# New Bioactive β-Resorcylic Acid Derivatives from the Alga-Derived Fungus *Penicillium antarcticum* KMM 4685

**DOI:** 10.3390/md21030178

**Published:** 2023-03-14

**Authors:** Elena V. Leshchenko, Alexandr S. Antonov, Gleb V. Borkunov, Jessica Hauschild, Olesya I. Zhuravleva, Yuliya V. Khudyakova, Alexander S. Menshov, Roman S. Popov, Natalya Yu Kim, Markus Graefen, Carsten Bokemeyer, Gunhild von Amsberg, Anton N. Yurchenko, Sergey A. Dyshlovoy

**Affiliations:** 1 G.B. Elyakov Pacific Institute of Bioorganic Chemistry, Far Eastern Branch of the Russian Academy of Sciences, 159 Prospect 100-letiya Vladivostoka, Vladivostok 690022, Russia; 2 Institute of High Technologies and Advanced Materials, Far Eastern Federal University, Vladivostok 690922, Russia; 3 Department of Oncology, Hematology and Bone Marrow Transplantation with Section Pneumology, Hubertus Wald Tumorzentrum—University Cancer Center Hamburg (UCCH), University Medical Center Hamburg-Eppendorf, Martinistrasse 52, 20246 Hamburg, Germany; 4 Martini-Klinik, Prostate Cancer Center, University Hospital Hamburg-Eppendorf, Martinistrasse 52, 20246 Hamburg, Germany

**Keywords:** marine-derived fungus, secondary metabolites, *Penicillium antarcticum*, p-glycoprotein inhibitory activity, prostate cancer, β-resorcylic acid

## Abstract

Five new β-resorcylic acid derivatives, 14-hydroxyasperentin B (**1**), β-resoantarctines A-C (**3**, **5**, **6**) and 8-dehydro-β-resoantarctine A (**4**), together with known 14-hydroxyasperentin (5′-hydroxyasperentin) (**2**), were isolated from the ethyl acetate extract of the fungus *Penicillium antarcticum* KMM 4685 associated with the brown alga *Sargassum miyabei*. The structures of the compounds were elucidated by spectroscopic analyses and modified Mosher’s method, and the biogenetic pathways for compounds **3**–**6** were proposed. For the very first time, the relative configuration of the C-14 center of a known compound **2** was assigned via analyses of magnitudes of the vicinal coupling constants. The new metabolites **3**–**6** were biogenically related to resorcylic acid lactones (RALs); however, they did not possess lactonized macrolide elements in their structures. Compounds **3**, **4** and **5** exhibited moderate cytotoxic activity in LNCaP, DU145 and 22Rv1 human prostate cancer cells. Moreover, these metabolites could inhibit the activity of p-glycoprotein at their noncytotoxic concentrations and consequently synergize with docetaxel in p-glycoprotein-overexpressing drug-resistant cancer cells.

## 1. Introduction

Marine-derived fungi are a rich source of promising lead molecules with various bioactive proprieties [1,2]. *Penicillium* species are among the most widespread fungal organisms on our planet. *Penicillium antarcticum* is a common species of micromycetes belonging to subgenus *Aspergilloides*, section *Canescentia*. Along with other representatives of the section *Canescentia*, *P. antarcticum* is a characteristic species of terrestrial and marine fungal assemblages and is often isolated from soils and substrates of plant and animal origin [3,4]. Currently, section *Canescentia* includes 23 species, of which 6 belong to the series of *Atroveneta*, including *P. antarcticum* [5]. Fungi of the *Canescentia* section are widespread and colonize various terrestrial and marine substrates, which was suggested to be due to their high metabolic activity, making representatives of this group promising sources of biologically active compounds [6]. Various compounds have been reported to be isolated from *P. canescens*. Among them, there are multioxidized aromatic polyketides penicanesins A–G [7], penicanesones A–C [8], canescones A–E [9], brominated azaphilones [10], tetrapeptide D-Phe-L-Val-D-Val-L-Tyr [11], polyketides antarones A and B [12] and cladomarine (asperentin B) [13,14].

Resorcylic acid lactones (RALs) are structurally diverse polyketides, which usually consist of condensed resorcylic and macrolide cycles [15]. Very recently, Bang and colleagues reported the very first non-lactonized RALs, i.e., possessing an opened macrolide cycle [16]. These compounds were isolated from the halophyte-associated marine fungus *Colletotrichum gloeosporioides* JS0419 [16]. RALs exhibit a broad range of biological activities, including anticancer activities [17,18]. To date, there are around 50 molecules belonging to this group for which a significant (IC_50_ < 10 µM) in vitro anticancer activity in human cancer cells has been shown (reviewed in [17]). Moreover, the main molecular targets of RALs in mammalian cells known to date are Hsp90, protein kinases and NF-kB [17]. Thus, zearalenone was shown to inhibit activity of the NF-κB transcriptional factor [19]; radicicol, pochonin D and other RALs could suppress Hsp-90 [18,20]. Various RALs were reported to irreversibly inhibit activity of several important protein kinases, which play a critical role in cancer-related MEK, ERK, RAS and RAF pathways [17]. Thus, activities of MEK, ERK, TAK, AKT, SRC, Aurora A and other kinases were reported to be suppressed by naturally derived RALs and their synthetic derivatives (reviewed in [17]). Additionally, radicicol A was identified as inhibitor of IL-1β, and some derivatives of pochonin D were shown to be VEGFR suppressors [17].

**Figure 1:**
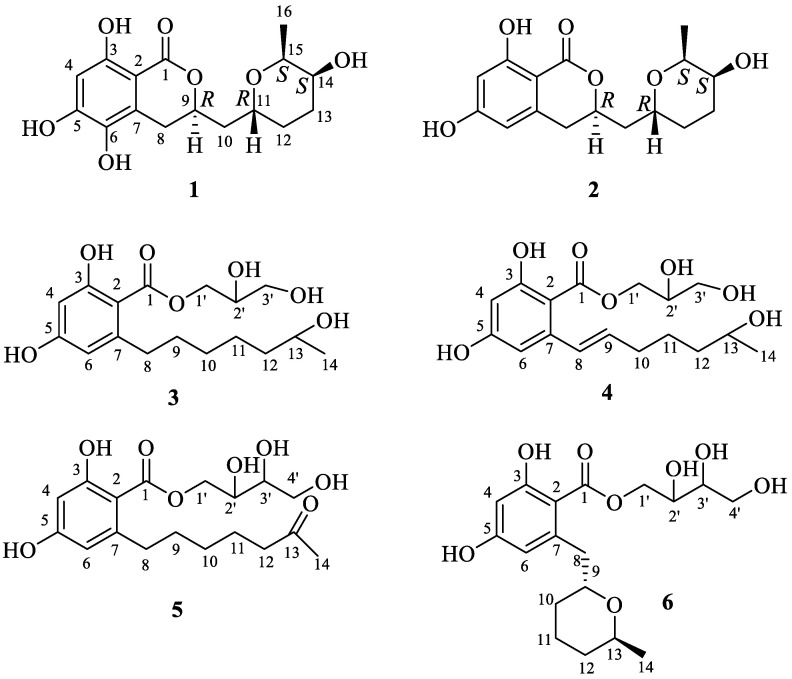
Metabolites isolated from *P. antarcticum*.

Recently, our group reported the isolation and characterization of three new bioactive meroterpenoids with rearranged skeletons, meroantarctines A–C, from the *Penicillium antarcticum* KMM 4685 [21]. Further searches for of new metabolites from this fungus strain resulted in the isolation of six β-resorcylic acid derivatives (**1**–**6**) (Figure 1). Herein, we report their isolation, structure elucidation, and activity in mammalian cells as well as the identification of the new type of biological activity for these RALs.

## 2. Results and Discussion

### 2.1. Structure Determination

The molecular formula of **2** was established as C_16_H_20_O_6_ from an HRESIMS peak at *m*/*z* 331.1153 [M + Na]^+^ with seven degrees of unsaturation. Using this result combined with the results of careful inspection of ^13^C and ^1^H NMR spectra (Table 1), including HSQC, HMBC, COSY and DEPT experiments, as well as comparison with literature data [22,23], the compound **2** was established as known 14-hydroxyasperentin (5′-hydroxyasperentin).

**Figure 2:**
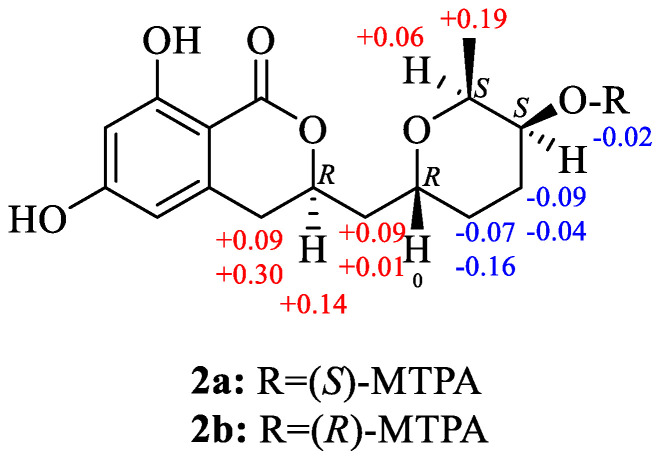
Δδ (δS − δR) values (in ppm) for the (S) − and (R) −MPTA esters of **2**.

According to the previously published data, the relative configuration of C-14 (C-5′) chiral center of compound **2** suggests its *β*-configuration; however, the authors of the primary publication did not provide any spectral data or other proofs supporting this speculation [23]. In the other reports the relative configuration of the C-5′ chiral center of 5′-hydroxyasperentin has not been established [22,24,25,26,27]. The ROESY spectrum of **2** did not contain any cross-peaks, which can be useful to establish the relative stereochemistry of **2**. However, the thorough analysis of the vicinal coupling constants for H-15 (δ_H_ 3.96, *J* = 6.6, 4.2 Hz) and H-14 (δ_H_ 3.70, *J* = 9.0, 4.3 Hz) together with biogenetic considerations of cladosporin biosynthesis and their derivatives [23,26] suggested a *β*-configuration of the methyl group at C-15 and a β-configuration of the hydroxy group at C-14. Esterification of **2** with (*R*)- and (*S*)-MTPA chloride occurred at the C-14 hydroxy group to give the (*S*)- and (*R*)-MTPA esters **2a** and **2b**, respectively. The observed chemical shift differences Δδ (δ*S* − δ*R*) (Figure 2) indicated the 14*S* configuration. Taken together, these data indicate the absolute stereostructure of **2** to be 9*R*,11*R*,14*S*,15*S*. 

**Figure 3:**
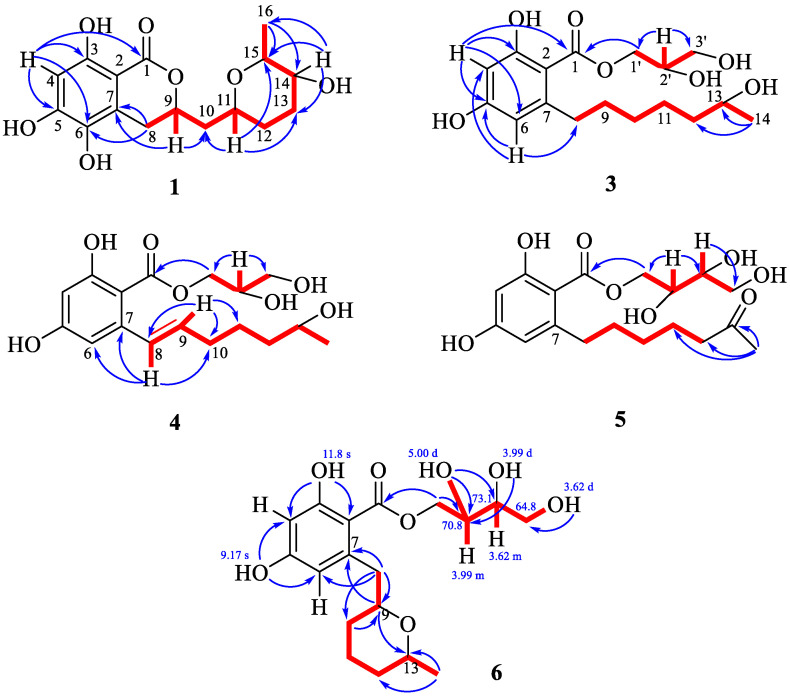
Key HMBC (blue arrows) and COSY (bold lines) correlations of **1** and **3**–**6**.

The molecular formula of **1** was established to be C_16_H_20_O_7_, which was indicated by a HRESIMS peak at *m*/*z* 347.1103 [M + Na]^+^ with seven degrees of unsaturation. A careful inspection of ^13^C NMR (Table 1), DEPT and HSQC data of **1** revealed the presence of one methyl group (δ_H_ 1.20, δ_C_ 13.1), four methylenes (δ_C_ 27.2, δ_C_ 28.6, δ_C_ 30.0, δ_C_ 40.5), five methines (δ_H_ 6.26, δ_C_ 101.8; δ_H_ 4.61, δ_C_ 77.7; δ_H_ 4.00, δ_C_ 66.5; δ_H_ 3.70, δ_C_ 69.0; δ_H_ 3.96, δ_C_ 72.3), five *sp^2^* quaternary carbons (δ_C_ 100.2; δ_C_ 126.1; δ_C_ 135.7; δ_C_ 155.6; δ_C_ 159.1) and a carbonyl group (δ_C_ 171.8). The ^1^H and ^13^C NMR data observed for **1** closely resembled those obtained for asperentin B [13,14] except for the C-13 and C-14 carbon and H_2_-13 and H-14 proton signals. The HMBC correlations H_3_-16 (δ_H_ 1.20)/C-15 (δ_C_ 72.3) and C-14 (δ_C_ 69.0); H-14 (δ_H_ 3.70)/C-15 and C-13 (δ_C_ 27.2) established the location of CH_3_-16 and 14-OH groups (Figure 3). Compound **1** was named 14-hydroxyasperentin B (the numeration of atoms was assigned as in the original papers [13,14]). Similar to compound **2**, the ROESY spectrum of **1** could not be used to determine the stereoconfigurations of **1**. However, based on the obvious biogenetic similarity, the identity of the chemical shift values of compounds **1** and **2** (Table 1) and the specific optical rotation values of [α]_D_^20^—15.5 (for **1**) and [α]_D_^20^—14.1 (for **2**) were found, and the absolute configurations of the stereogenic centers of **1** were assigned as 9*R*,11*R*,14*S*,15*S.*

The molecular formula of **3** was established to be C_17_H_26_O_7_ from an HRESIMS peak at *m*/*z* 365.1571 [M + Na]^+^ and confirmed by ^13^C NMR spectrum. An extensive analysis of ^13^C NMR (Table 2) and ^1^H NMR (Table 3), including DEPT and HSQC experiments of **3** revealed the presence of one methyl (δ_H_ 1.13, δ_C_ 23.5) group, seven methylenes (δ_C_ 26.8, δ_C_ 30.8, δ_C_ 33.1, δ_C_ 37.3, δ_C_ 40.1) including two oxygen-bearing methylenes (δ_H_ 3.62, 3.60, δ_C_ 64.5; δ_H_ 4.41, 4.30, δ_C_ 67.2), four methines (δ_H_ 6.15, δ_C_ 102.6; δ_H_ 6.20, δ_C_ 111.7) including two oxygen-bearing methines (δ_H_ 3.70, δ_C_ 68.6 and δ_H_ 3.96, δ_C_ 71.1), four *sp^2^* quaternary carbons (δ_C_ 106.0; δ_C_ 165.0; δ_C_ 163.0; δ_C_ 149.2) and one carbonyl group (δ_C_ 172.4). From these data, the five degrees of unsaturation from the molecular formula suggested compound **3** possessed one aromatic ring and one carbonyl group. HMBC correlations (Figure 3) from H-4 (δ_H_ 6.15) to C-1 (δ_C_ 172.4), C-2 (δ_C_ 106.0), C-3 (δ_C_ 165.0), C-5 (δ_C_ 163.0) and C-6 (δ_C_ 111.7), and from H-6 (δ_H_ 6.20) to C-1, C-2, C-4 (δ_C_ 102.6) and C-5, revealed the presence of a β-resorcylic acid moiety (2,4-dihydroxybenzoic acid). The ^1^H-^1^H COSY and HSQC spectra of **3** revealed the partial connectivity sequence of the following protons as CH_2_(1′)−CH(2′)−CH_2_(3′) and HMBC correlations from H-2′ (δ_H_ 3.96) to C-1′ (δ_C_ 64.5) and C-3′ (δ_C_ 67.2), as well as from H_2_-1′ (δ_H_ 4.41, 4.30) to C-1, C-2′ (δ_C_ 71.1) and C-3′, established the 1,2-dihydroxypropan ester residue (C-1′–C-3′ residue numbering) at C-1. The partially connectivity sequence of the protons as CH_2_(8)−CH_2_(9)−CH_2_(10)−CH_2_(11)−CH_2_(12)−CH(13)−CH_3_(14) and HMBC correlations from H_2_-8 (δ_H_ 2.88, 2.84) to C-2, C-6, C-7 (δ_C_ 149.2) and C-10 (δ_C_ 30.8); from H_2_-9 (δ_H_ 1.58, 1.55) to C-7, C-10 and C-11 (δ_C_ 26.8); from H_2_-10 (δ_H_ 1.38) to C-9, C-11 and C-12 (δ_C_ 40.1); from H_2_-11 (δ_H_ 1.39) to C-9 and C-13 (δ_C_ 68.6); from H_2_-12 (δ_H_ 1.41) to C-10, C-11 and C-13; and from H_3_-14 (δ_H_ 1.13) to C-12 and C-13 indicated the presence of heptan-2-ol residue at C-7 and methyl group CH_3_-14 at C-13. Note that the magnitudes of the vicinal coupling constants for H-2′ from the data of the ROESY experiment were not informative. Thus, the planar structure of compound **3** named β-resoantarctine A was established. 

The molecular formula of **4** was established to be C_17_H_24_O_7_ from an HRESIMS peak at *m*/*z* 363.1416 [M + Na]^+^ that corresponds to six degrees of unsaturation. The general features of the ^13^C NMR of **4** resembled those of **3** with the exception of the C-6–C-10 carbon signals (Table 2). The analysis of ^1^H-^1^H COSY spectrum of **2** revealed the sequence of following protons formed an isolated spin system: H(8)−H(9)−H_2_(10)−H_2_(11)−H_2_(12)−H(13)−H_3_(14) (Figure 3). These data together with HMBC correlations from H-8 (δ_H_ 6.98) to C-2, C-6, C-7 (δ_C_ 144.9) and C-10 (δ_C_ 34.0); from H-9 (δ_H_ 5.91) to C-7, C-10 (δ_C_ 34.0) and C-11 (δ_C_ 26.6); from H_2_-10 (δ_H_ 2.24) to C-9, C-11 and C-12 (δ_C_ 39.7); from H_2_-11 (δ_H_ 1.60, 1.52) to C-9 and C-13 (δ_C_ 68.4); from H-12b (δ_H_ 1.45) to C-10, C-11 and C-13; and from H_3_-14 (δ_H_ 1.16) to C-12 and C-13 indicated the hept-6-en-2-ol residue (C-8–C-14 residue numbering) bonded to C-7. The magnitudes of the vicinal coupling constants for H-8 (δ_H_ 6.98, *J* = 15.5 Hz) and H-9 (δ_H_ 5.91, *J* = 15.5 Hz) together with chemical shifts for C-8 (δ_C_ 132.3) and C-9 (δ_C_ 133.6) in the ^13^C NMR indicated the *trans*-configuration of Δ^8^ double bond. However, it was impossible to establish the relative configurations of C-2′ and C-13 chiral centers because the magnitudes of the coupling constants for H-2′ and H-13 and the data of the ROESY experiment were not informative. Thus, the planar structure of compound **4**, named 8-dehydro-β-resoantacrtine A, was established.

The molecular formula of **5** was established to be C_18_H_26_O_8_ from an HRESIMS peak at *m*/*z* 393.1519 [M + Na]^+^ that corresponds to six degrees of unsaturation. The general features of the ^13^C NMR of **5** resembled those of **4** with the exception of the C-13, C-14, C-3′ and additional C-4′ carbon signals (Table 2). ^1^H-^1^H COSY and HSQC spectra of **5** revealed the partially connectivity sequence of the protons as CH_2_(1′)−CH(2′)−CH(3′)−CH_2_(4′) as seen above (Figure 3). This information together with the HMBC correlation from H-2′ (δ_H_ 3.88) to C-1′ (δ_C_ 68.1) and C-3′ (δ_C_ 73.8); from H_2_-1′ to C-1 (δ_C_ 172.5), C-2′ (δ_C_ 71.3) and C-3′; and from H-3′ to C-1′, C-2′ and C-4′ (δ_C_ 64.6) allowed us to establish the 1,2,3-trihydroxybutan ester residue (C-1′–C-4′ residue numbering) linked to C-1. ^1^H-^1^H COSY and HSQC spectra of **5** revealed the partial connectivity sequence of the protons as CH_2_(8)−CH_2_(9)−CH_2_(10)−CH_2_(11)−CH_2_(12) as shown above. The HMBC correlations from H_3_-14 (δ_H_ 2.11) to C-13 (δ_C_ 209.0) and C-12 (δ_C_ 44.3), and from H_2_-8 (δ_H_ 2.90, 2.84) to C-2 (δ_C_ 106.2), C-6 (δ_C_ 111.7), C-7 (δ_C_ 149.1) and C-10 (δ_C_ 30.2), indicated the heptane-2-one residue (C-8–C-14 residue numbering) was bonded to C-7. The assigning of relative configurations of C-2′ and C-3′ stereocenters was impossible on the base of magnitudes of the coupling constants for H-2′ and H-3′, and the data of the ROESY experiment were not informative. Thus, the planar structure of compound **3**, named β-resoantarctine B, was established. 

**Figure 4:**
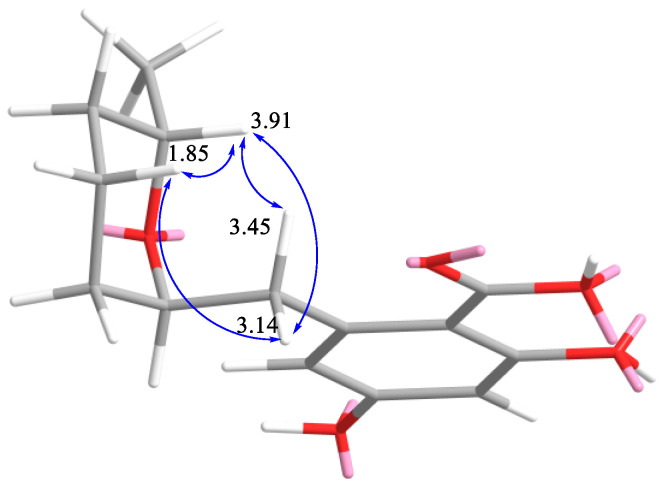
Key ROESY (blue arrows) correlations of **6** (part of a molecule is shown).

The molecular formula of **6** was established to be C_18_H_26_O_8_ from an HRESIMS peak at *m*/*z* 393.1524 [M + Na]^+^ and was supported by the ^13^C NMR spectrum. A close inspection of ^13^C NMR (Table 2) and ^1^H NMR (Table 3), including DEPT and HSQC experiments of **6,** revealed the presence of one methyl (δ_H_ 1.13, δ_C_ 21.3) group, six methylenes (δ_C_ 18.7, δ_C_ 27.2, δ_C_ 33.3, δ_C_ 39.9) including two oxygen-bearing methylenes (δ_H_ 3.80, 3.66, δ_C_ 64.8; δ_H_ 4.62, 4.33, δ_C_ 70.0), six methines (δ_H_ 6.28, δ_C_ 102.4; δ_H_ 6.28, δ_C_ 113.2) including two oxygen-bearing methines (δ_H_ 3.62, δ_C_ 73.1; δ_H_ 3.91, δ_C_ 67.8; δ_H_ 3.99, δ_C_ 70.8 and δ_H_ 4.03, δ_C_ 74.2), four *sp^2^* quaternary carbons (δ_C_ 105.1; δ_C_ 168.8; δ_C_ 163.2; δ_C_ 145.4) and one carbonyl group (δ_C_ 172.4). Additionally, thee ^1^H NMR spectra revealed the presence three doublets (δ_H_ 5.00 (1H, d, *J* = 5.3), δ_H_ 3.99 (1H, d, *J* = 5.8) and δ_H_ 3.62 (1H, d, *J* = 5.4) without any correlations in HSQC experiments. Some part of the signals in the ^13^C NMR and ^1^H NMR spectra of **6** were close to those in **5** with the exception of the C-7 (δ_C_ 145.4), C-8 (δ_C_ 39.9), C-9 (δ_C_ 74.2), C-10 (δ_C_ 27.2), C-11 (δ_C_ 18.7), C-12 (δ_C_ 33.3), C-13 (δ_C_ 67.8) and C-14 (δ_C_ 21.3) carbon signals (Table 2). ^1^H-^1^H COSY and HSQC spectra of **6** revealed the partial connectivity sequence of the protons as CH_2_(8)−CH(9)−CH_2_(10)−CH_2_(11)−CH_2_(12)−CH(13)−CH_3_(14) and CH_2_(1′)−CH(2′)−CH(3′)−CH_2_(4′) (Figure 3). These data showed the molecular formula corresponded to six degrees of unsaturation, the deshielding of the signal of H-9 to δ_H_ 4.03 and of the corresponding carbon signal (C-9) to δ_C_ 74.2 as well as HMBC correlations from H-9 (δ_H_ 4.03) to C-7 and C-13, which allowed us to suppose the presence of the ring B at C-8. HMBC correlations from the hydroxyl groups from (δ_H_ 5.00) to C-2′ and C-3′; from (δ_H_ 3.99) to C-2′, C-3′ and C-4′; and from (δ_H_ 5.00) to C-4′ indicated their locations at C-2′, C-3′ and C-4′, respectively. The observed ROESY correlations from H-13 (δ_H_ 3.91) to H_2_-8 (δ_H_ 3.45, δ_H_ 3.14) and H_a_-11 (δ_H_ 1.85) and from H_b_-8 (δ_H_ 3.14) to H_a_-11 (Figure S63) indicated axial location of H_a_-11 and H-13 and substituent at C-9 (Figure 4). This information revealed the β-configuration of H_3_-14 and H-9. 

**Figure 5:**
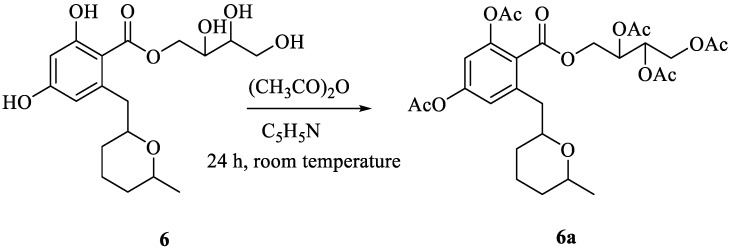
Scheme for the preparation of pentaacetate β-resoantarctine C **6a**.

The planar structure of compound **6**, named β-resoantarctine C, was also confirmed by the obtaining its pentaacetate derivative **6a** (Figure 5) via acylation reaction. The molecular formula of **6a** was established as C_28_H_36_O_13_ from a HRESIMS peak at *m*/*z* 603.2051 [M + Na]^+^ and was supported by the analysis of ^13^C NMR data (Figure S45).

**Figure 6:**
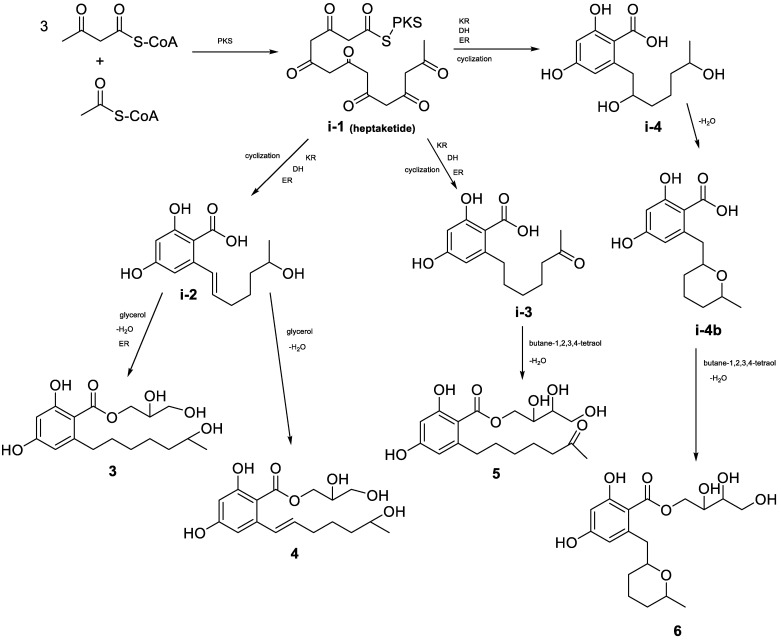
Proposed biosynthetic pathway for compounds **3**–**6**. PKS—polyketide synthase; KR—keto reductase; DH—dehydratase; ER—enoyl reductase.

Previously, RALs biosynthetic pathways were investigated in detail in several studies [15,18]. The formation polyketide precursor, which is catalyzed by polyketide synthase, is followed by the further cyclization to β-resorcylic acid [15]. Ultimately this results in the formation of a hybrid structure consisting of 2,4-dihydroxybenzoic acid moiety and a macrolide ring [15]. Usually, the β-resorcylic acid residue has an aliphatic side chain that is attached at C-7 and esterified with the C-1 carboxylic acid, therefore forming a macrolactone ring [15]. However, in the isolated compounds **3**–**6,** the glycerol and butane-1,2,3,4-tetraol were esterified with the C-1 carboxylic acid, therefore forming non-lactonizes β-resorcylic acid derivatives. Thus, our study is (i) the very first report on non-lactonizes RALs having polyol esterified at C-1 and (ii) the second report on β-resorcylic acid harboring a long unesterified aliphatic side chain [16]. Based on this, for the isolated compounds **3−6** we suggest a biosynthetic pathway that includes the same steps as previously reported fungal RALs, but without a macrolide ring formation (Figure 6).

### 2.2. Activity and Selectivity in Prostate Cancer Cells

Previously, RALs have been reported to exhibit cytotoxic activity in human cancer cell lines in vitro [17]. This effect has been stipulated by Hsp90, protein kinase (PK) and NF-kB inhibition. Moreover, the 14-membered RALs were the most potent among the investigated molecules regarding anticancer activity [17]. Therefore, we examined cytotoxic activity of the three new isolated RALs (**3**–**6**) using human prostate cancer cells bearing various levels of drug resistance. Hence, we used PC-3 and DU145 cells, which do not express the full-length androgen receptor (AR) and therefore are non-sensitive to hormonal therapy; docetaxel- and hormonal therapy-resistant PC3-DR cells; 22Rv1 and VCaP cells, which are also insensitive to hormonal therapy due to expression of the AR splice variant V7 (AR-V7) resulting in a ligand-independent AR pathway activation; and hormone-sensitive LNCaP cells, which express the wild-type AR (full-length AR).

**Figure 7:**
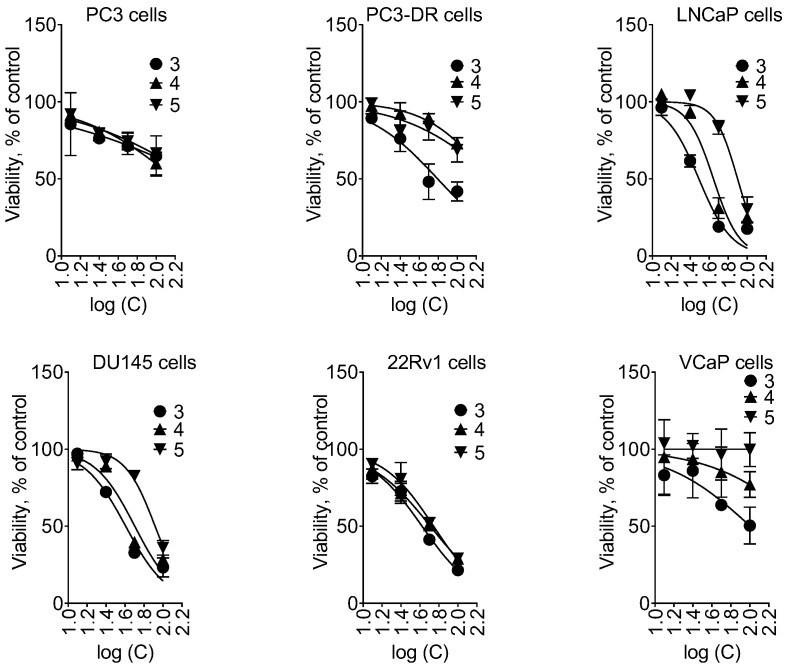
Viability of the prostate cancer cells treated with the isolated compounds **3**–**5**. The viability was measured using MTT assay following 48 h of treatment. The values are shown as mean ± SD.

Overall, compounds **3**, **4** and **5** exhibited moderate cytotoxic activity in LNCaP, DU145 and 22Rv1 cells, whereas they were inactive in PC3 and VCaP cells up to 100 µM (Table 4, Figure 7). 

Interestingly, compound **3** was active in docetaxel-resistant PC3-DR cells, whereas no activity in PC3 cells was observed (Table 4).

**Figure 8:**
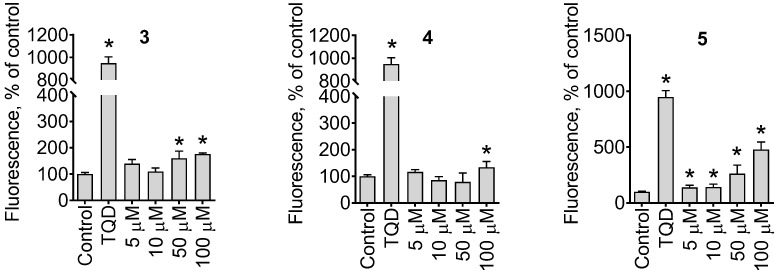
Effect on p-glycoprotein activity. The p-gp-overexpressing PC3-DR cells were treated with 5–100 µM of the tested compounds for 30 min following incubation with calcein-AM for 15 min. Additionally, 50 nM tariquidar (TQD) was used as a positive control. Significant difference from control is indicated as * (*p* < 0.05, one-way ANOVA).

We further examined the effect of the isolated compounds on the activity of p-glycoprotein (p-gp). Overexpression of p-gp has been identified to be a major mechanism of drug resistance in cancer cells. It mediates the excretion of various chemotherapeutic agents, including taxanes, out of the cancer cell. To evaluate the effect of the isolated compounds on p-gp activity, we utilized the calcein exclusion assay and used p-gp overexpressing docetaxel-resistant PC3-DR cells as a model [28,29]. Calcein-AM is a non-fluorescent small molecule that can passively enter the cells via diffusion. In the cytoplasm, calcein-AM is hydrolyzed to release green fluorescent-free calcein, which can be then detected. Calcein and calcein-AM are known substrates for p-gp. Therefore, in cells overexpressing p-gp, non-hydrolyzed and therefore non-fluorescent calcein-AM is quickly evacuated back into the extracellular space, and no fluorescence can be observed. The inhibitors of p-gp, such as tariquidar, can block the p-gp-mediated drug excretion and therefore promote the therapeutic effects of the chemotherapeutics. Remarkably, we examined the p-gp inhibitory activity of compounds **3**–**5**. For compounds **3** and **4,** moderate p-gp inhibitory activity was observed only at high concentrations of ~100 µM, whereas for carbonyl-containing compound **5,** significant inhibitory activity was detected at the low concentrations of 5 µM (Figure 8). 

**Figure 9:**
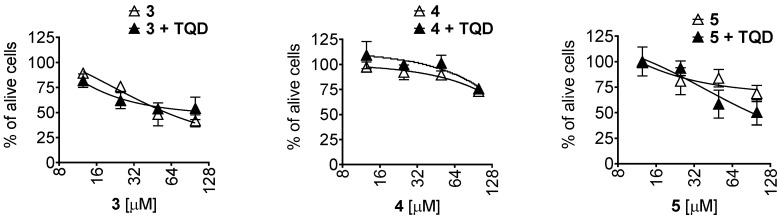
Effect of tariquidar on cytotoxicity of the isolated compounds in PC3-DR cells. The cells were pretreated with tariquidar (TQD, 50 nM) for 1 h and then co-treated with the tested compounds for 48 h. The viability was measured using MTT assay.

Accumulation of calcein in the cells results from p-gp inhibition, which may be either due to the p-gp blockade by its true inhibitor or by a concurrent substrate molecule. In order to identify whether the tested compounds are p-gp substrates (which ultimately lead to concurrent inhibition of calcein excretion from the cells), we examined the effect of well-established p-gp inhibitor tariquidar (TQD) on cytotoxic activity of the compounds in p-gp-overexpressing PC3-DR cells. Thus, co-treatment with 50 nM TQD did not result in any significant alteration of cytotoxic activity of the isolated compounds (Figure 9), while the activity of docetaxel (a clinically approved chemotherapeutic drug that is known to be a p-gp substrate) was dramatically increased [28]. Therefore, we concluded that unlike to docetaxel, the isolated compounds **3**–**5** are not p-gp substrates, but rather the true p-gp inhibitors.

**Figure 10:**
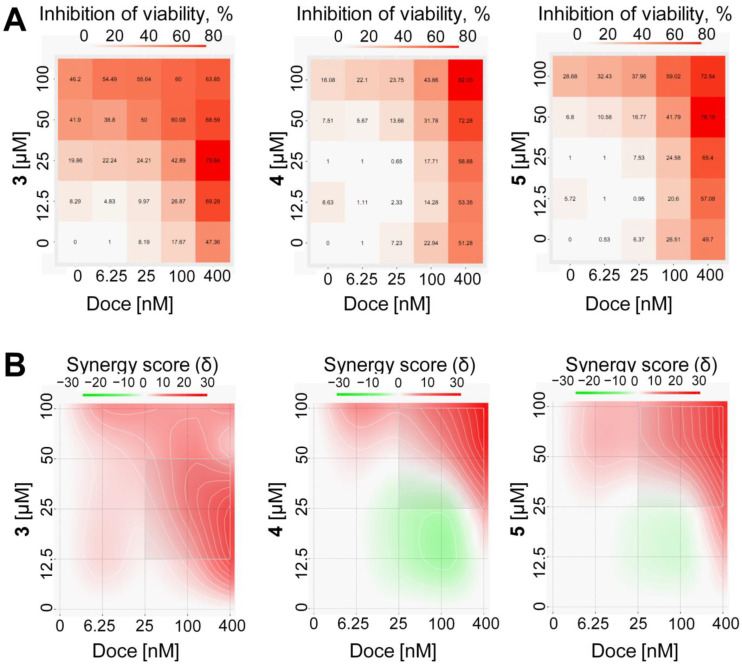
Effects of the drug combinations with docetaxel in PC3–DR cells. Cells were treated with the tested compounds, docetaxel (Doce) and their combinations at the indicated concentrations. The viability was measured after 48 h of treatment using MTT assay. The cytotoxic activity heat-maps (**A**) and synergistic 2D maps (**B**) were constructed and analyzed using SynergyFinder 2.0 software. Red areas indicate synergistic effect of the drug combinations (**B**).

A high tolerance of the cells to this chemotherapeutic agent is due to p-gp-mediated excretion of the drug [28,29]. Hence, inhibition of p-gp activity should result in the accumulation of docetaxel within the cells, leading to the more pronounced cytotoxic activity of this drug. Therefore, we co-treated PC3-DR cells with docetaxel plus the isolated compounds **3**–**5**. Further measurement of cellular viability followed by data analysis with the SynergyFinder 2.0 software using the ZIP model indicated a synergistic effect of the combinational treatment, especially at a high concentration of both agents (Figure 10). These findings are in line with the above-reported p-gp inhibitory activity of the isolated compounds **3**–**5** (Figure 10A,B).

## 3. Materials and Methods

### 3.1. General Experimental Procedures

Optical rotations were measured on a Perkin-Elmer 343 polarimeter (Perkin Elmer, Waltham, MA, USA) in MeOH. UV spectra were recorded on a Shimadzu UV-1601PC spectrometer (Shimadzu Corporation, Kyoto, Japan) in MeOH. ECD spectra were measured using a Chirascan-Plus CD Spectrometer (Leatherhead, UK) in MeOH. ^1^H and ^13^C NMR spectra were recorded in aceton-d_6_ on Bruker Avance-500, Avance III-700 and Bruker DPX-300 spectrometers (Bruker BioSpin GmbH, Rheinstetten, Germany) operating at 500 MHz and 125 MHz, 700 and 176 MHz and 300 and 75 MHz, respectively, using TMS as an internal standard. HRESIMS spectra were obtained using Bruker maXis Impact II mass spectrometer (Bruker Daltonics GmbH, Rheinstetten, Germany). 

Low-pressure liquid column chromatography was performed using Si gel KSK (50/100 μm, Imid Ltd., Krasnodar, Russia) and Gel ODS-A (12 nm, S—75 um, YMC Co., Ishikawa, Japan). Plates precoated with Si gel (5–17 μm, 4.5 × 6.0 cm, Imid) and Si gel60 RP-18 F254S (20 × 20 cm, Merck KGaA, Darmstadt, Germany) were used for thin-layer chromatography. Preparative HPLC was carried out on a Shimadzu LC-20 (Shimadzu, Kyoto, Japan) and Agilent 1100 (Agilent Technologies, Santa Clara, CA, USA) chromatographs using a Shimadzu RID-20A and Agilent 1100 refractometers and YMC ODS-AM (YMC Co, 5 μm, 250 × 10 mm), Synergi, Fusion-RP (Phenomenex, Torrance, CA, USA, 4 μm, 250 × 10 mm) and HyperClone ODS (Phenomenex, 5 μm, 250 × 4.6 mm) columns.

### 3.2. Fungal Strain 

The strain of the fungus *Penicillium antarcticum* KMM 4685 was isolated from superficial mycobiota of the marine brown alga *Sargassum miyabei* (Sea of Japan) (GenBank sequence dataset and registered under accession number MW527122) [21]. The strain is stored at the Collection of Marine Microorganisms (KMM) of G.B. Elyakov Pacific Institute of Bioorganic Chemistry (Vladivostok, Russia).

### 3.3. Cultivation of P. thomii

The fungus was grown stationary at 22 °C for 21 days in 100 Erlenmeyer flasks (500 mL), each containing 20 g of rice, 20 mg of yeast extract, 10 mg of KH_2_PO_4_ and 40 mL of natural sea water (Marine Experimental Station of G.B. Elyakov Pacific Institute of Bioorganic Chemistry, Troitsa (Trinity) Bay, Sea of Japan). 

### 3.4. Extraction and Isolation 

At the end of the incubation period, the mycelia and medium were homogenized and extracted with EtOAc (1 L). The obtained extract was dried in vacuo. The residue was dissolved in H_2_O−EtOH (4:1) (300 mL) and was extracted with *n*-hexane (0.2 L × 3) and EtOAc (0.2 L × 3). After evaporation of the EtOAc layer, the residual material (5 g) was passed over a silica column (6 × 15 cm), which was eluted followed by a step gradient from 10:1 to 6:1 EtOH in CHCl_3_ (total volume 5 L). Fractions of 250 mL were collected and combined based on TLC results (Si gel, toluene–isopropanol 6:1 and 3:1, *v*/*v*). As a result, two fractions were obtained: SM-1327-3 (1.87 g) and SM-1327-4 (640 mg). 

Fraction SM-1327-3 (1.87 g) was purified on a Si Gel column (32 × 2 cm), which was eluted first with n-hexane (200 mL) followed by a step gradient from 5% to 50% EtOAc in *n*-hexane (total volume: 5 L). Fractions of 250 mL were collected and combined on the basis of the TLC results (Si gel, toluene–isopropanol 6:1 and 3:1, *v*/*v*). 

The *n*-hexane–EtOAc (50:50) (100 mg) eluate was purified on a Gel ODS-A column eluting with MeOH–H_2_O (100:0) to yield the subfraction SM-1327-3-5 (91 mg), which was purified on a YMC-ODS-AM column eluting with MeOH–H_2_O (80:20) to yield subfractions SM-1327-3-5-1 (61 mg). Subfraction SM-1327-3-5-1 (61 mg) was purified on a YMC-ODS-AM column eluting with MeOH−H_2_O (60:40) to yield SM-1327-3-5-1-7 (27.1 mg), which was purified on a HyperClone column eluting with CH_3_CN−H_2_O (30:70) to yield **6** (1.4 mg).

Fraction SM-1327-4 (640 mg) was purified on a Gel ODS-A column (8 × 15 cm) eluting with MeOH–H_2_O (50:50, 80:20, 100:0) to yield purified SM-1327-4 (580 mg) (MeOH–H_2_O, 50:50). Purified SM-1327-4 (580 mg) was purified on a YMC ODS-AM column eluting with MeOH–H_2_O (80:20) to yield subfractions SM-1327-4(3) (53.2 mg) and SM-1327-4(5) (27.0 mg). 

Subfraction SM-1327-4(5) (27.0 mg) was purified on a fusion column eluting with CH_3_CN–H_2_O (30:70) to yield **2** (3.9 mg), **3** (4.2 mg) and **4** (2.2 mg). 

Subfraction SM-1327-4(3) (53.2 mg) was purified on a YMC-ODS-AM column eluting with MeOH–H_2_O (60:40) to yield subfraction SM-1327-4(3)-3 (12.7 mg). Subfraction SM-1327-4(3)-3 (12.7 mg) was purified on a fusion column eluting with CH_3_CN–H_2_O (30:70) to yield **1** (2.1 mg) and **5** (1.4 mg). 

### 3.5. Spectral Data

14-hydroxyasperentin B (**1**): colorless amorphous; [α]_D_ ^20^ –15.5 (c 0.18 MeOH); UV (MeOH) λ_max_ (log ε) 234 (4.99), 223 (3.92), 272 (3.89), 329 (3.72) and 209 (3.50) nm; CD (c 0.00069 M, MeOH), λ_max_ (∆ε) 200 (+4.75), 224 (−2.67), 242 (+2.47) and 272 (+1.45) nm; ^1^H and ^13^C NMR data, see Table 1, Supplementary Figures S2–S7; HRESIMS [M + Na]^+^ *m*/*z* 347.1103 (calcd. for C_16_H_20_O_7_Na 347.1101, ∆−0.4 ppm), [M − H]^−^ *m*/*z* 323.1136 (calcd. for C_16_H_19_O_7_ 323.1136, ∆0.2 ppm) (Figure S1).

14-hydroxyasperentin (**2**): colorless amorphous; [α]_D_ ^20^ –14.1 (c 0.17 MeOH); UV (MeOH) λ_max_ (log ε) 213 (4.00), 269 (3.54) and 301 (3.19) nm; CD (c 0.000173 M, MeOH), λ_max_ (∆ε) 225 (−2.48), 235 (+3.41), 248 (+0.79) and 270 (4.33) nm; ^1^H and ^13^C NMR data, see Table 1, Supplementary Figures S9–S14; HRESIMS [M + Na]^+^ *m*/*z* 331.1153 (calcd. for C_16_H_20_O_6_Na 331.1152, ∆−0.3 ppm), [M − H]^−^ *m*/*z* 307.1182 (calcd. for C_16_H_19_O_6_ 307.1187, ∆1.6 ppm) (Figure S8).

β-resoantarctine A (**3**): colorless amorphous; [α]_D_ ^20^ + 2.94 (c 0.17 MeOH); UV (MeOH) λ_max_ (log ε) 218 (4.37), 265 (4.09) and 302 (3.69) nm; CD (c 0.00053 M, MeOH), λ_max_ (∆ε) 211 (−0.56) and 253 (−0.07) nm; ^1^H and ^13^C NMR data, see Table 1 and Table 2, Supplementary Figures S16–S21; HRESIMS [M + Na]^+^ *m*/*z* 365.1571 (calcd. for C_17_H_26_O_7_Na 365.1571, ∆−0.1 ppm), [M − H]^−^ *m*/*z* 341.1605 (calcd. for C_17_H_25_O_7_ 341.1606, ∆0.1 ppm) (Figure S15).

8-dehydro-β-resoantarctine A (**4**): colorless amorphous; [α]_D_ ^20^ + 3.13 (c 0.16 MeOH); UV (MeOH) λ_max_ (log ε) 235 (4.29), 271 (4.00) and 309 (3.65) nm; CD (c 0.00059 M, MeOH), λ_max_ (∆ε) 218 (−0.43), 224 (−0.49), 247 (0.06) and 270 (0,15) nm; ^1^H and ^13^C NMR data, see Table 1 and Table 2, Supplementary Figures S23–S28; HRESIMS [M + Na]^+^ *m*/*z* 363.1416 (calcd. for C_17_H_24_O_7_Na 363.1414, ∆−0.5 ppm), [M − H]^−^ *m*/*z* 339.1447 (calcd. for C_17_H_23_O_7_ 339.1449, ∆0.6 ppm) (Figure S22).

β-resoantarctine B (**5**): colorless amorphous; [α]_D_ ^20^–3.33 (c 0.15 MeOH); UV (MeOH) λ_max_ (log ε) 235 (4.29), 271 (4.00) and 309 (3.65) nm; CD (c 0.00059 M, MeOH), λ_max_ (∆ε) 218 (−0.43), 224 (−0.49), 247 (+0.06) and 270 (+0.15) nm; ^1^H and ^13^C NMR data, see Table 1 and Table 2, Supplementary Figures S30–S35; HRESIMS [M + Na]^+^ m/z 393.1519 (calcd. for C_18_H_26_O_8_Na 393.1520, ∆0.3 ppm), [M − H]^-^ *m*/*z* 369.1550 (calcd. for C_18_H_25_O_8_ 369.1555, ∆1.3 ppm) (Figure S29).

β-resoantarctine C (**6**): colorless amorphous; [α]_D_ ^20^–56.0 (c 0.05 MeOH); UV (MeOH) λ_max_ (log ε) 219 (3.75), 265 (3.47) and 303 (3.116) nm; CD (c 0.00061 M, MeOH), λ_max_ (∆ε) 208 (−11.22), 229 (−2.66), 262 (+2.98) and 303 (+0.84) nm; ^1^H and ^13^C NMR data, see Table 1 and Table 2, Supplementary Figures S37–S42; HRESIMS [M + Na]^+^ m/z 393.1524 (calcd. for C_18_H_26_O_8_Na 393.1520, ∆−0.9 ppm), [M − H]^−^ *m*/*z* 369.1561 (calcd. for C_18_H_26_O_8_ 369.1555, ∆−1.7 ppm) (Figure S36).

### 3.6. Preparation of Pentaacetate β-Resoantarctine C (***6a***)

To a solution of **6** (0.4 mg) in 100 μL pyridine, acetic anhydride was added at room temperature, and the mixture was stirred for 24 h without UV.

Pentaacetate β-resoantarctine C (**6a**): colorless amorphous; ^1^H and ^13^C NMR data, Supplementary Figures S44–S57; HRESIMS [M + Na]^+^ *m*/*z* 603.2051 (calcd. for C_28_H_36_O_13_Na 603.2048, ∆−0.4 ppm) (Figure S43).

### 3.7. Preparation of (S)-MTPA and (R)-MTPA Esters of ***2***

To a solution of **2** (1 mg) in pyridine, 4-dimethylaminopyridine (a few crystals) and (R)-MTPA-Cl (1.2 μL) were added at room temperature, and the mixture was stirred for 1.5 h. After evaporation of the solvent, the residue was purified by HPLC on a HyperClone column eluting with CH_3_CN−H_2_O (70:30) to afford the (S)-MTPA ester of **2** (**2a**). The (R)-MTPA ester of **2** (**2b**) was prepared similarly manner using (S)-MTPA-Cl. NMR data of (R, S)-MTPA esters of **2** can be found in Supplementary Figures S53–S56. HRESIMS (**2a**) *m*/*z* 763.1940 [M + Na]^+^ (calcd. for C_36_H_34_F_6_O_10_Na, 763.1948), *m*/*z* 739.1951 [M − H]^−^ (calcd. for C_36_H_33_F_6_O_10_, 739.1983) and HRESIMS (**2b**) *m*/*z* 979.2330 [M + Na]^+^ (calcd. for C_46_H_41_F_9_O_12_Na, 979.2347).

### 3.8. Reagents and Antibodies for Biological Experiments

Calcein-AM was purchased from BIOZOL (Eching, Germany), Tariquidar was purchased from MedChemExpress (Monmouth Junction, NJ, USA), MTT (3-(4,5-dimethylthiazol-2-yl)-2,5-diphenyltetrazolium bromide) was purchased from Sigma (Taufkirchen, Germany) and docetaxel was purchased from a Pharmacy of the University Medical Center Hamburg-Eppendorf (Hamburg, Germany).

### 3.9. Cell Lines and Culture Conditions

The human prostate cancer cells 22Rv1, LNCaP, VCaP and PC-3, DU145 were purchased from ATCC (Manassas, VA, USA). Docetaxel-resistant human prostate cancer cells PC3-DR were generated by the long-term incubation of PC3 cells in the sub-lethal concentrations of docetaxel [30]. The cells were recently authenticated by a commercial service (Multiplexion, Heidelberg, Germany). The cells were cultured as previously described [31].

### 3.10. MTT Assay

Effect of the drugs on viability of the cells was evaluated using MTT assay, as previously described [32]. In brief, 6 × 10^3^ cells per well in 100 μL per well were plated in 96-well plates and treated with the tested compounds at the indicated concentrations. After 48 h of incubation, the MTT reagent was added, and the viability was measured following 2 h of incubation. The vehicle-treated cells were used as a control. IC50s were calculated using GraphPad Prism v.9.1.1 (GraphPad Software, San Diego, CA, USA).

### 3.11. Drug Combination Studies

The PC3-DR cells were treated with the single drugs at the indicated concentrations and their combinations with docetaxel for 48 h in 100 µL/well as described for MTT assay. The viability was measured using MTT assay, and the generated data were further analyzed using SynergyFinder 2.0 software (https://synergyfinder.fimm.fi [33] (accessed on 9 September 2022)) as previously reported [31]. The difference between the expected and observed drug combination effects was evaluated using Zero Interaction Potency (ZIP) reference model [34]. Synergism was identified as areas with positive δ-values (marker with red), whereas antagonism refers to the areas with negative δ-values (marker with green). 

### 3.12. P-Glycoprotein Activity Assay

PC3-DR cells were seeded in a 96-well plate (6 × 10^3^ cells/well in 100 µL/well) in the drug- and docetaxel-free medium, incubated overnight and then treated with the tested drugs for 30 min in PBS. Then calcein-AM solution was added to each well, and the green fluorescence was measured using the plate reader following 15 min of incubation according to the manufacturer’s protocol. The fluorescence was normalized to the background autofluorescence of the drug solutions as well as to the cellular viability, which was evaluated by MTT assay at the same experimental conditions.

### 3.13. Statistical Analysis

The experiments were performed in biological triplicates. Statistical analyses were performed using GraphPad Prism v.9.1.1 (GraphPad Software, San Diego, CA, USA) and the data are represented as mean ± SD (standard deviation). For the analysis of the statistical significance of the difference between the drug-exposed group and the control group, the one-way ANOVA test and Dunnett’s post-hoc test were used; for the difference between two groups, the Student’s t-test was used. The statistically significant difference is indicated as (*) if *p* < 0.05 in either statistical test.

## 4. Conclusions

In conclusion, we report isolation and structure elucidation of five new β-resorcylic acid derivatives—β-resoantarctines A–C (**3**, **5**, **6**), 8-dehydro-β-resoantarctine A (**4**) and 14-hydroxyasperentin B (**1**)—as well as one previously known 14-hydroxyasperentin (**2**) from the alga-derived fungus *Penicillium antarcticum* KMM 4685. For the very first time, an absolute configuration of 14-hydroxyasperentin (**2**) was established using the modified Mosher’s method. The configuration of a new compound **1** was determined based on a comparison of its spectral data with those of **2** as well as using biosynthetical considerations. The new isolated compounds **3**–**6** were structurally and biosynthetically related to resorcylic acid lactones (RAls), but had non-lactonizes structures, possessing an opened macrolide cycle. Compounds **3**, **4** and **5** exhibited a moderate cytotoxic activity in LNCaP, DU145 and 22Rv1 human prostate cancer cells. Remarkably, they inhibited an activity of p-glycoprotein at their noncytotoxic concentrations and consequently synergized with docetaxel in p-glycoprotein-overexpressing drug-resistant cells.

## Figures and Tables

**Table 1:** ^13^C and ^1^H NMR spectroscopic data for compounds **1**–**2**.

No	1		2	
	δ_C_, Type	δ_H_, Mult. (*J* in Hz)	δ_c_, Type	δ_H_, Mult. (*J* in Hz)
1	171.8, C		171.4, C	
2	100.2, C		101.2, C	
3	159.1, C		166.3, C	
4	101.8, CH	6.26, s	102.2, CH	6.19, d (2.2)
5	155.6, C		165.6, C	
6	135.7, C		107.8, C	6.20, brs
7	126.1, C		143.5, C	
8	28.6, CH_2_	3.15, dd (16.7, 3.3) 2.63, dd (16.7, 11.0)	34.4, CH_2_	2.88, dd (16.5, 4.0) 2.85, dd (16.5, 10.7)
9	77.7, CH	4.61, m	77.8, CH	4.65, m
10	40.5, CH_2_	1.92, m 1.85, m	40.3, CH_2_	1.89, m 1.83, m
11	66.4, CH	4.00, m	66.5, CH	3.99, dt (9.0, 3.3)
12	30.0, CH_2_	1.87, m 1.33. m	30.0, CH_2_	1.87, m 1.33. m
13	27.2, CH_2_	1.80, m 1.72, m	27.2, CH_2_	1.79, m 1.73, m
14	69.0, CH	3.70, dt (9.0, 4.3)	69.0, CH	3.70, dt (9.0, 4.3)
15	72.3, CH	3.96, qd (6.6, 4.2)	72.4, CH	3.96, qd (6.6, 4.2)
16	13.1, CH_3_	1.20, d (6.6)	13.1, CH_3_	1.20, d (6.6)

Chemical shifts were measured at 125 MHz and 500 MHz in CD_3_OD.

**Table 2:** ^13^C NMR spectroscopic data for compounds **3**–**6**.

Position	δ_C_, Type			
	3 *	4 *	5 **	6 ***
1	172.4, C	172.3, C	172.5, C	172.6, C
2	106.0, C	105.1, C	106.2, C	105.1, C
3	165.0, C	165.0, C	165.3, C	166.8, C
4	102.6, CH	102.6, CH	101.9, CH	102.4, CH
5	163.0, C	163.6, C	163.5, C	163.2, C
6	111.7, CH	108.9, CH	111.7, CH	113.2, CH
7	149.2, C	144.9, C	149.1, C	145.4, C
8	37.3, CH_2_	132.3, CH	37.2, CH_2_	39.9, CH_2_
9	33.1, CH_2_	133.6, CH	32.8, CH_2_	74.2, CH
10	30.8, CH_2_	34.0, CH_2_	30.2, CH_2_	27.2, CH_2_
11	26.8, CH_2_	26.6, CH_2_	24.8, CH_2_	18.7, CH_2_
12	40.1, CH_2_	39.7, CH_2_	44.3, CH_2_	33.3, CH_2_
13	68.6, CH	68.4, CH	209.0, C	67.8, CH
14	23.5, CH_3_	23.5, CH_3_	29.8, CH_3_	21.3, CH_3_
1′	67.2, CH_2_	67.1, CH_2_	68.1, CH_2_	70.0, CH_2_
2′	71.1, CH	71.0, CH	71.3, CH	70.8, CH
3′	64.5, CH_2_	64.3, CH_2_	73.8, CH	73.1, CH
4′			64.6, CH_2_	64.8, CH_2_

* Chemical shifts were measured at 75 MHz in CD_3_OD. ** Chemical shifts were measured at 125 MHz in CD_3_OD. *** Chemical shifts were measured at 175 MHz in acetone-d_6_.

**Table 3:** ^1^H NMR spectroscopic data for compounds **3**–**6**.

Position	δ_H_, Mult. (*J* in Hz)			
	3 *	4 *	5 **	6 ***
4	6.15, d (2.5)	6.20, d (2.4)	6.16, d (2.5)	6.28, brs
6	6.20, d (2.5)	6.38, d (2.4)	6.20, d (2.5)	6.28, brs
8	2.88, m 2.84, m	6.98, dt (15.5, 1.5)	2.90, m 2.84, m	3.45, dd (12.5, 3.9) 3.14, dd (12.4, 9.1)
9	1.58, m 1.55, m	5.91, dt (15.5, 7.0)	1.57, m (2H)	4.03, m
10	1.38, m (2H)	2.24, m (2H)	1.36, m (2H)	1.54, m 1.51, m
11	1.39, m (2H)	1.60, m 1.52, m	1.57, m (2H)	1.85, m 1.65, m
12	1.41, m (2H)	1.51, m 1.45, m	2.46, m (2H)	1.66, m 1.24, m
13	3.70, ddd (12.0, 9.2, 5.6)	3.75, dd (11.9, 6.0)		3.91, m
14	1.13, d (6.0)	1.16, d (6.2)	2.11, s	1.13, d (6.3)
1′	4.41, ddd (11.5, 4.3, 1.3) 4.30, ddd (11.5, 6.6, 1.3)	4.39, dd (11.4, 4.5) 4.31, dd (11.4, 5.9)	4.57, dd (11.5, 4.3, 1.3) 4.38, dd (11.5, 6.6, 1.3)	4.62, dd (11.2, 2.1) 4.33, dd (11.2, 7.8)
2′	3.96, m	3.95, m	3.88, td (7.0, 2,6)	3.99, m
3′	3.62, ddd (11.4, 5.5, 0.6) 3.60, ddd (11.4, 5.5, 1.0)	3.64, dd (11.2, 5.5) 3.60, dd (11.2, 5.5)	3.61, m	3.62, ddd (8.0, 5.6, 4.0)
4′			3.77, dd (10.6, 3.1) 3.64, m	3.80, dd (10.8, 4.0) 3.66, dd (10.8, 5.7)
C-3-OH				11.7, s
C-5-OH				9.17, s
C-2′-OH				5.00 d (5.3)
C-3′-OH				3.99 d (5.8)
C-4′-OH				3.62 d (5.4)

* Chemical shifts were measured at 500 MHz in CD_3_OD. ** Chemical shifts were measured at 500 MHz in CD_3_OD. *** Chemical shifts were measured at 700 MHz in acetone-d_6_.

**Table 4:** Cytotoxic activity of the isolated new compounds **3**–**5** in six human prostate cancer cell lines determined using MTT assay following 48 h of treatment. Docetaxel was used as a positive control. The IC_50_ values were calculated using GraphPad Prism v.9.1.1 software and are represented as mean ± SD.

Cell Line	Compound, IC_50_ [µM]			Control, IC_50_ [nM]
	3	4	5	Doce
PC3	>100	>100	>100	7.5 ± 7.1
PC3-DR	21.4 ± 18.8	>100	>100	325 ± 22
LNCaP	31 ± 2	44.1 ± 3.8	79.2 ± 2.8	5.1 ± 0.6
DU145	40.4 ± 2.7	50.4 ± 3.8	82.5 ± 4.1	2.2 ± 0.6
22Rv1	41.9 ± 2.1	51.3 ± 2.8	55.4 ± 3.1	0.6 ± 0.1
VCaP	>100	>100	>100	0.23 ± 0.22

## Data Availability

The original data are available from the correspondent author on request.

## Supplementary Materials

The following supporting information can be downloaded at: https://www.mdpi.com/article/10.3390/md21030178/s1, Figure S1: HRESIMS for 1; Figure S2: 1H NMR spectrum of 1 measured at 500 MHz in MeOD; Figure S3: 13C NMR spectrum of 1 measured at 125 MHz in MeOD; Figure S4: DEPT-135 spectrum of 1 measured at 125 MHz in MeOD; Figure S5: HSQC spectrum of 1 measured in MeOD; Figure S6: COSY spectrum of 1 measured in MeOD; Figure S7: HMBC spectrum of 1 measured in MeOD; Figure S8: HRESIMS for 2; Figure S9: 1H NMR spectrum of 2 measured at 500 MHz in MeOD; Figure S10: 13C NMR spectrum of 2 measured at 125 MHz in MeOD; Figure S11: DEPT-135 spectrum of 2 measured at 125 MHz in MeOD; Figure S12: HSQC spectrum of 2 measured in MeOD; Figure S13: COSY spectrum of 2 measured in MeOD; Figure S14: HMBC spectrum of 2 measured in DMSO-d6; Figure S15: HRESIMS for 3; Figure S16: 1H NMR spectrum of 3 measured at 500 MHz in MeOD; Figure S17: 13C NMR spectrum of 3 measured at 75 MHz in MeOD; Figure S18: DEPT-135 spectrum of 3 measured at 75 MHz in MeOD; Figure S19: HSQC spectrum of 3 measured in MeOD; Figure S20: COSY spectrum of 3 measured in MeOD; Figure S21: HMBC spectrum of 3 measured in MeOD; Figure S22: HRESIMS for 4; Figure S23: 1H NMR spectrum of 4 measured at 500 MHz in MeOD; Figure S24: 13C NMR spectrum of 4 measured at 75 MHz in MeOD; Figure S25: DEPT-135 spectrum of 4 measured at 75 MHz in MeOD; Figure S26: HSQC spectrum of 4 measured in MeOD; Figure S27: COSY spectrum of 4 measured in MeOD; Figure S28: HMBC spectrum of 4 in MeOD; Figure S29: HRESIMS for 5; Figure S30: 1H NMR spectrum of 5 measured at 500 MHz in MeOD; Figure S31: 13C NMR spectrum of 5 measured at 125 MHz in MeOD; Figure S32: DEPT-135 spectrum of 5 measured at 125 MHz in MeOD; Figure S33: HSQC spectrum of 5 measured in MeOD; Figure S34: COSY spectrum of 5 measured in MeOD; Figure S35: HMBC spectrum of 5 in MeOD; Figure S36: HRESIMS for 6; Figure S37: 1H NMR spectrum of 6 measured at 700 MHz in acetone-d6; Figure S38: 13C NMR spectrum of 6 measured at 175 MHz in acetone-d6; Figure S39: DEPT-135 spectrum of 6 measured at 175 MHz in acetone-d6; Figure S40: HSQC spectrum of 6 measured in acetone-d6; Figure S41: COSY spectrum of 6 measured in acetone-d6; Figure S42: HMBC spectrum of 6 in acetone-d6; Figure S43: HRESIMS data for 6a; Figure S44: 1H NMR spectrum of 6a measured at 700 MHz in acetone-d6; Figure S45: 13C NMR spectrum of 6a measured at 175 MHz in acetone-d6; Figure S46: HSQC spectrum of 6a measured in acetone-d6; Figure S47: COSY spectrum of 6a measured in acetone-d6; Figure S48: UV and CD data for 1; Figure S49: UV and CD data for 3; Figure S50: UV and CD data for 4; Figure S51: UV and CD data for 5; Figure S52: UV and CD data for 6; Figure S53: 1H NMR spectrum for MTPA-2a (S); Figure S54: 1H NMR spectrum for MTPA-2b (R); Figure S55: COSY spectrum for MTPA-2b (R); Figure S56: HSQC spectrum for MTPA-2b (R); Figure S57: Scheme of isolation compounds **1**–**4**; Figure S58: ROESY spectrum for 1 in MeOD; Figure S59: ROESY spectrum for 2 in MeOD; Figure S60: ROESY spectrum for 3 in MeOD; Figure S61: ROESY spectrum for 4 in MeOD; Figure S62: ROESY spectrum for 5 in MeOD; Figure S63: ROESY spectrum for 6 in acetone-d6.
