# Supplementary material for: New Bioactive β-Resorcylic Acid Derivatives from the Alga-Derived Fungus Penicillium antarcticum KMM 4685
Source: Mar Drugs. 2023 Mar 14;21(3):178. doi: 10.3390/md21030178 (PMC10058225; doi:10.3390/md21030178)
Supplement: Supplementary file 1 [file marinedrugs-21-00178-s001.zip › marinedrugs-2273291-supplementary.pdf]

Supplementary Materials

New Bioactive β-Resorcylic Acid Derivatives from the Alga-Derived Fungus *Penicillium antarcticum* KMM 4685

Elena V. Leshchenko [1,2,*], Alexandr S. Antonov [1], Gleb V. Borkunov [1,2], Jessica Hauschild [3,4], Olesya I. Zhuravleva [1,2], Yuliya V. Khudyakova [1], Alexander S. Menshov [1], Roman S. Popov [1], Natalya Yu. Kim [1], Markus Graefen [4], Carsten Bokemeyer [3], Gunhild von Amsberg [3,4], Anton N. Yurchenko [1] and Sergey A. Dyshlovoy [3,4,*]

[1] G.B. Elyakov Pacific Institute of Bioorganic Chemistry, Far Eastern Branch of the Russian Academy of Sciences, 159 Prospect 100-letiya Vladivostoka, Vladivostok 690022, Russia

[2] Institute of High Technologies and Advanced Materials, Far Eastern Federal University, Vladivostok 690922, Russia

[3] Department of Oncology, Hematology and Bone Marrow Transplantation with Section Pneumology, Hubertus Wald Tumorzentrum – University Cancer Center Hamburg (UCCH), University Medical Center Hamburg-Eppendorf, 20251 Hamburg, Germany

[4] Martini-Klinik, Prostate Cancer Center, University Hospital Hamburg-Eppendorf, 20251 Hamburg, Germany

* Correspondence: leshchenko.ev@dvfu.ru (E.V.L.); s.dyshlovoy@uke.de (S.A.D.).

Figure S1. HRESIMS for 1

Figure S2. ^{1}H NMR spectrum of 1 measured at 500 MHz in MeOD

Figure S3. ^{13}C NMR spectrum of 1 measured at 125 MHz in MeOD

Figure S4. DEPT-135 spectrum of 1 measured at 125 MHz in MeOD

Figure S5. HSQC spectrum of 1 measured in MeOD

Figure S6. COSY spectrum of 1 measured in MeOD

Figure S7. HMBC spectrum of 1 measured in MeOD

Figure S8. HRESIMS for 2

Figure S9. ^{1}H NMR spectrum of 2 measured at 500 MHz in MeOD

Figure S10. ^{13}C **NMR spectrum of 2 measured at 125 MHz in MeOD**

Figure S11. DEPT-135 spectrum of 2 measured at 125 MHz in MeOD

Figure S12. HSQC spectrum of 2 measured in MeOD

Figure S13. COSY spectrum of 2 measured in MeOD

Figure S14. HMBC spectrum of 2 measured in DMSO-d6

Figure S15. HRESIMS for 3

Figure S16. ^{1}H **NMR spectrum of 3 measured at 500 MHz in MeOD**

Figure S17. ^{13}C **NMR spectrum of 3 measured at 75 MHz in MeOD**

Figure S18. DEPT-135 spectrum of 3 measured at 75 MHz in MeOD

Figure S19. HSQC spectrum of 3 measured in MeOD

Figure S20. COSY spectrum of 3 measured in MeOD

Figure S21. HMBC spectrum of 3 measured in MeOD

Figure S22. HRESIMS for 4

Figure S23. ^{1}H **NMR spectrum of 4 measured at 500 MHz in MeOD**

Figure S24. ^{13}C **NMR spectrum of 4 measured at 75 MHz in MeOD**

Figure S25. DEPT-135 spectrum of 4 measured at 75 MHz in MeOD

Figure S26. HSQC spectrum of 4 measured in MeOD

Figure S27. COSY spectrum of 4 measured in MeOD

Figure S28. HMBC spectrum of 4 in MeOD

Figure S29. HRESIMS for 5

Figure S30. ^{1}H **NMR spectrum of 5 measured at 500 MHz in MeOD**

Figure S31. ^{13}C NMR spectrum of 5 measured at 125 MHz in MeOD

Figure S32. DEPT-135 spectrum of 5 measured at 125 MHz in MeOD

Figure S33. HSQC spectrum of 5 measured in MeOD

Figure S34. COSY spectrum of 5 measured in MeOD

Figure S35. HMBC spectrum of 5 in MeOD

Figure S36. HRESIMS for 6

Figure S37. ^{1}H NMR spectrum of 6 measured at 700 MHz in acetone-d6

Figure S38. ^{13}C NMR spectrum of 6 measured at 175 MHz in acetone-d6

Figure S39. DEPT-135 spectrum of 6 measured at 175 MHz in acetone-d6

Figure S40. HSQC spectrum of 6 measured in acetone-d6

Figure S41. COSY spectrum of 6 measured in acetone-d6

Figure S42. HMBC spectrum of 6 in acetone-d6

Figure S43. HRESIMS data for 6a

Figure S44. ^{1}H NMR spectrum of 6a measured at 700 MHz in acetone-d6

Figure S45. ^{13}C NMR spectrum of 6a measured at 175 MHz in acetone-d6

Figure S46. HSQC spectrum of 6a measured in acetone-d6

Figure S47. COSY spectrum of 6a measured in acetone-d6

Figure S48. UV and CD data for 1

Figure S49. UV and CD data for 3

Figure S50. UV and CD data for 4

Figure S51. UV and CD data for 5

Figure S52. UV and CD data for 6

Figure S53. 1H NMR spectrum for MTPA-2a (S)

Figure S54. 1H NMR spectrum for MTPA-2b (R)

Figure S55. COSY spectrum for MTPA-2b (R)

Figure S56. HSQC spectrum for MTPA-2b (R)

Figure S57. Scheme of isolation compounds 1-4.

Figure S58. ROESY spectrum for 1 in MeOD

Figure S59. ROESY spectrum for 2 in MeOD

Figure S60. ROESY spectrum for 3 in MeOD

Figure S61. ROESY spectrum for 4 in MeOD

Figure S62. ROESY spectrum for 5 in MeOD

Figure S63. ROESY spectrum for 6 in acetone-d6

Figure S1. HRESIMS for 1

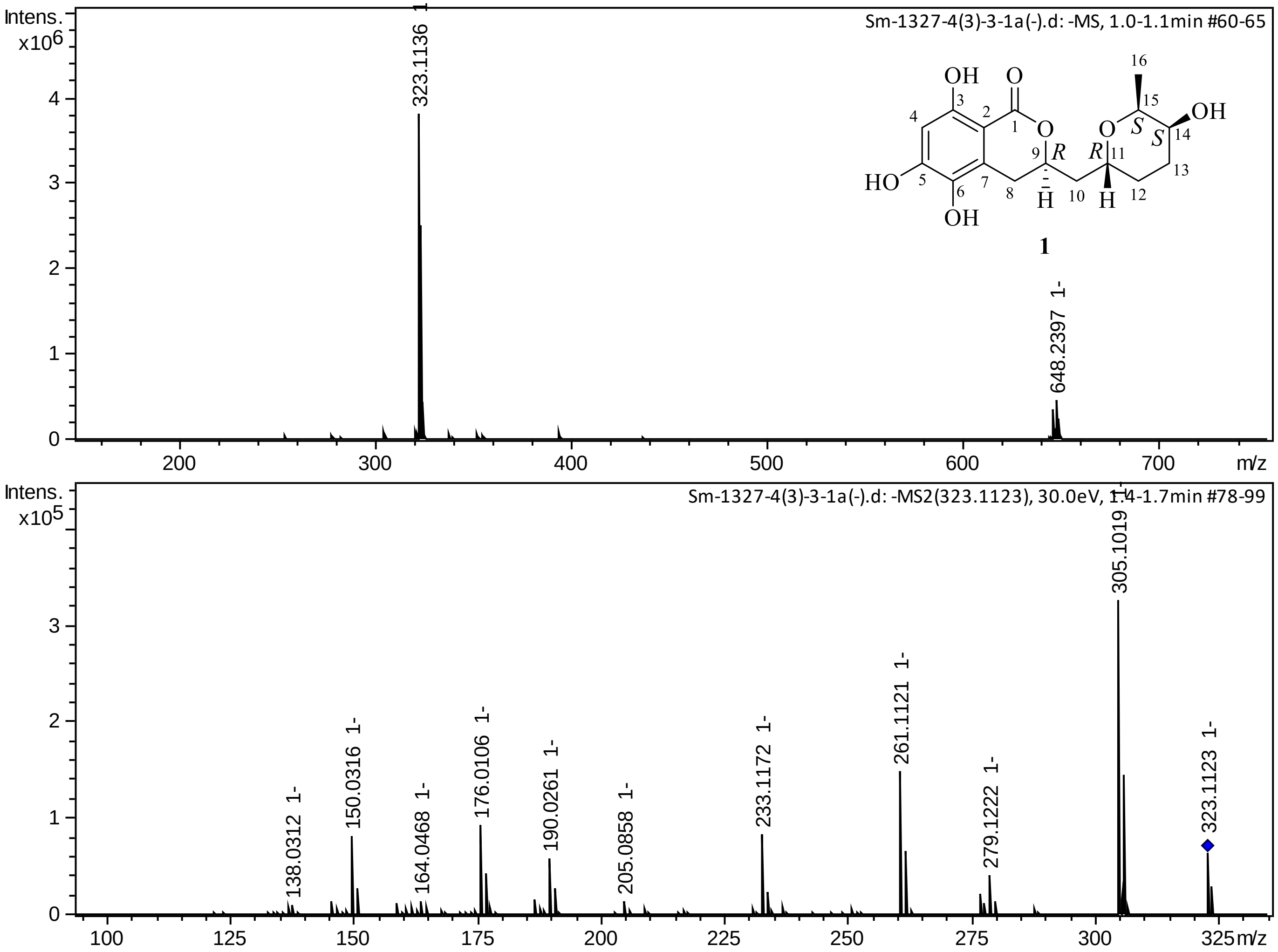

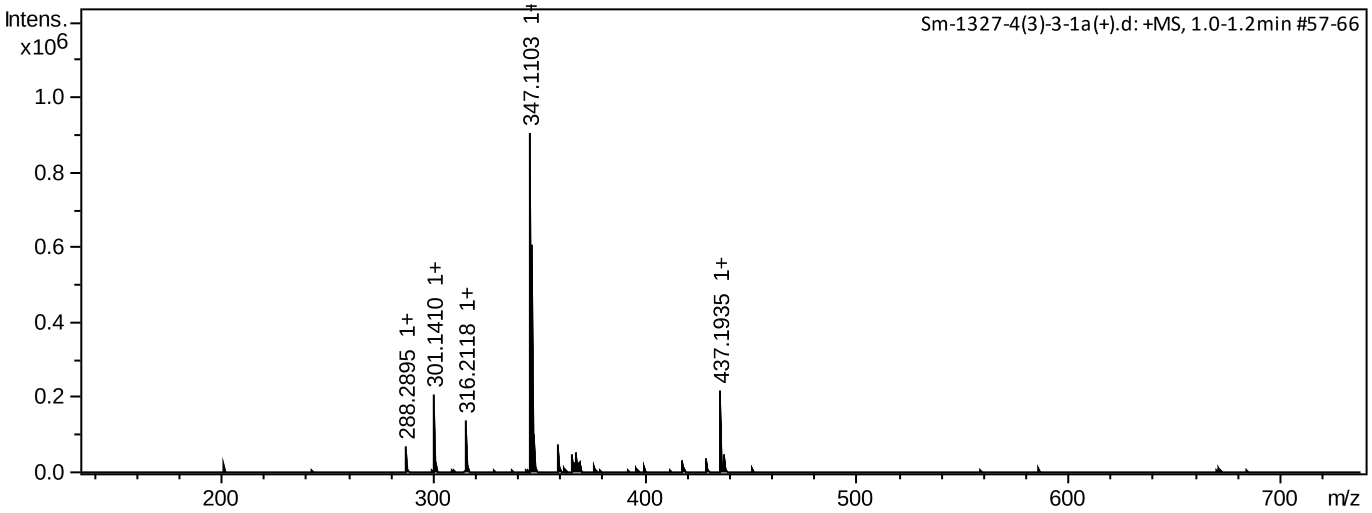

	meas	calc	Δ (ppm)
$[M-H]^-$	323,1136	323,1136	0,2
$[M+Na]^+$	347,1103	347,1101	-0,4

Figure S2. ¹H NMR spectrum of **1** measured at 500 MHz in MeOD

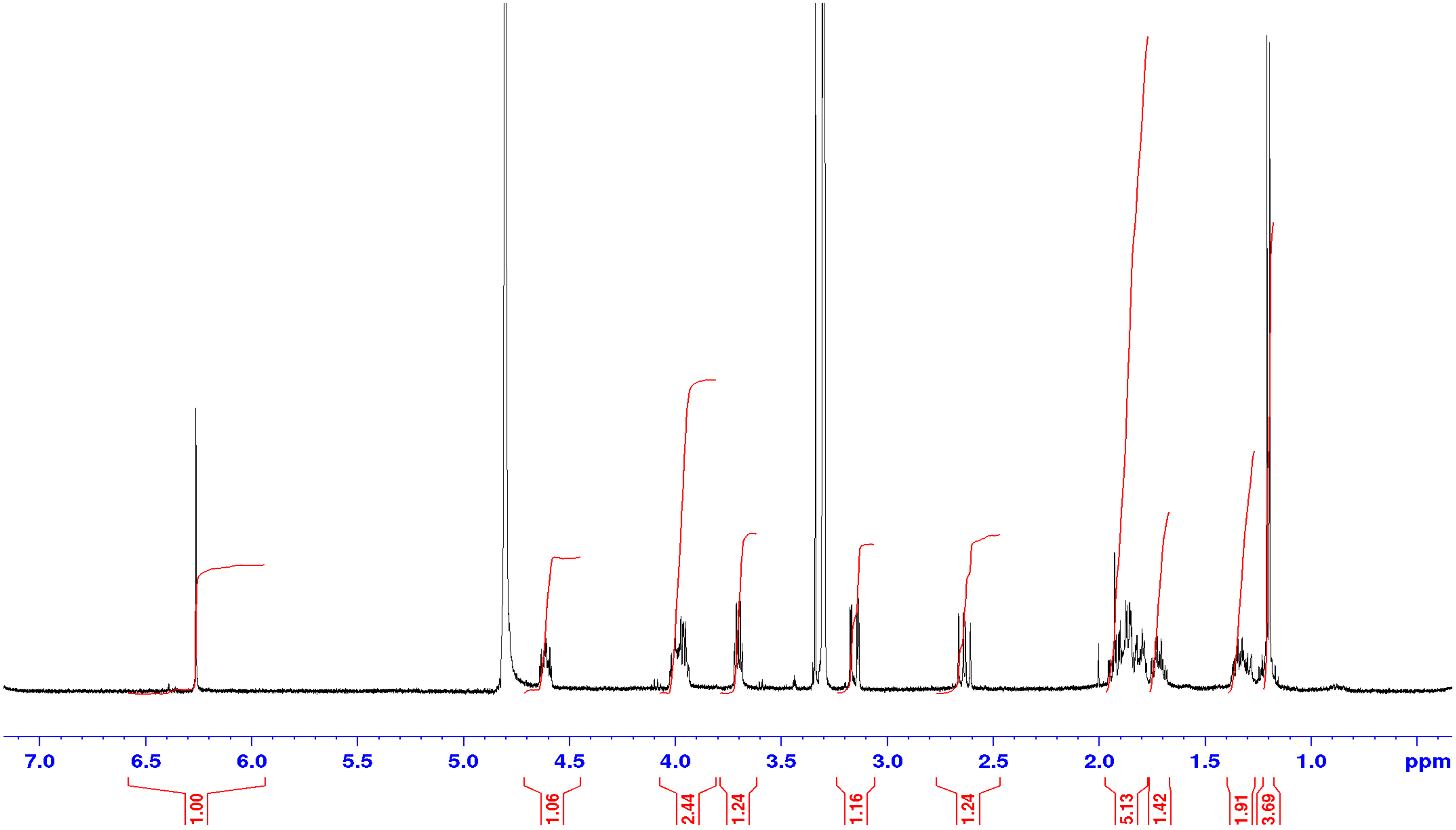

Figure S3. ^{13}C **NMR spectrum of 1 measured at 125 MHz in MeOD**

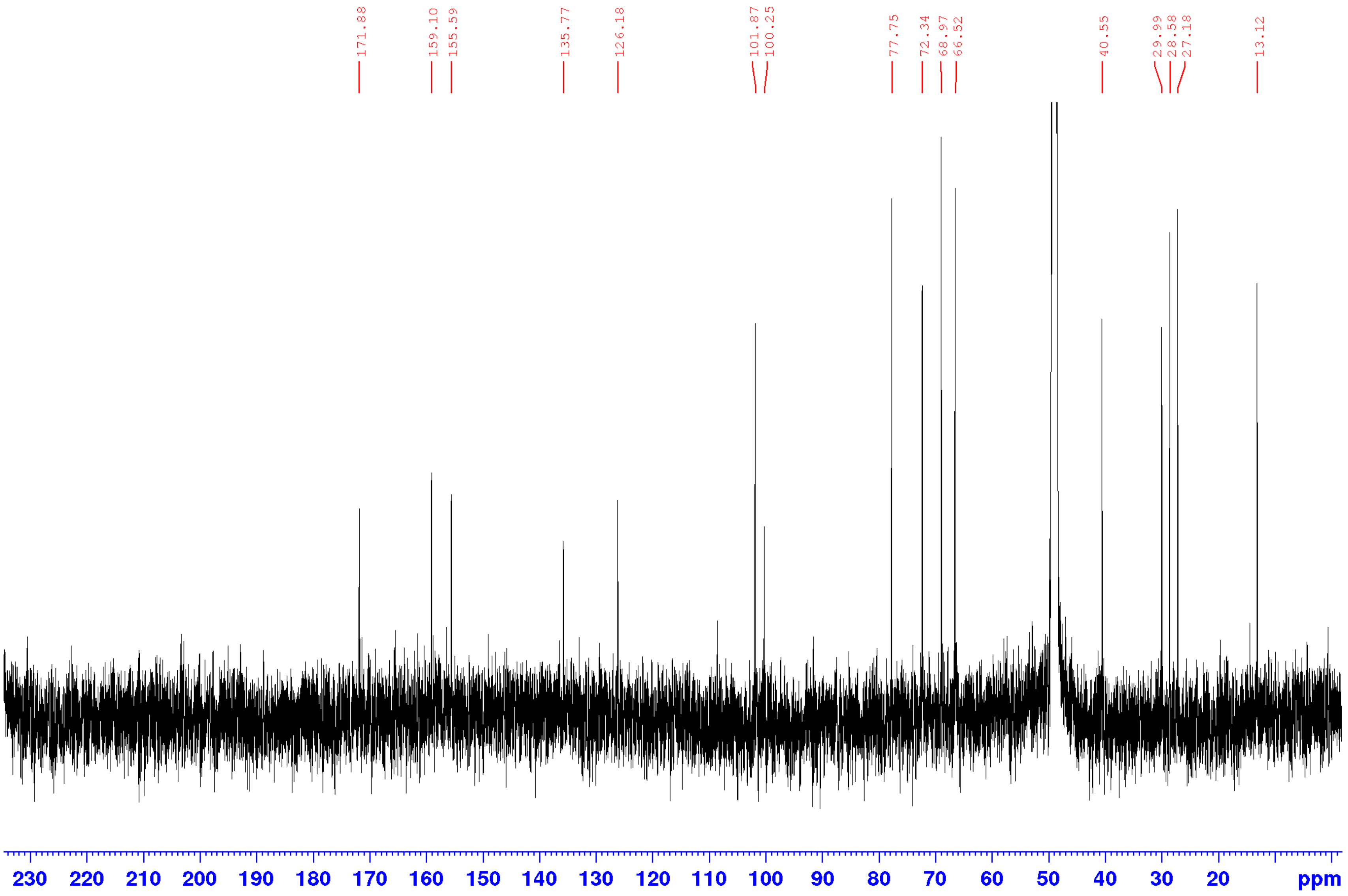

Figure S4. DEPT-135 spectrum of 1 measured at 125 MHz in MeOD

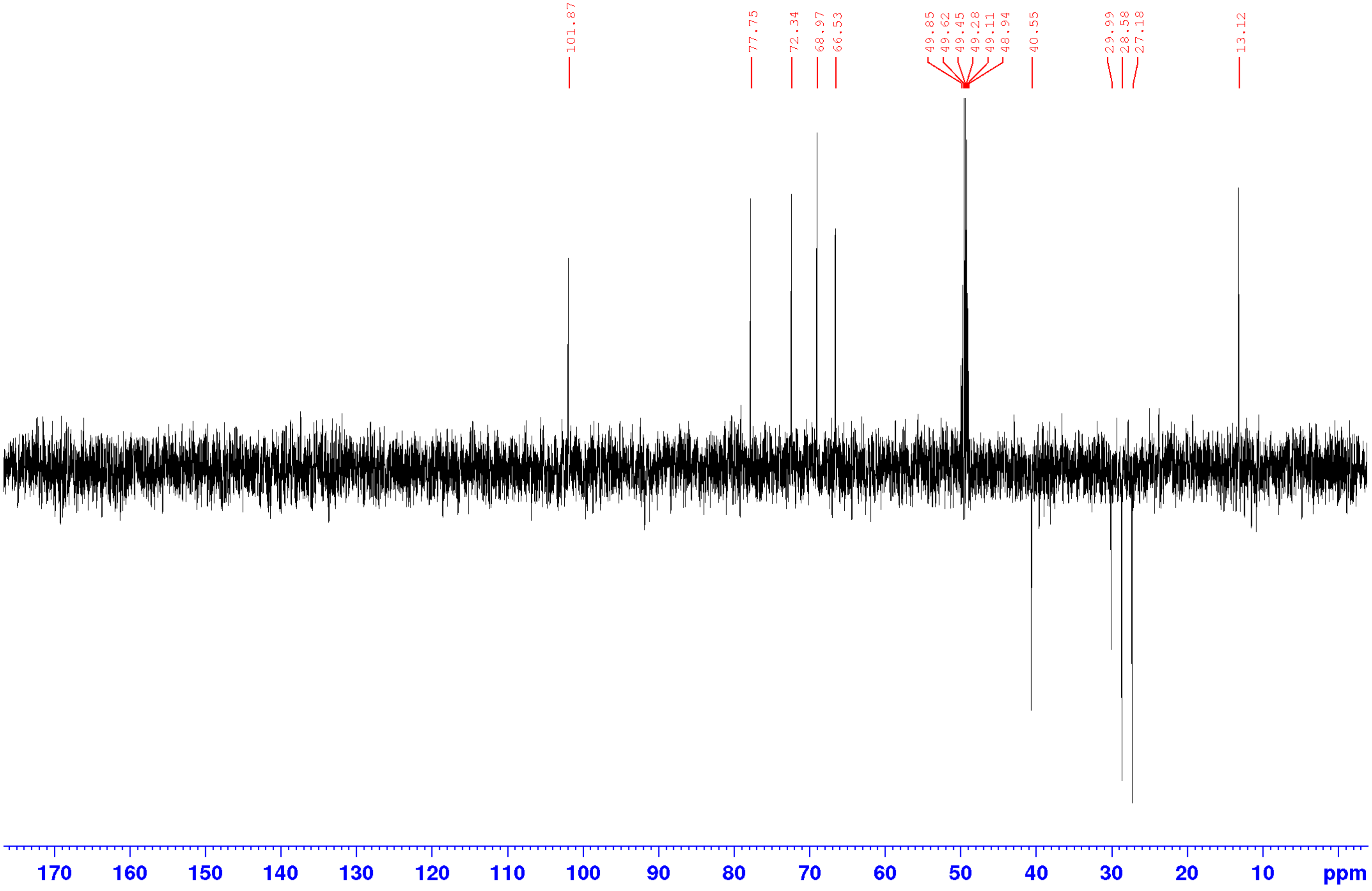

Figure S5. HSQC spectrum of 1 measured in MeOD

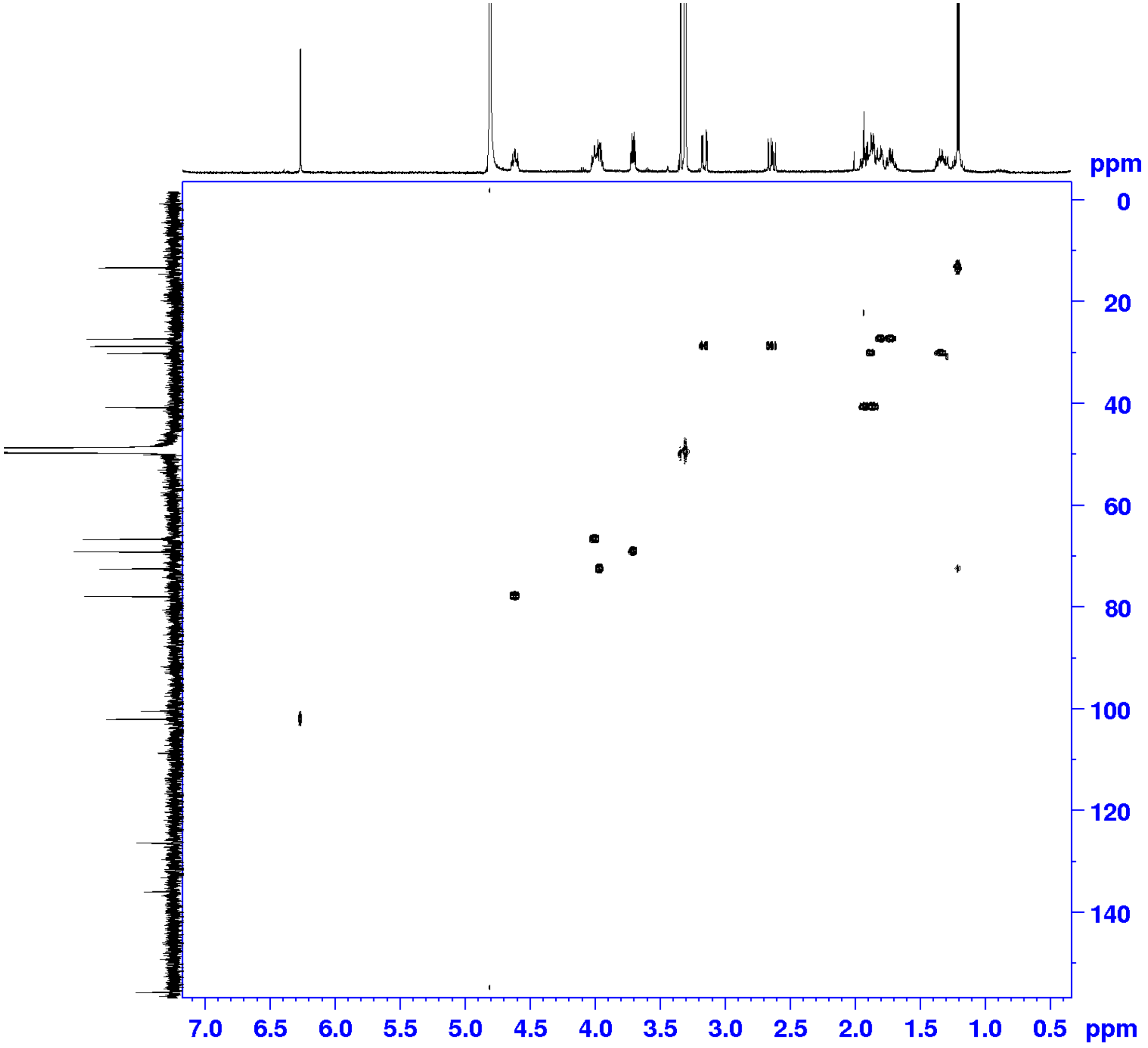

Figure S6. COSY spectrum of 1 measured in MeOD

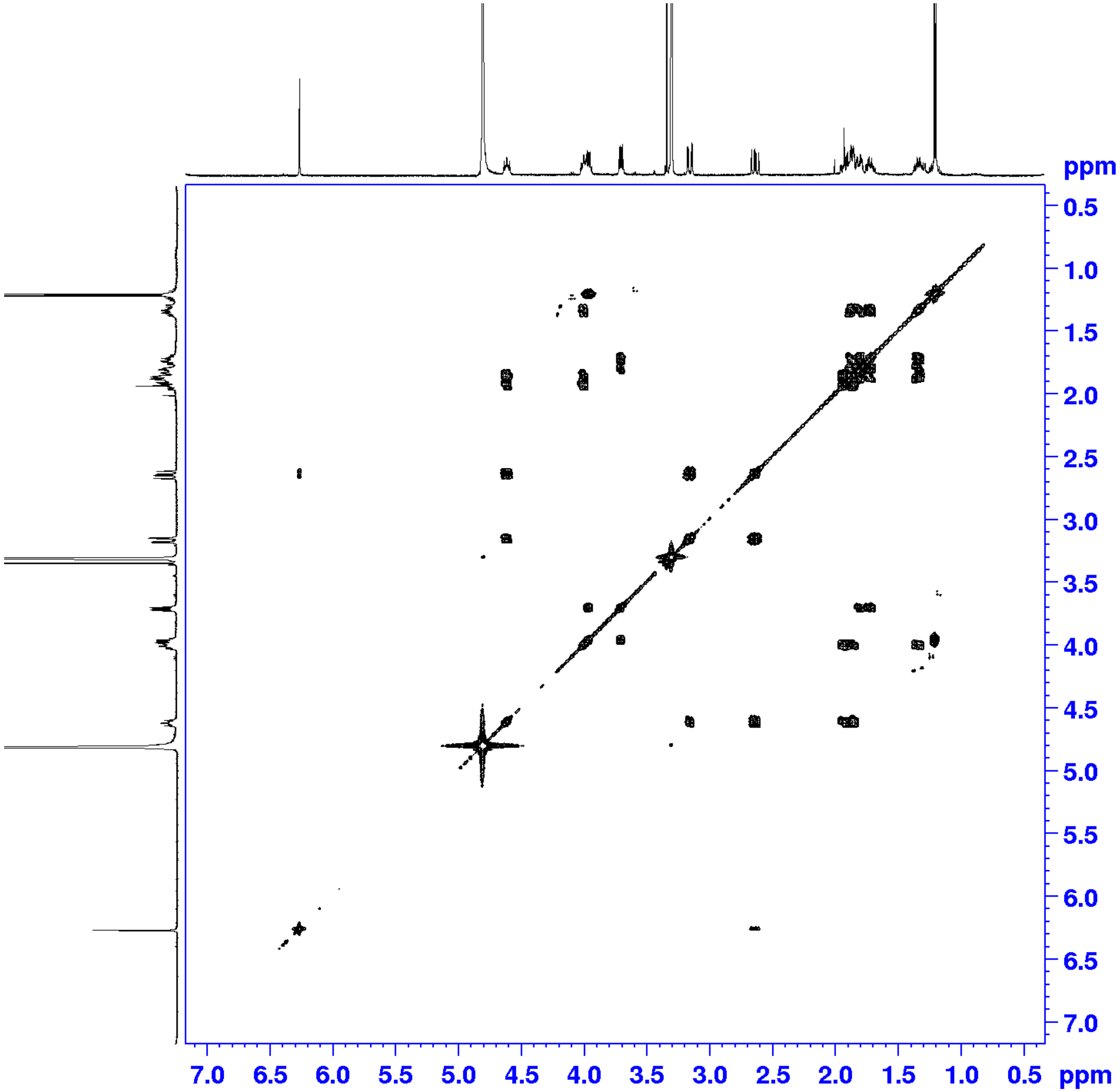

Figure S7. HMBC spectrum of 1 measured in MeOD

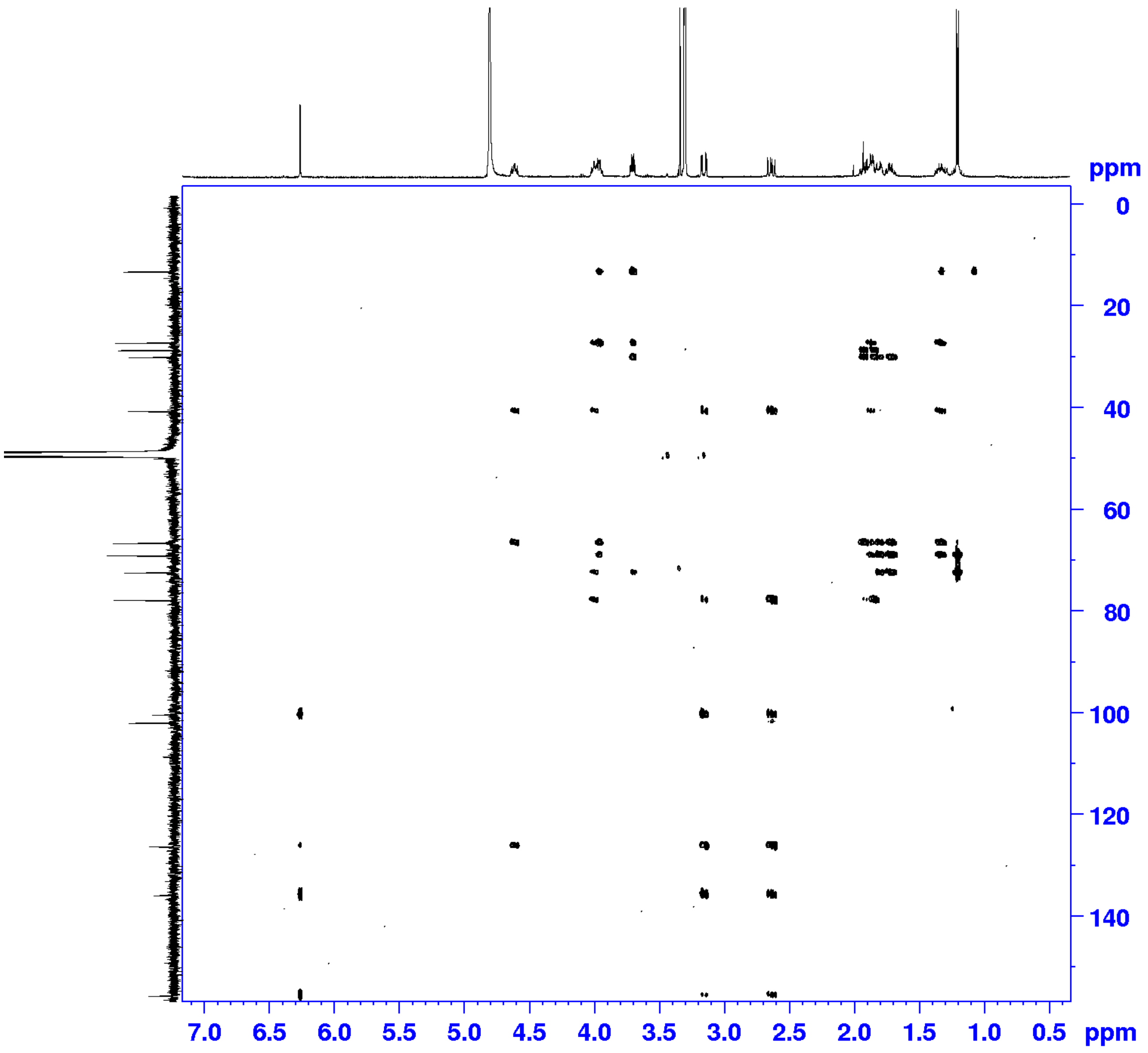

Figure S8. HRESIMS for 2

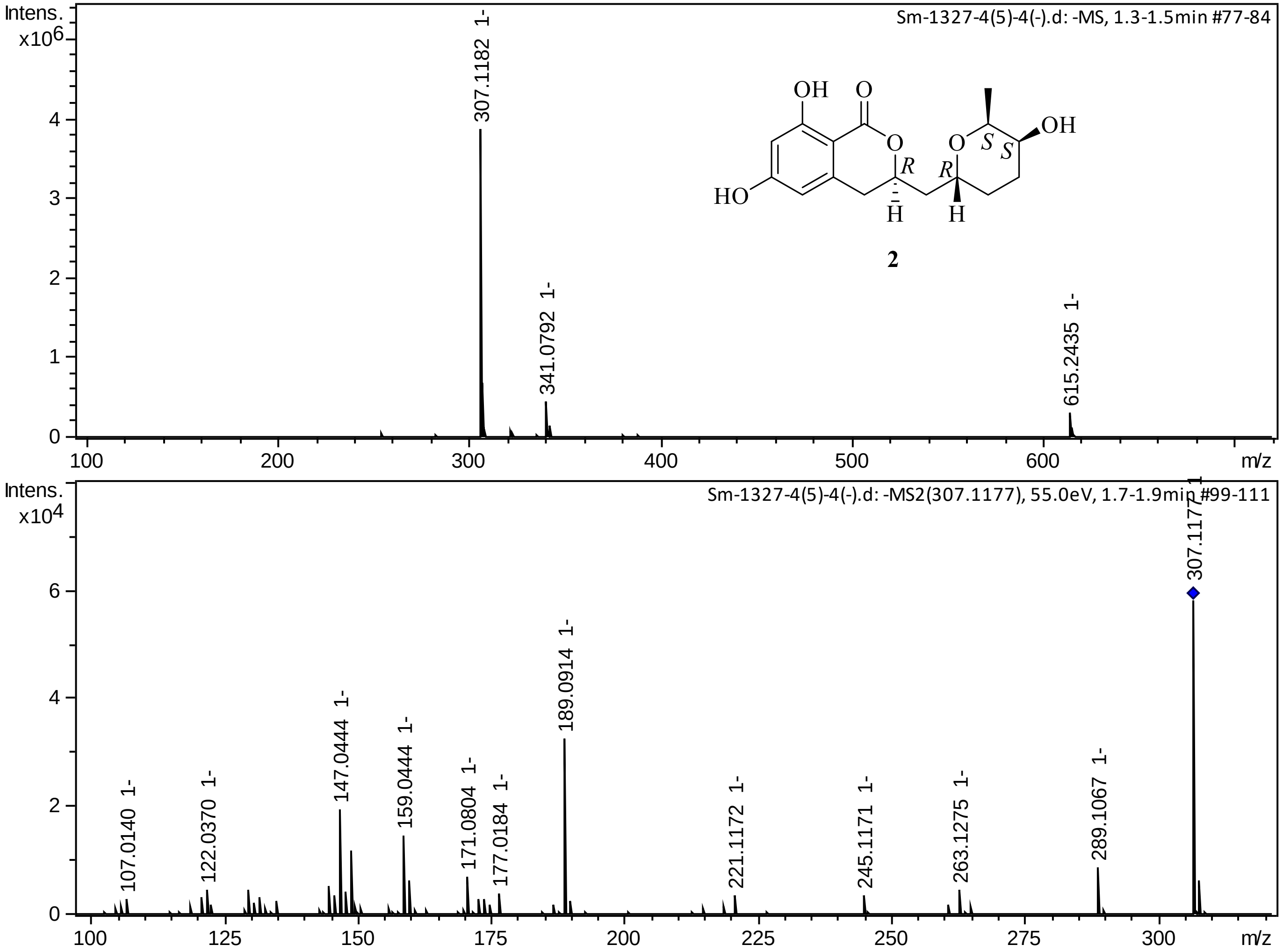

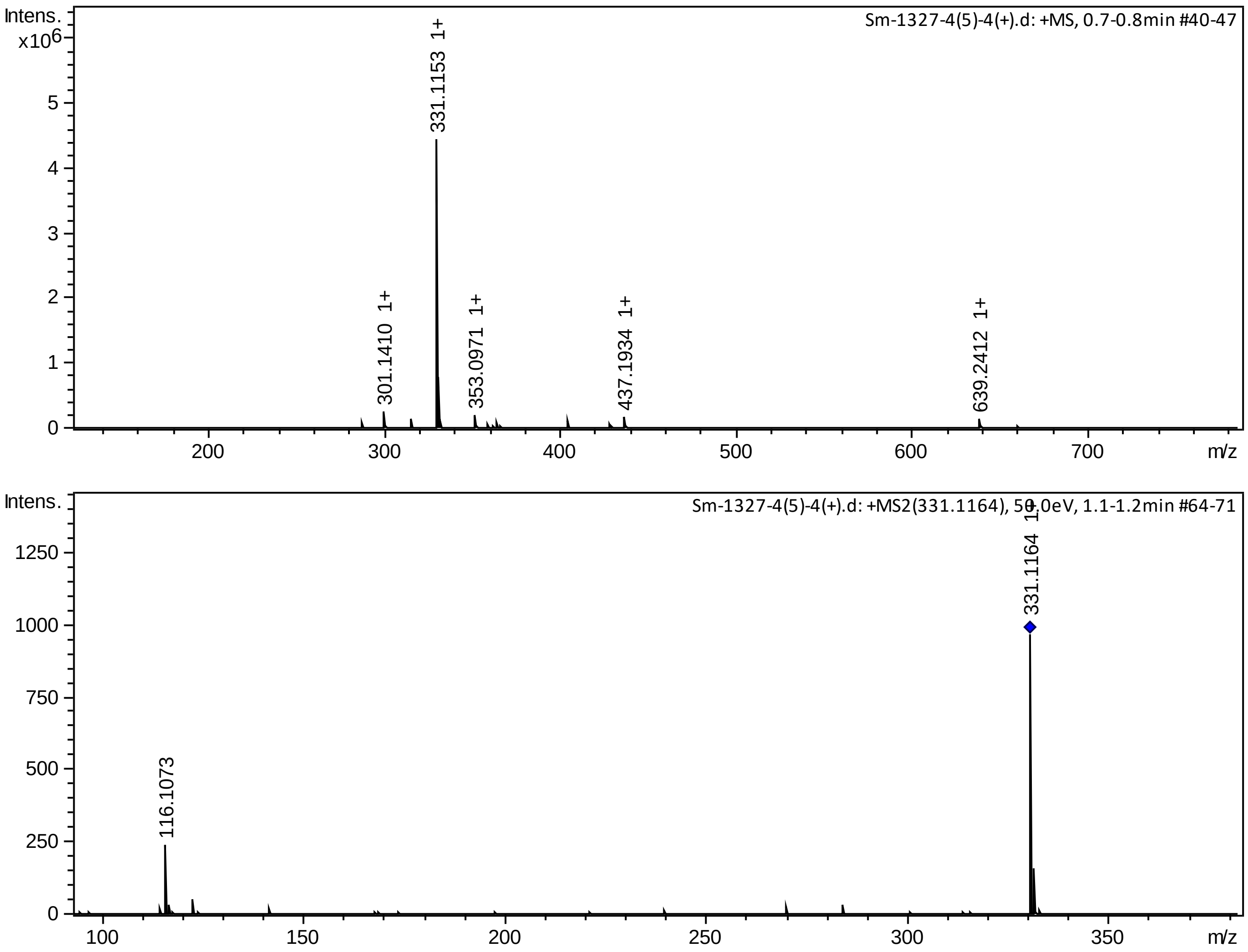
Intens.
x10^6
Sm-1327-4(5)-4(+).d: +MS, 0.7-0.8min #40-47
331.1153 1+
301.1410 1+
353.0971 1+
437.1934 1+
639.2412 1+
m/z
Intens.
Sm-1327-4(5)-4(+).d: +MS2(331.1164), 50.0eV, 1.1-1.2min #64-71
331.1164 1+
116.1073
m/z

Figure S9. ^{1}H NMR spectrum of 2 measured at 500 MHz in MeOD

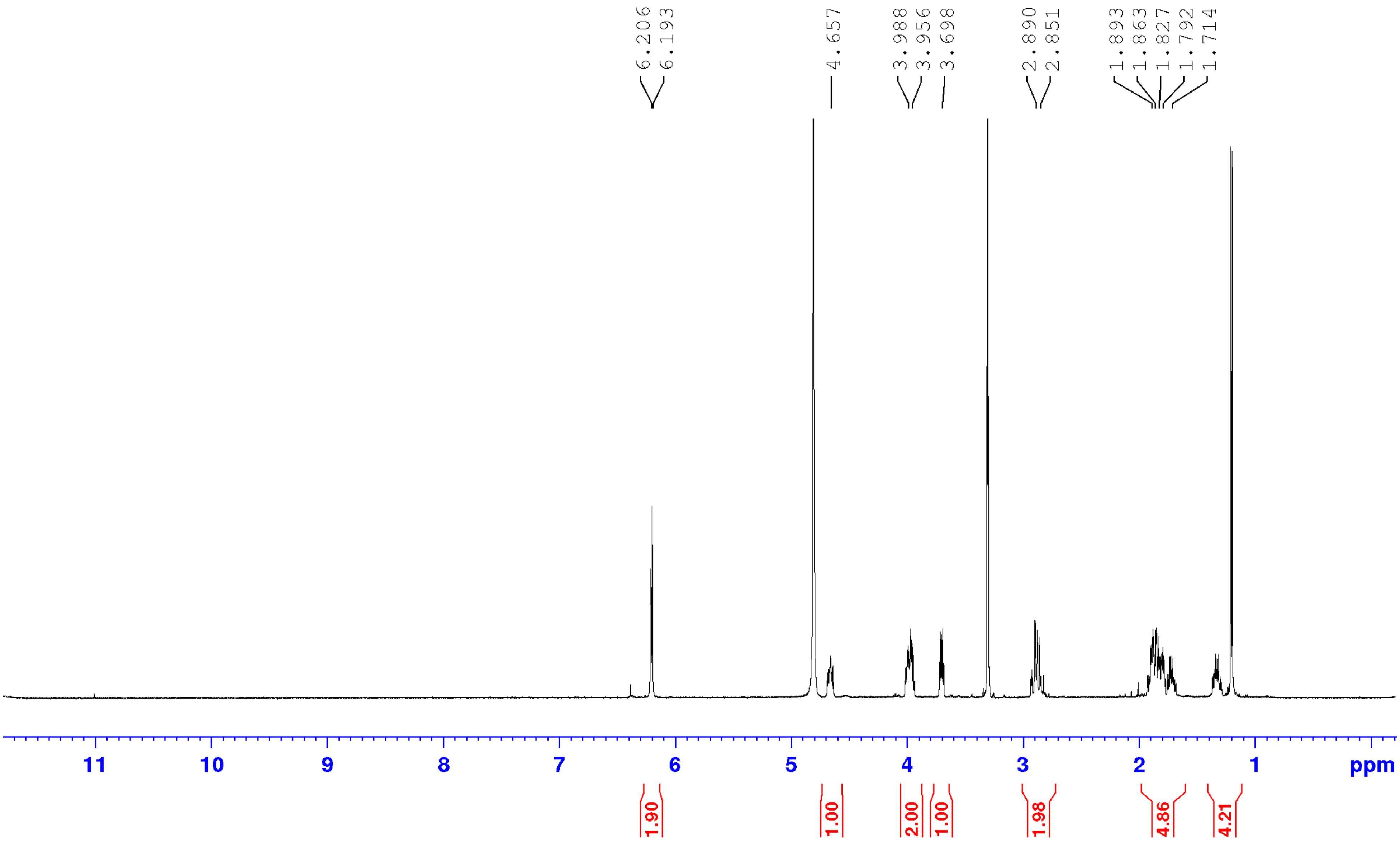

Figure S10. ^{13}C NMR spectrum of **2** measured at 125 MHz in MeOD

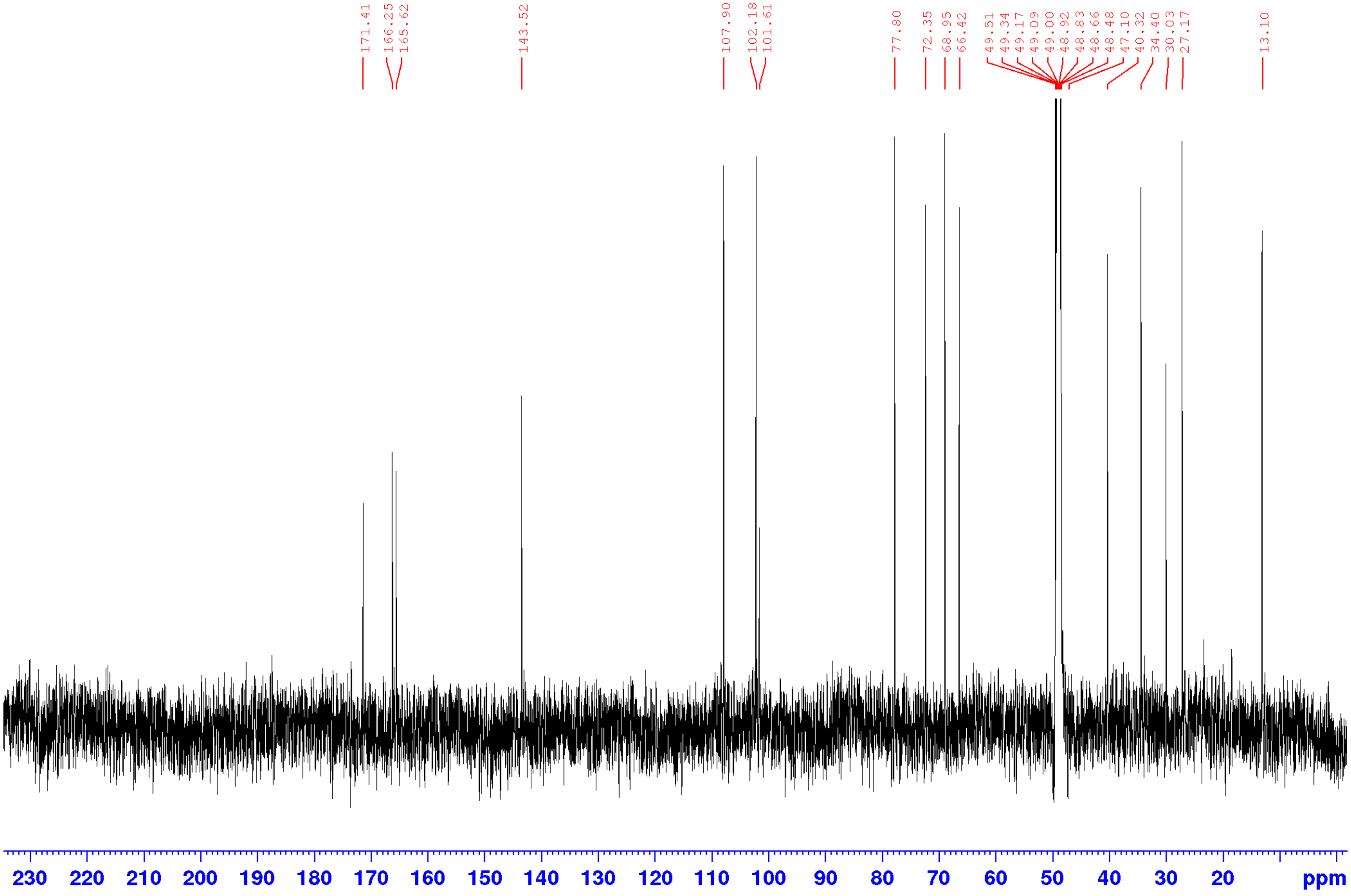

Figure S11. DEPT-135 spectrum of 2 measured at 125 MHz in MeOD

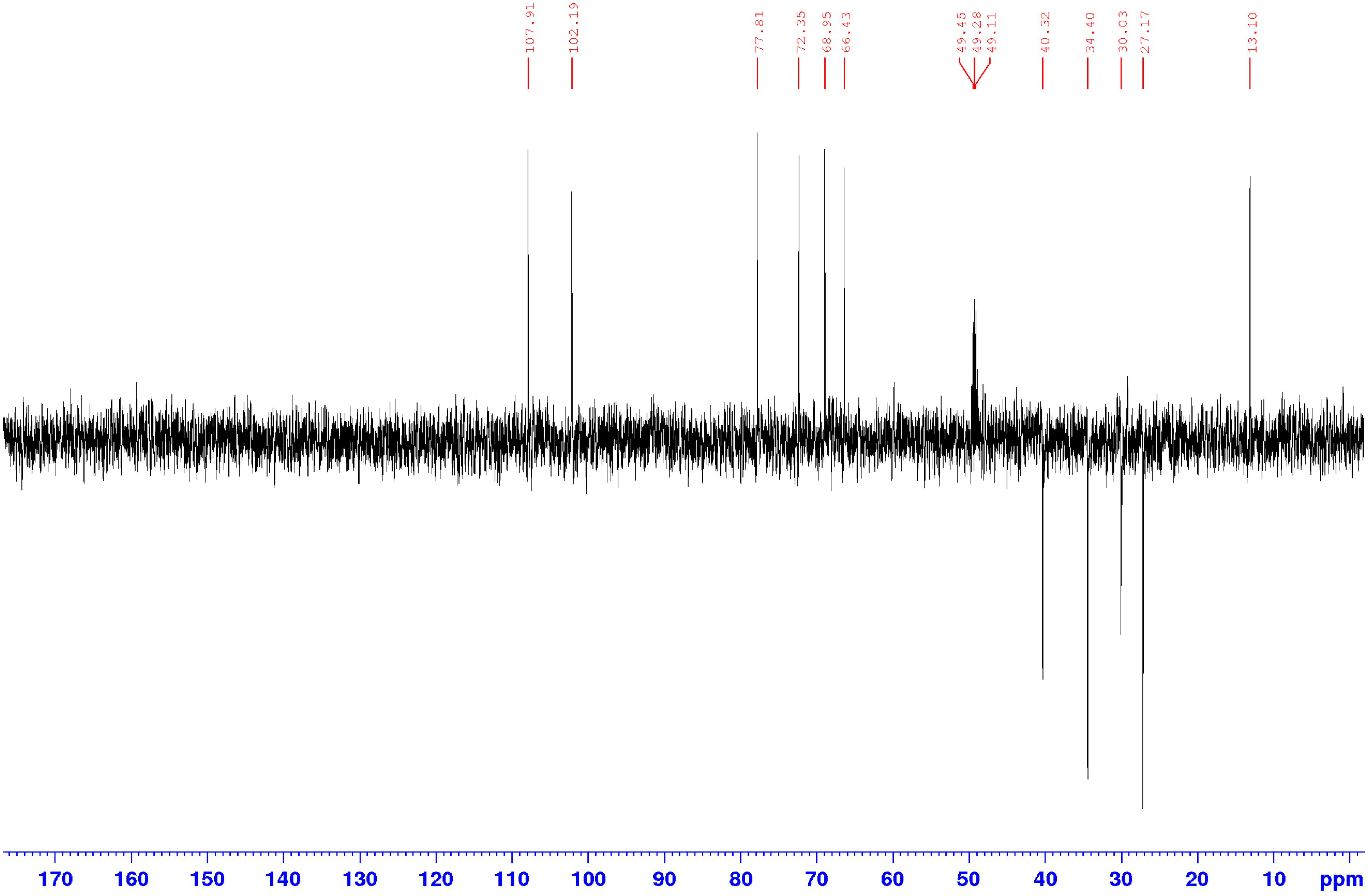

Figure S12. HSQC spectrum of 2 measured in MeOD

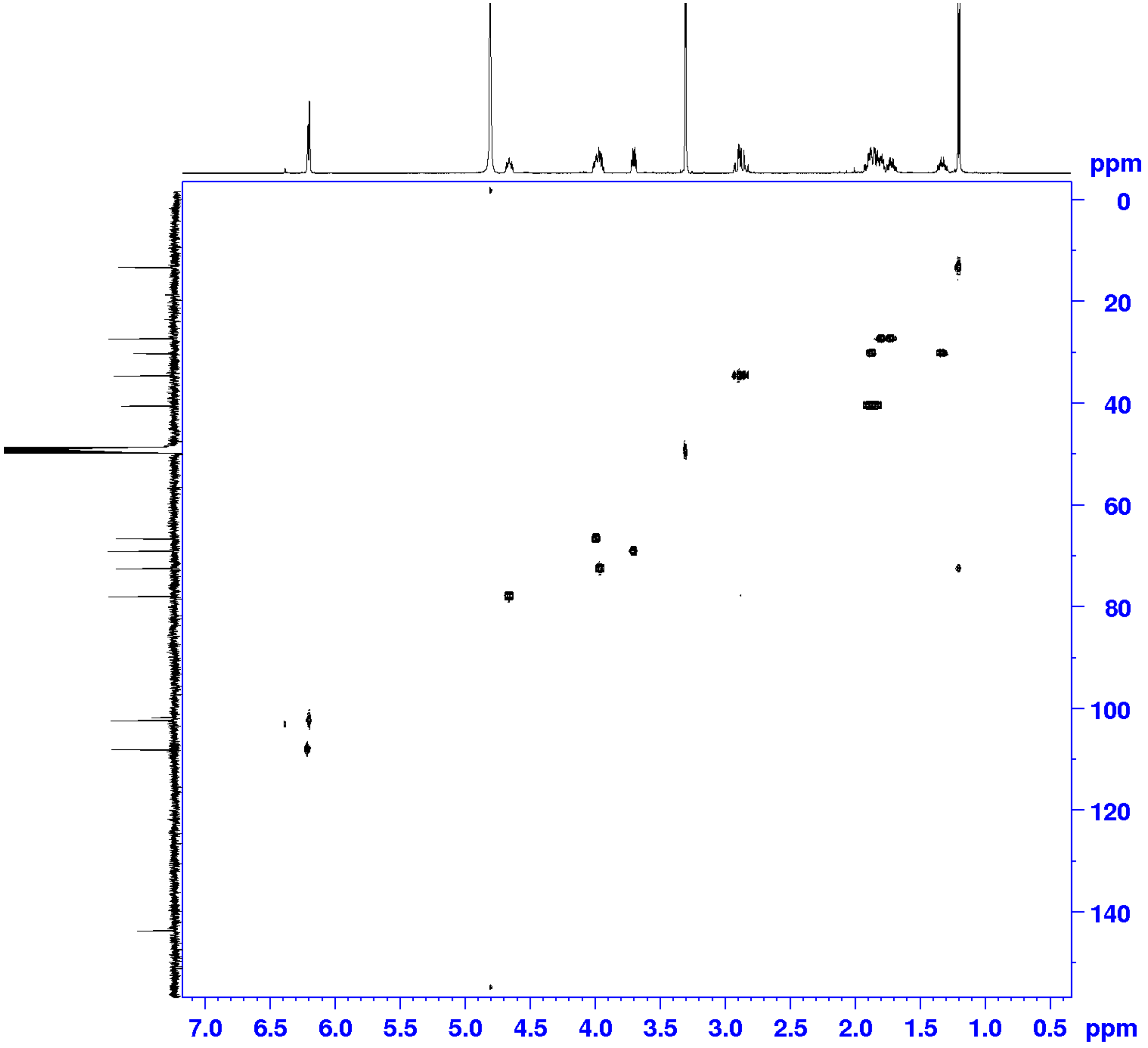

Figure S13. COSY spectrum of 2 measured in MeOD

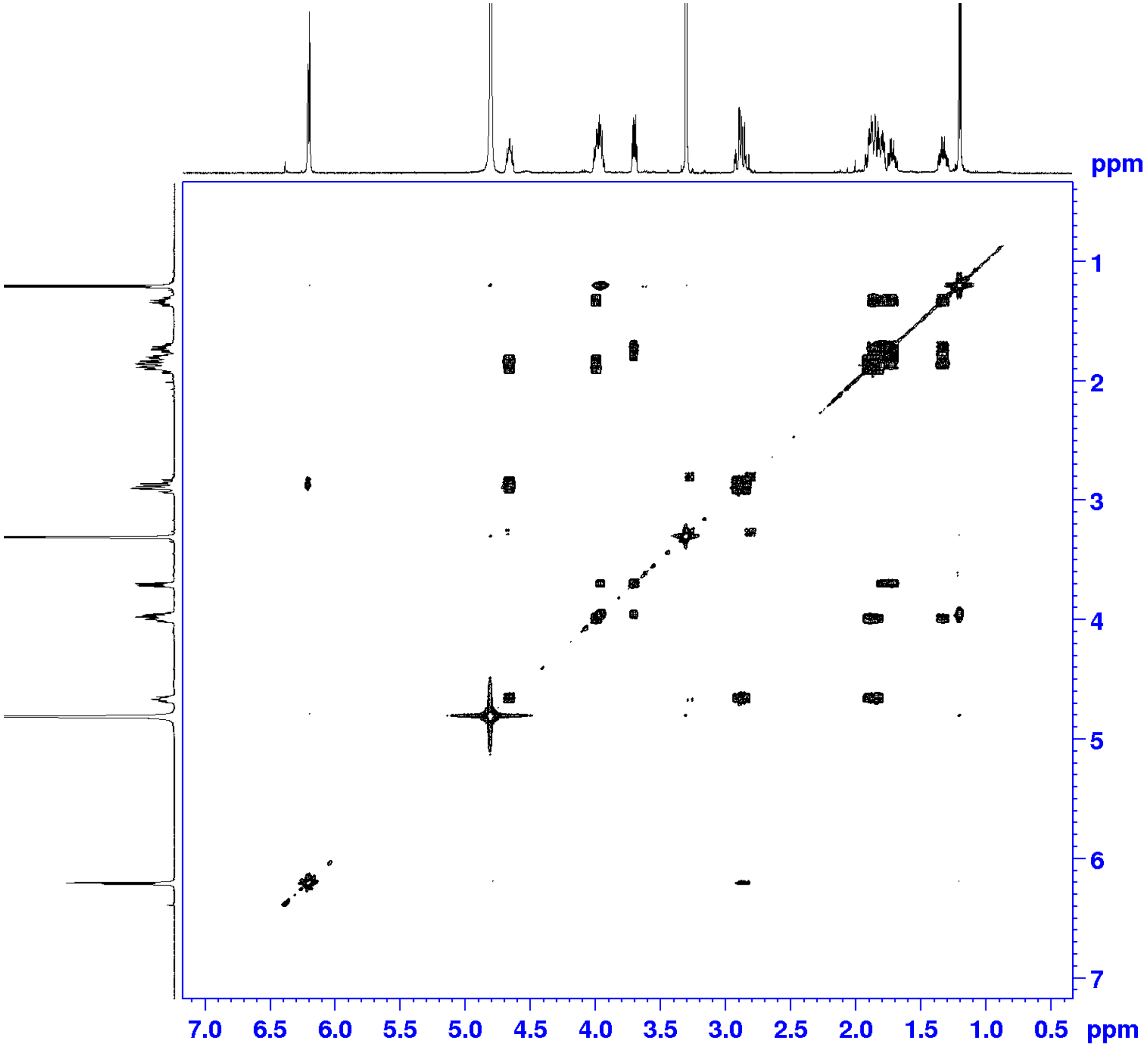

Figure S14. HMBC spectrum of 2 measured in DMSO-d6

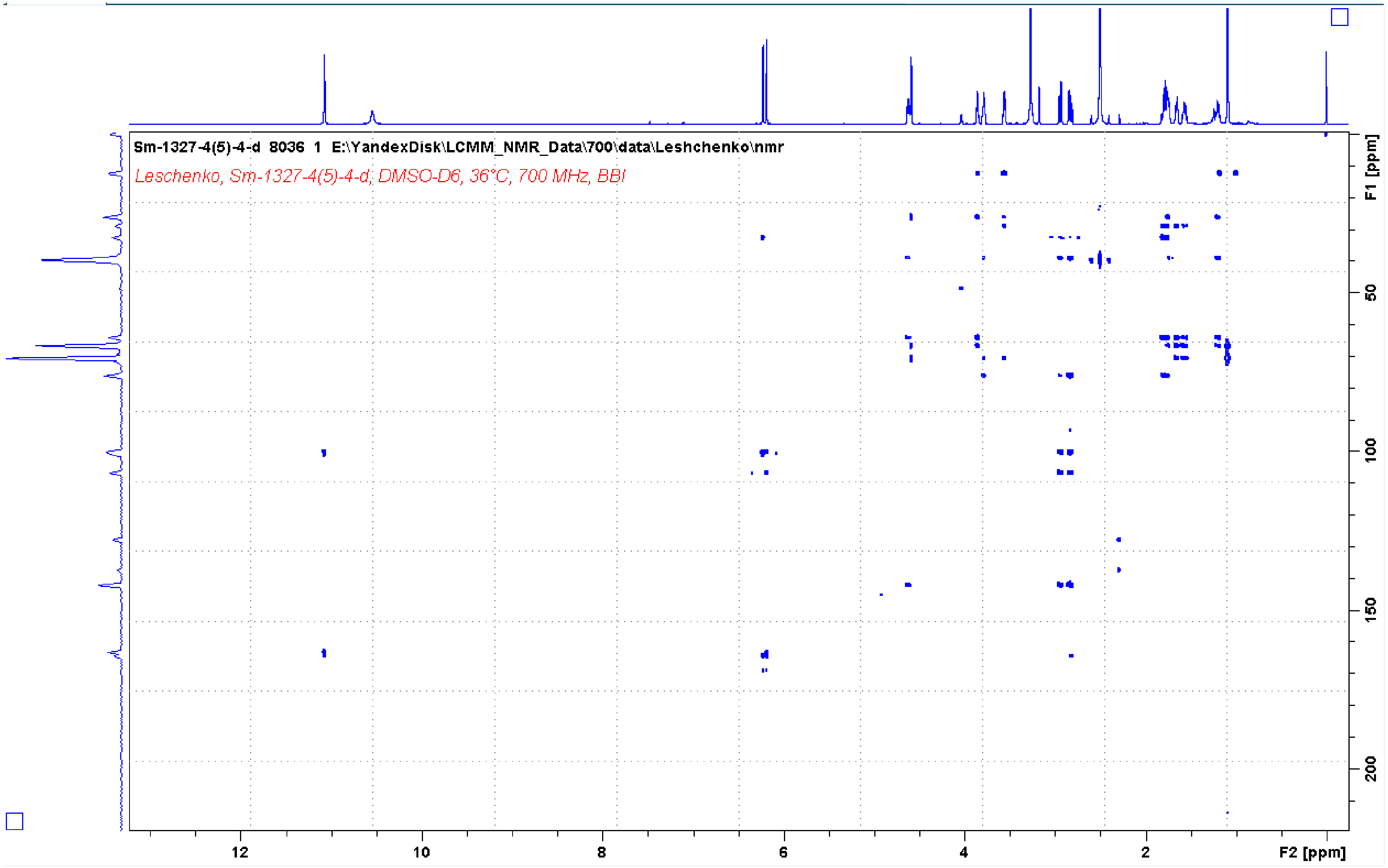

Figure S15. HRESIMS for 3

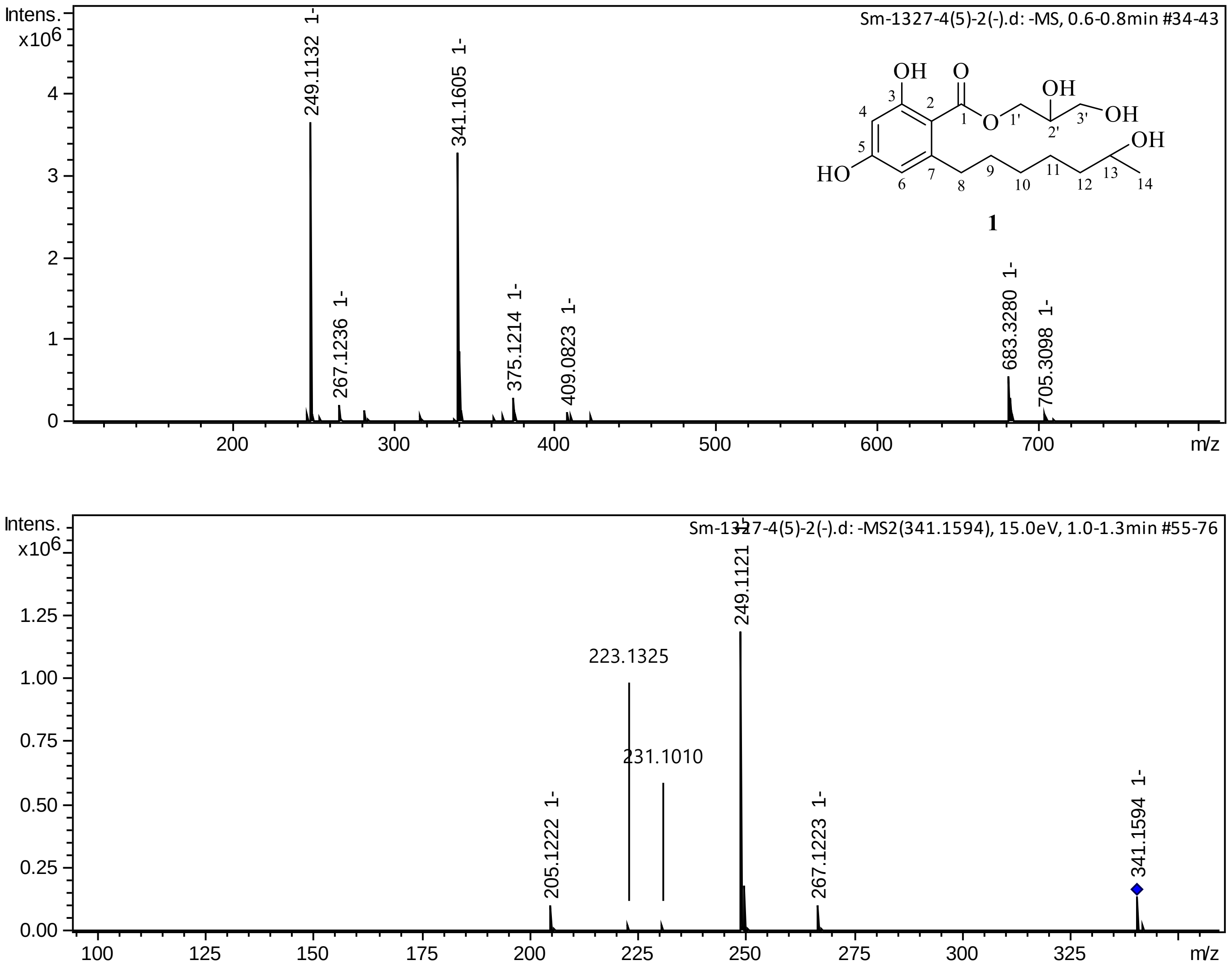

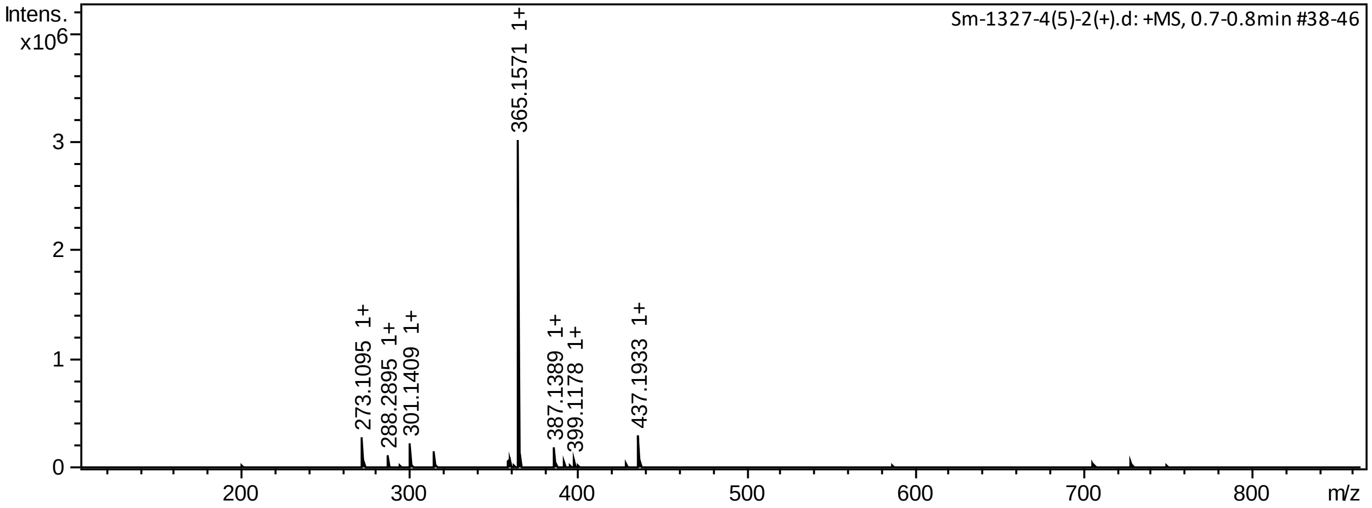

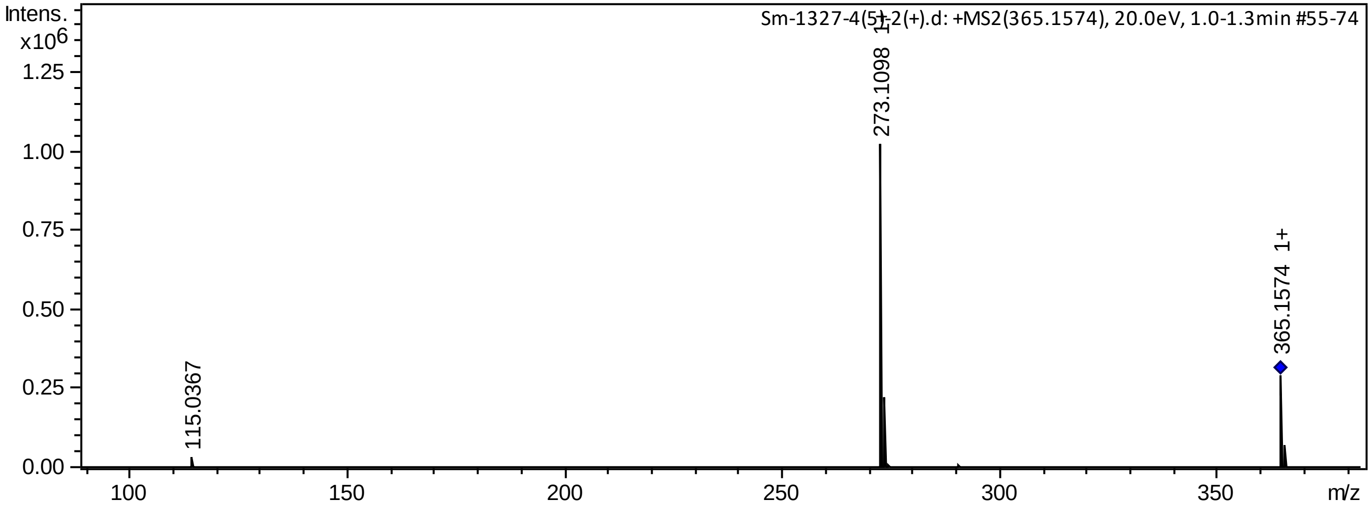

	meas	calc	Δ (ppm)
$[M-H]^-$	341,1605	341,1606	0,1
$[M+Na]^+$	365,1571	365,1571	-0,1

Figure S16. ¹H NMR spectrum of **3** measured at 500 MHz in MeOD

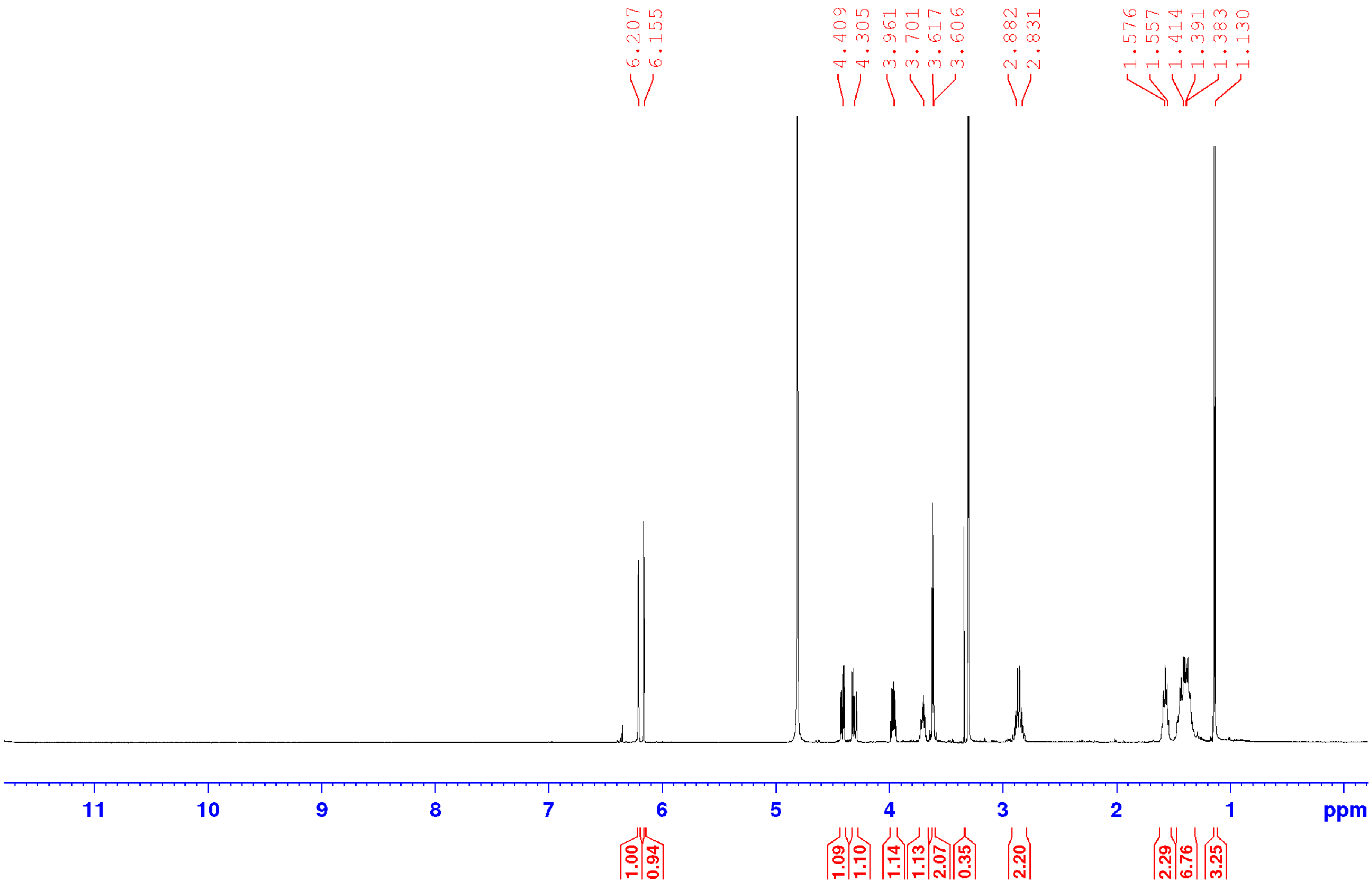

Figure S17. [13]**C NMR spectrum of 3 measured at 75 MHz in MeOD**

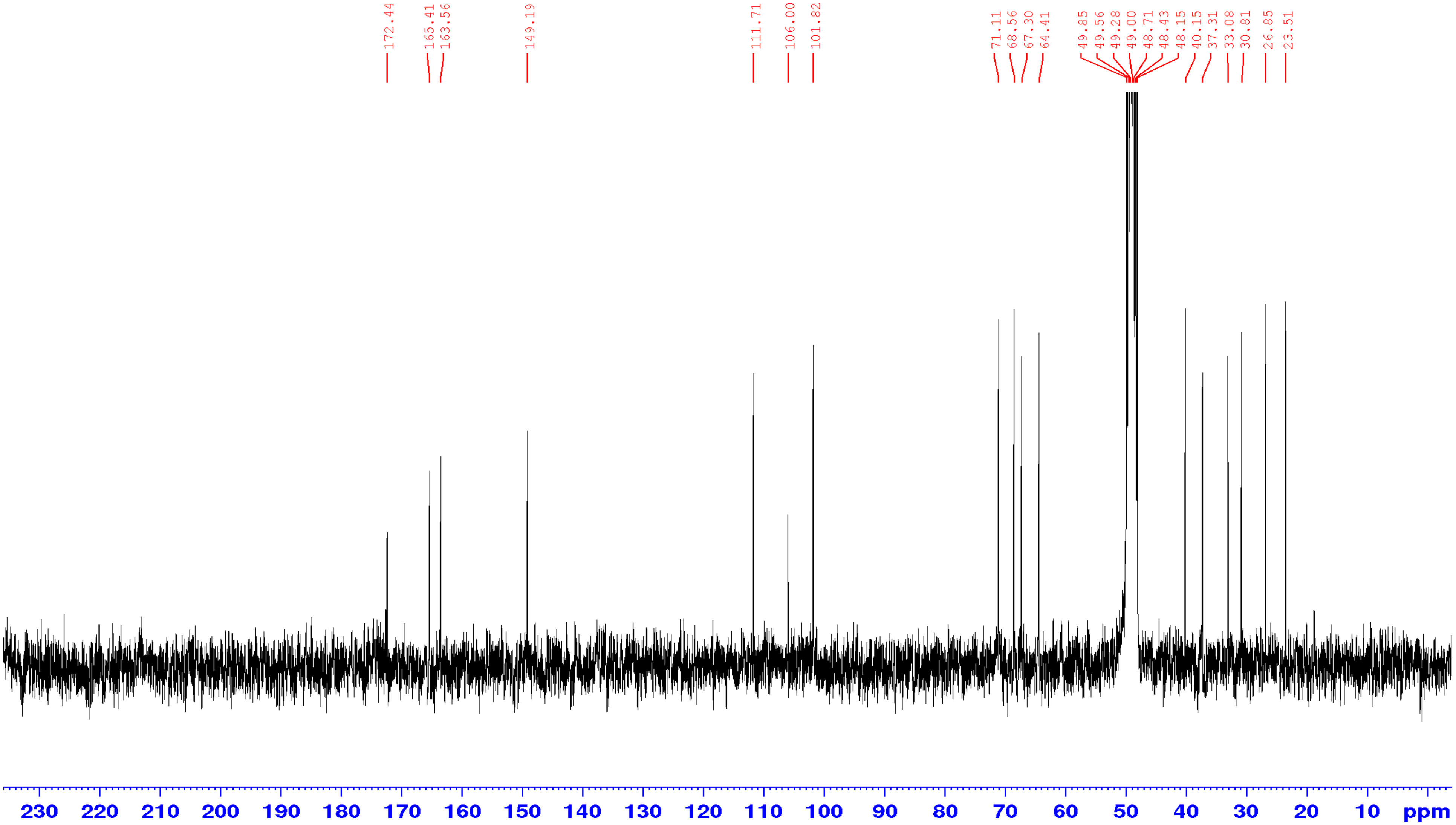

Figure S18. DEPT-135 spectrum of 3 measured at 75 MHz in MeOD

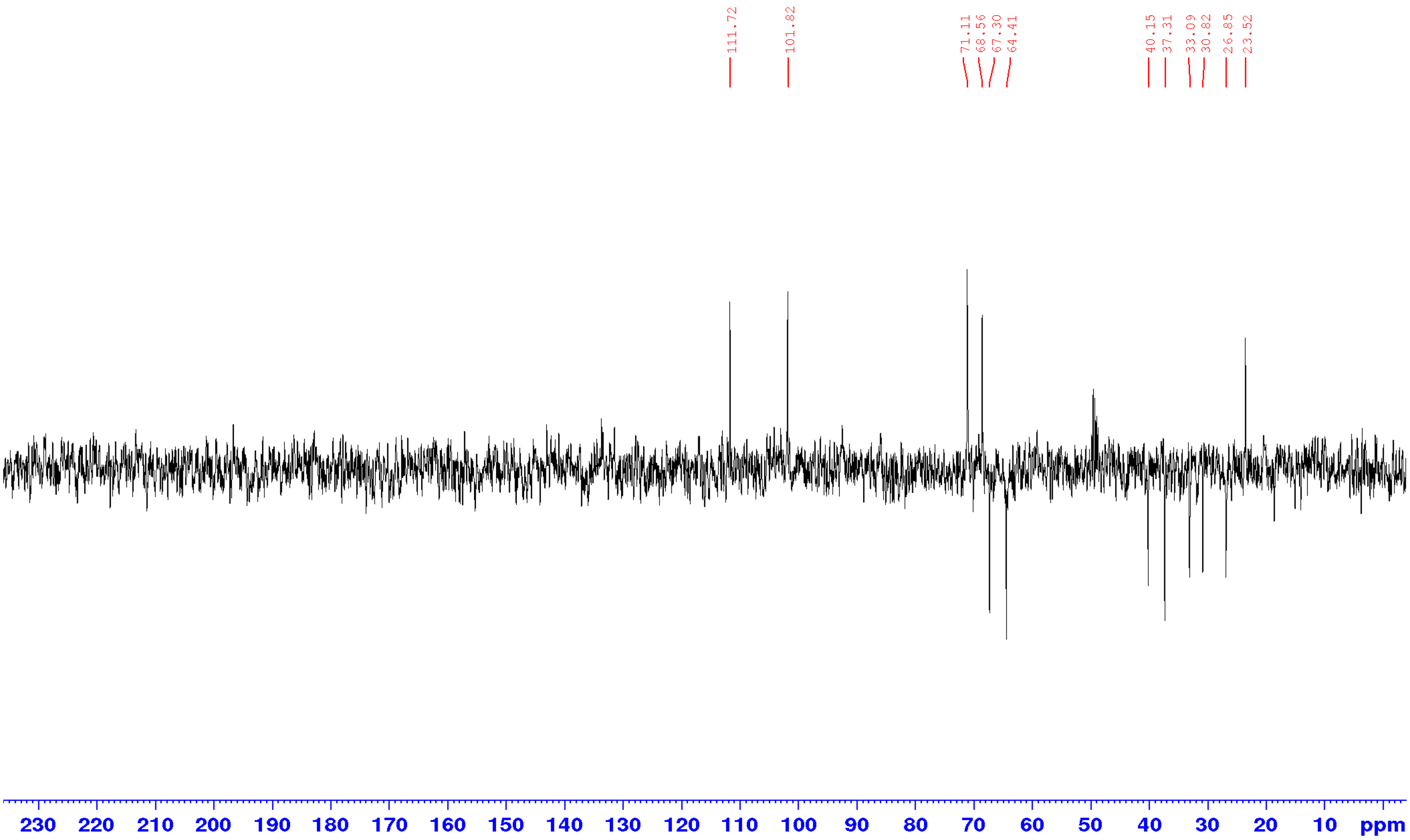

Figure S19. HSQC spectrum of 3 measured in MeOD

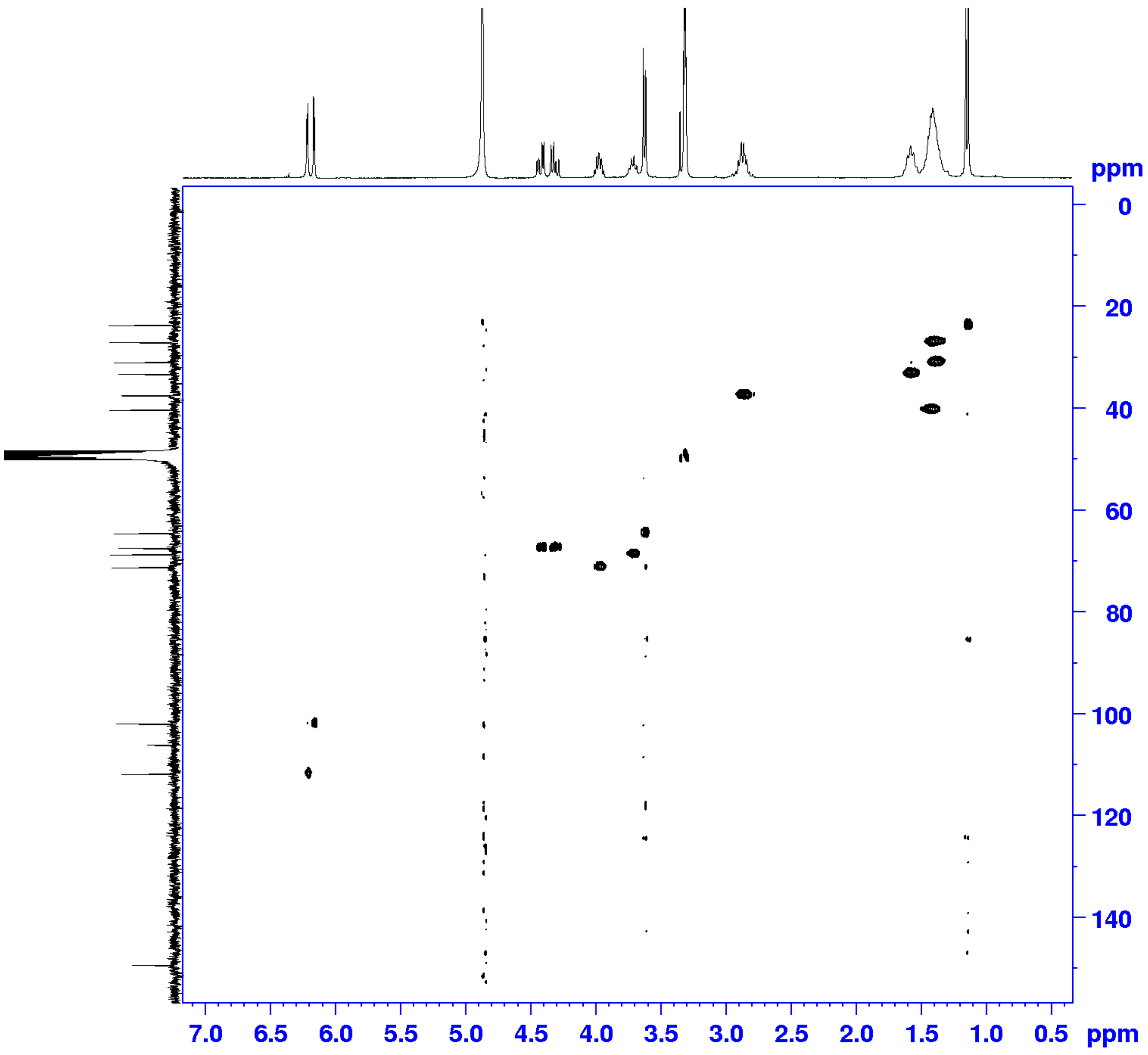

Figure S20. COSY spectrum of 3 measured in MeOD

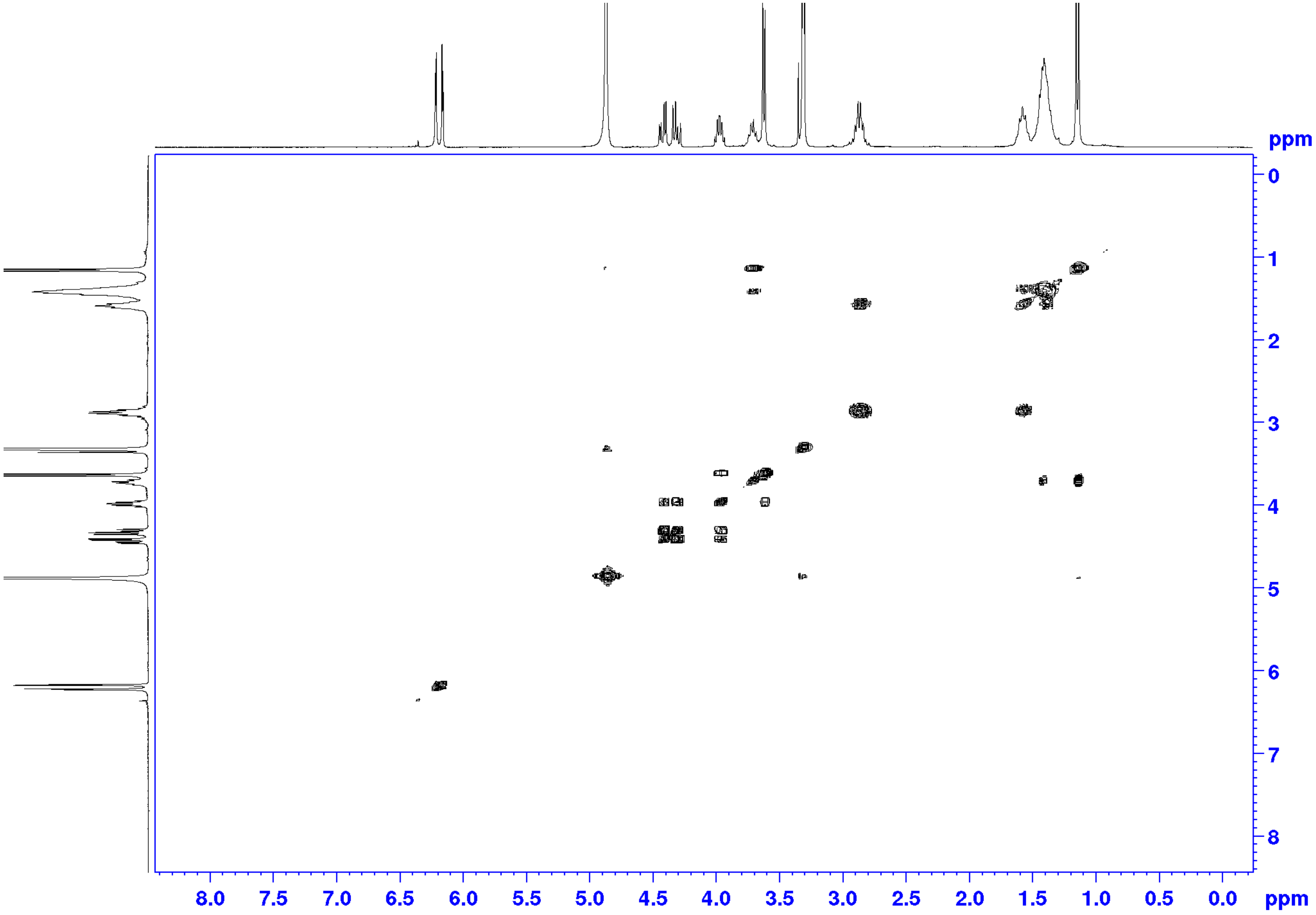

Figure S21. HMBC spectrum of 3 measured in MeOD

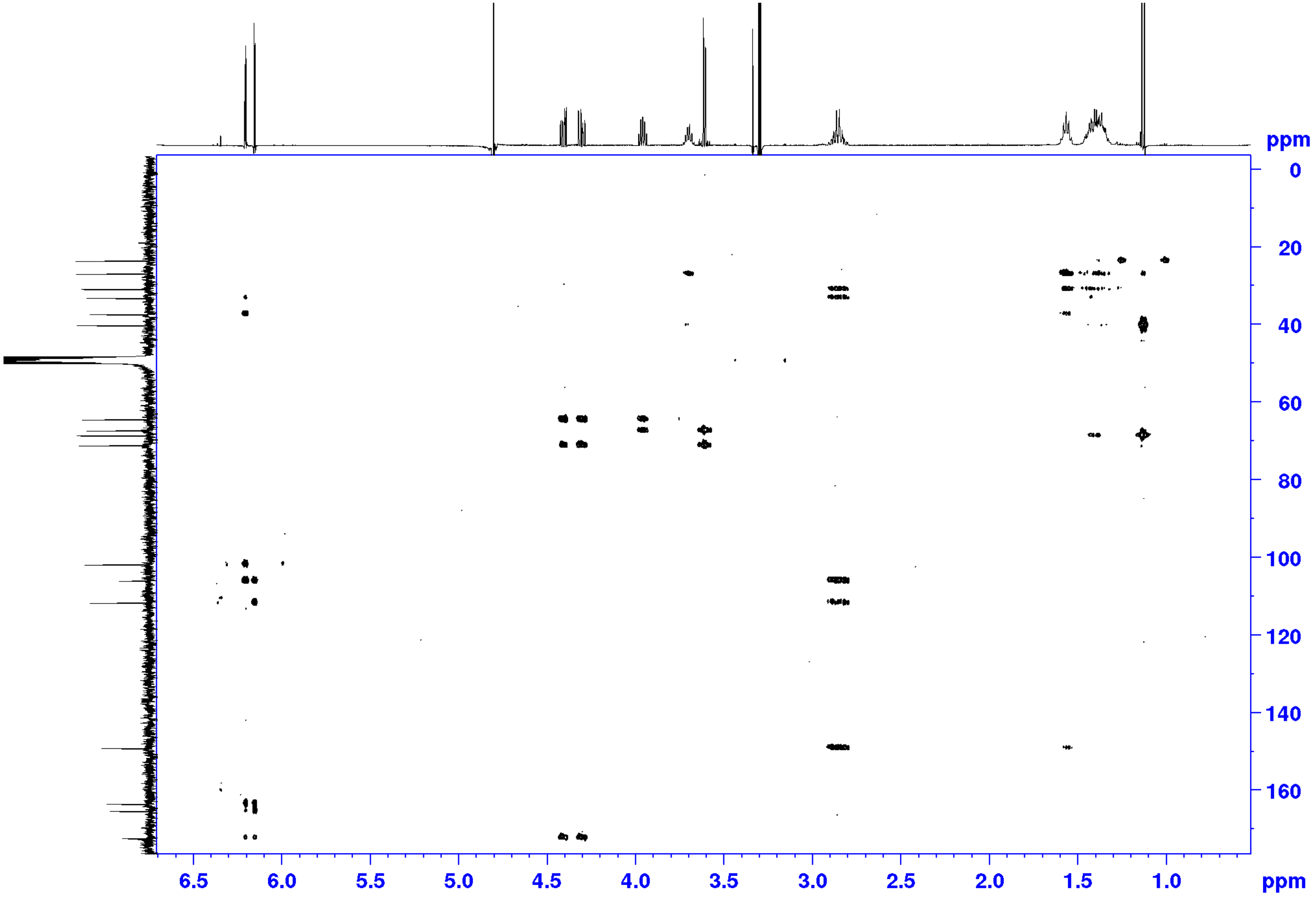

Figure S22. HRESIMS for 4

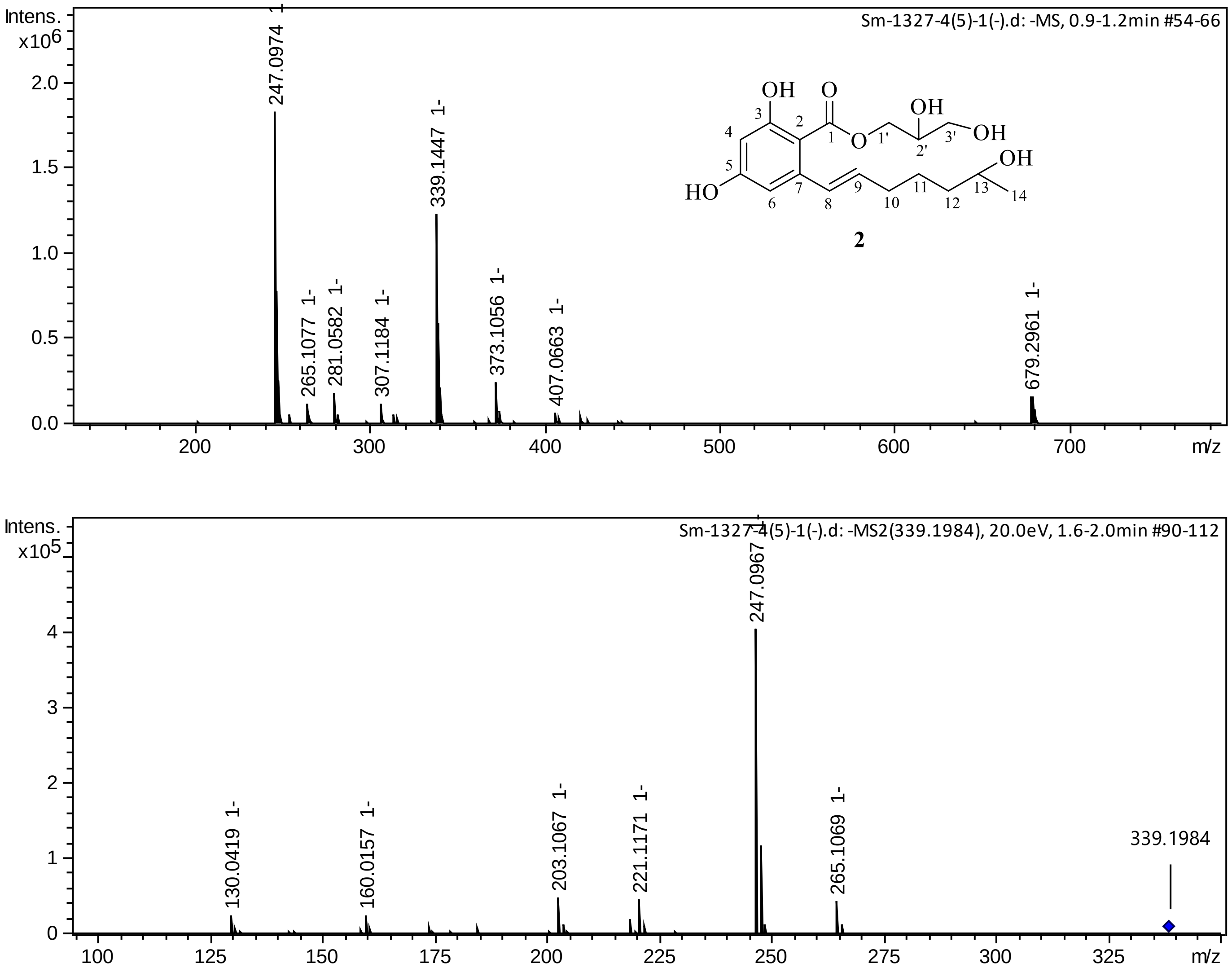

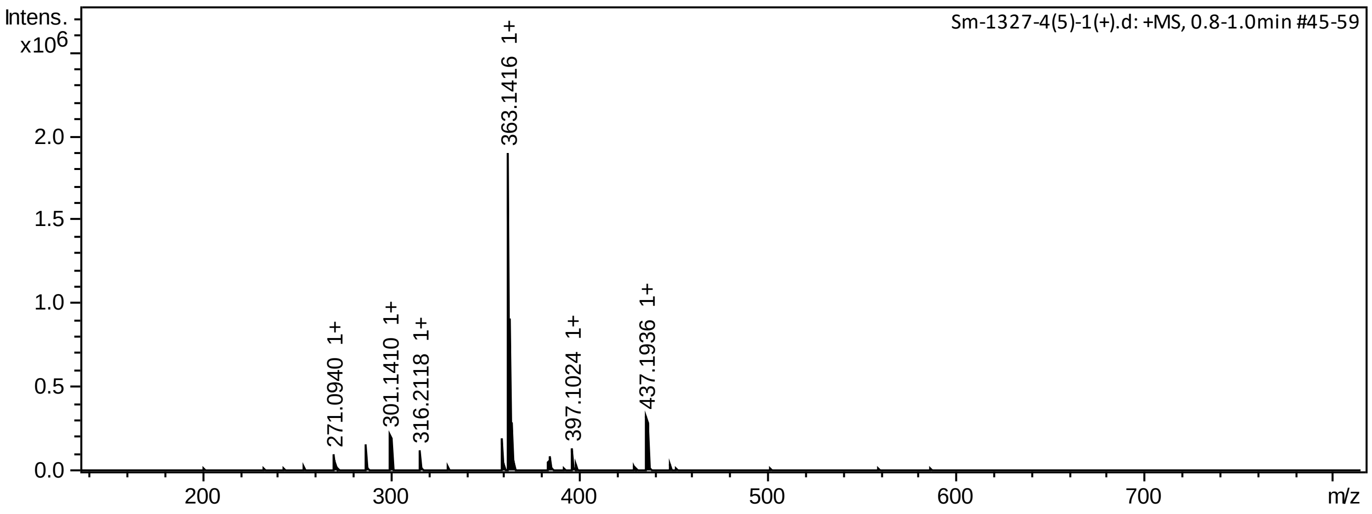

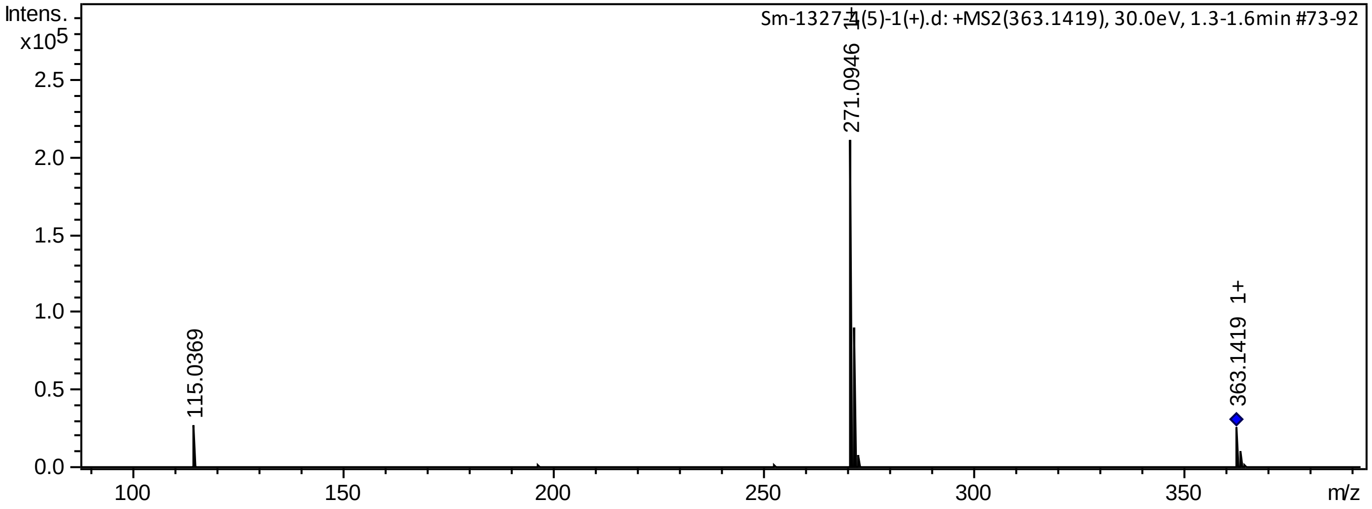

	meas	calc	Δ (ppm)
$[M-H]^-$	339,1447	339,1449	0,6
$[M+Na]^+$	363,1416	363,1414	-0,5

Figure S23. ^1H **NMR spectrum of 4 measured at 500 MHz in MeOD**

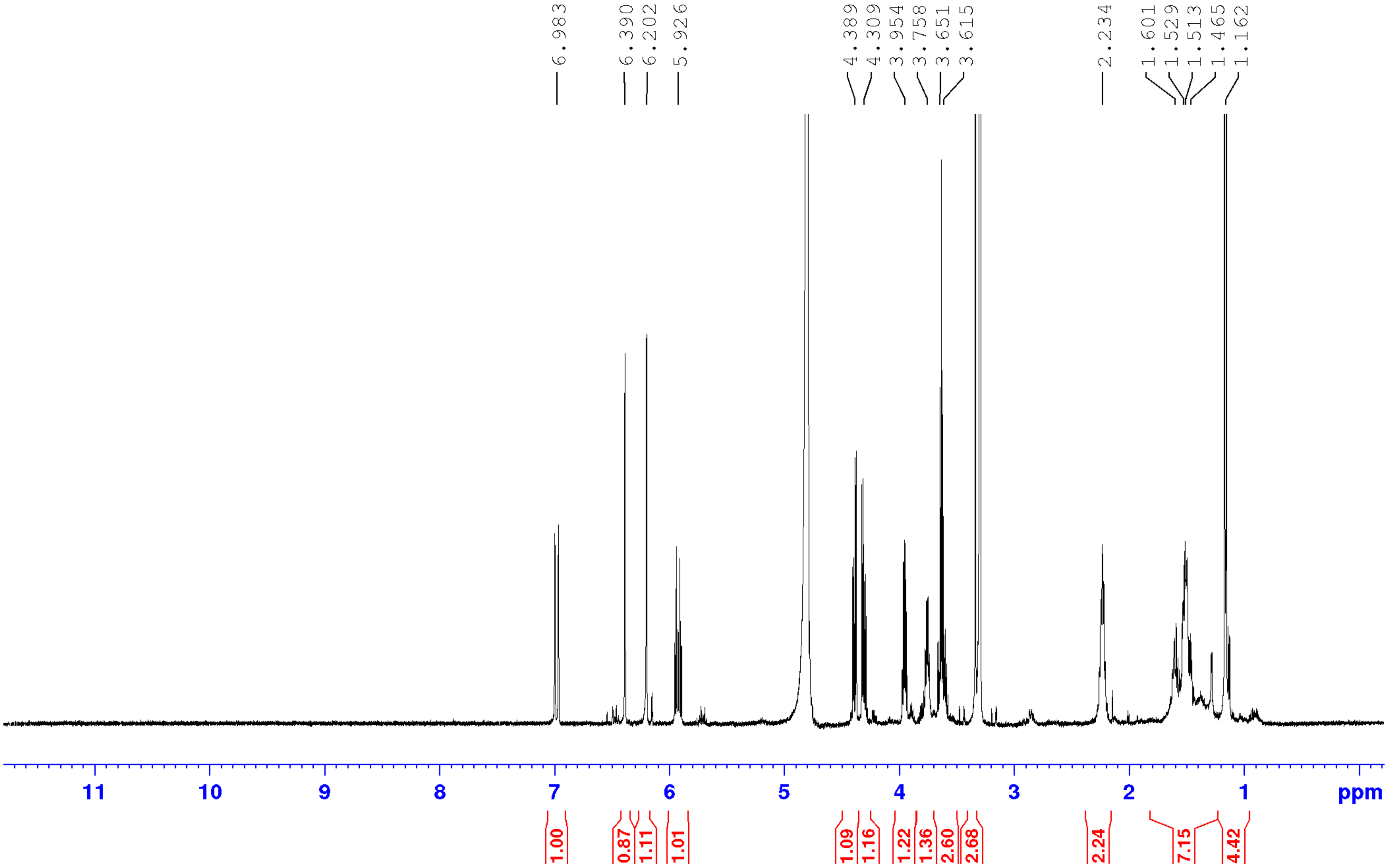

Figure S23. ^{13}C **NMR spectrum of 4 measured at 75 MHz in MeOD**

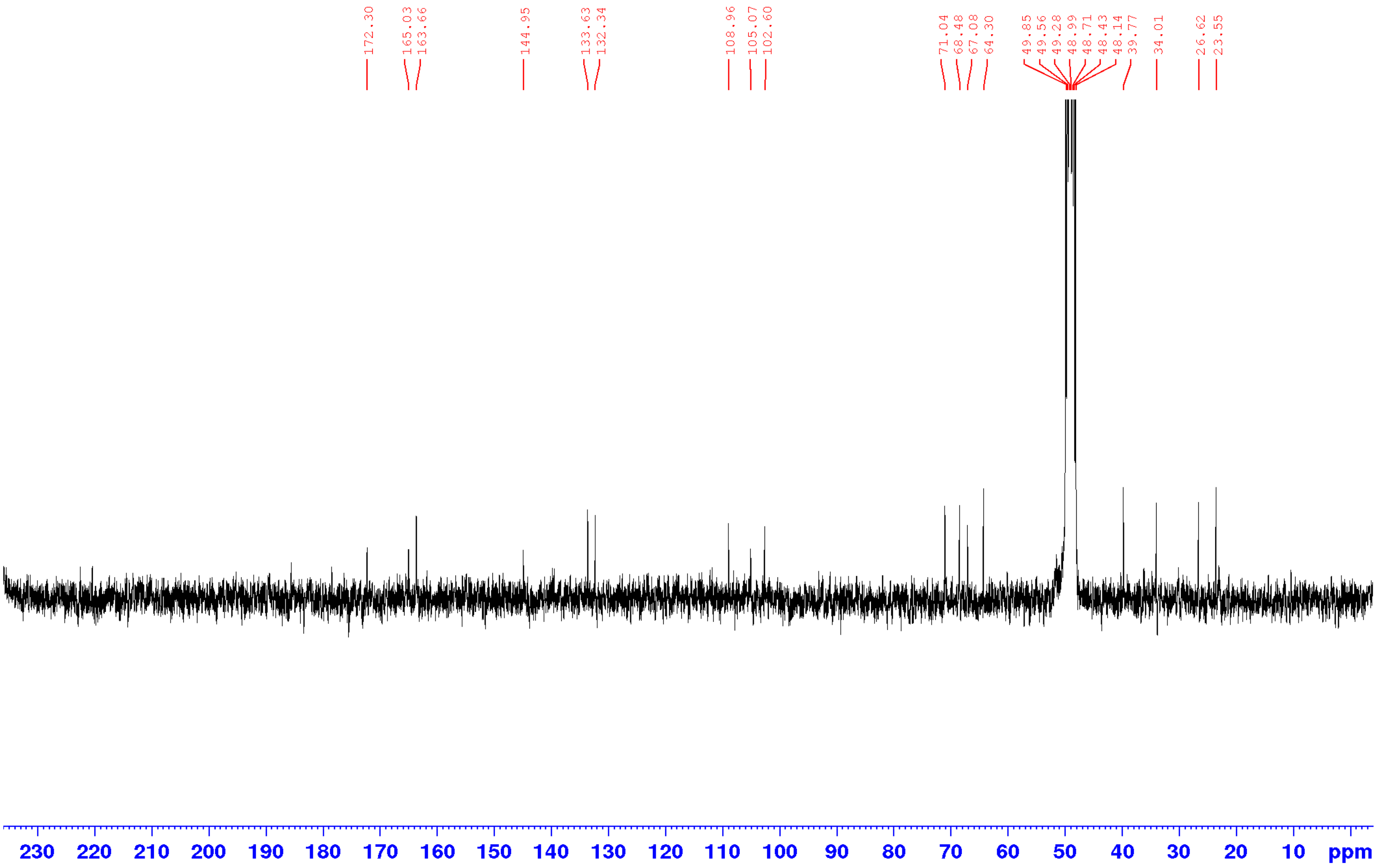

Figure S25. ^{13}**C NMR spectrum of 4 measured at 75 MHz in MeOD**

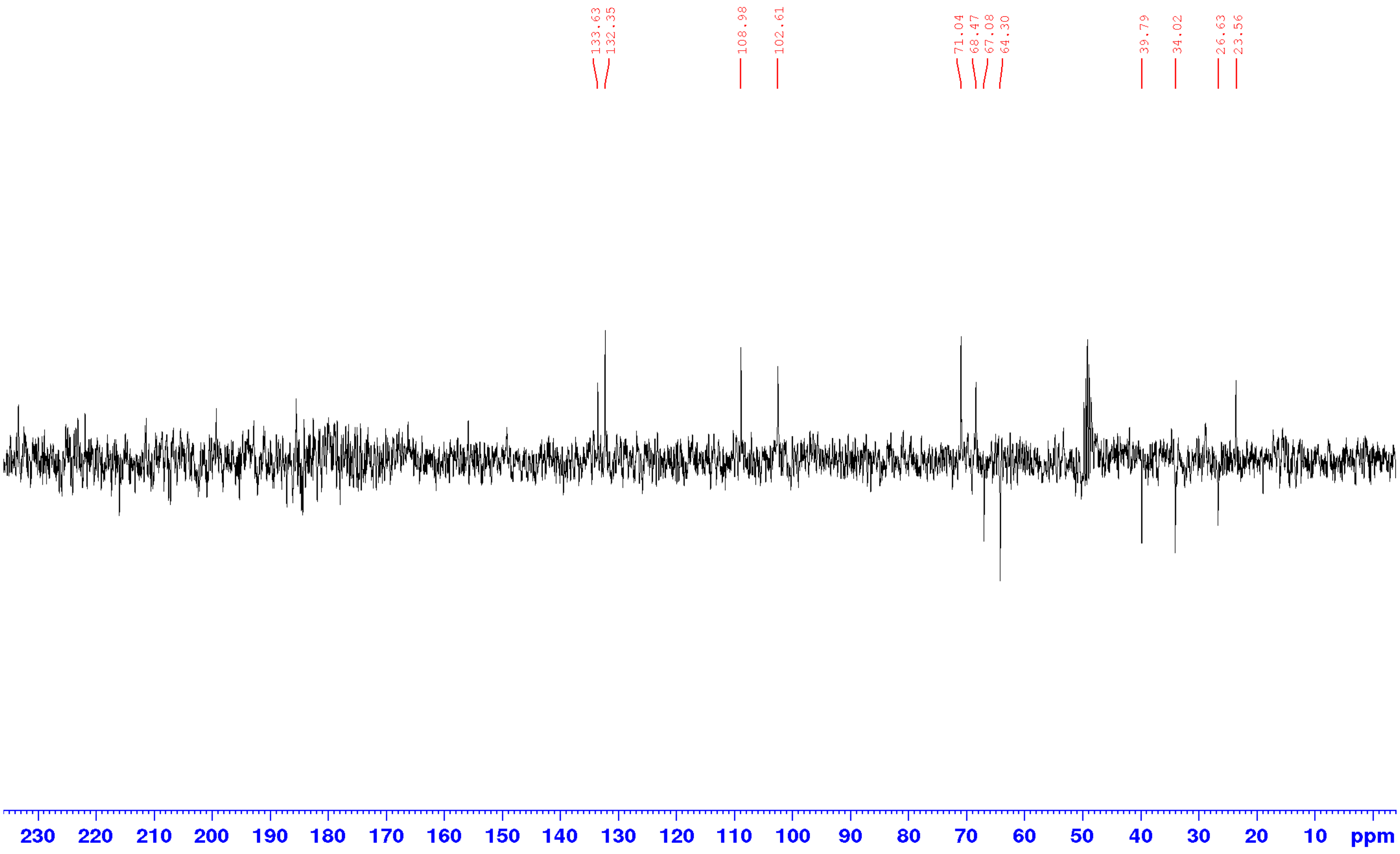

Figure S26. HSQC spectrum of 4 measured in MeOD

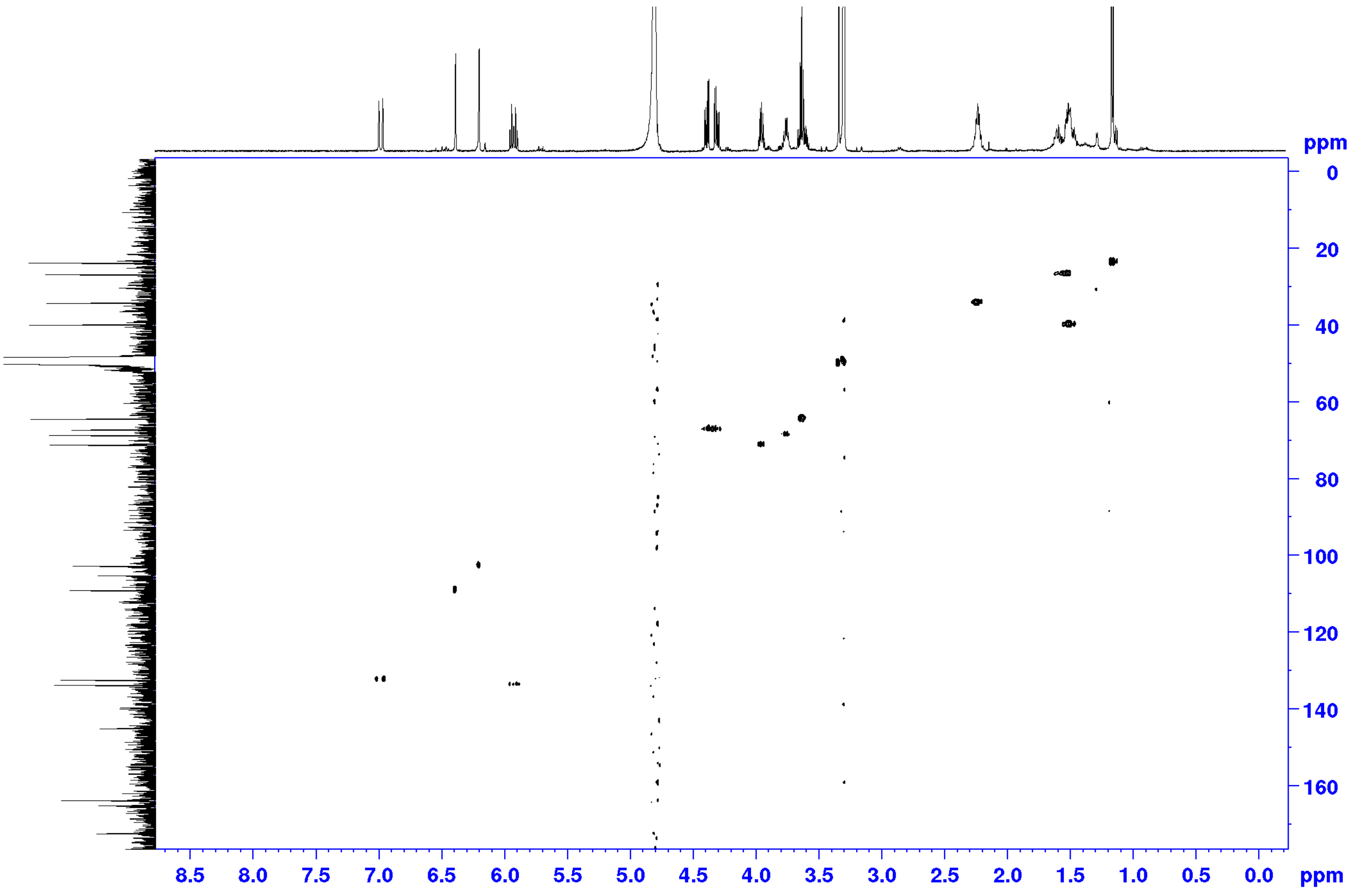

Figure S27. COSY spectrum of 4 measured in MeOD

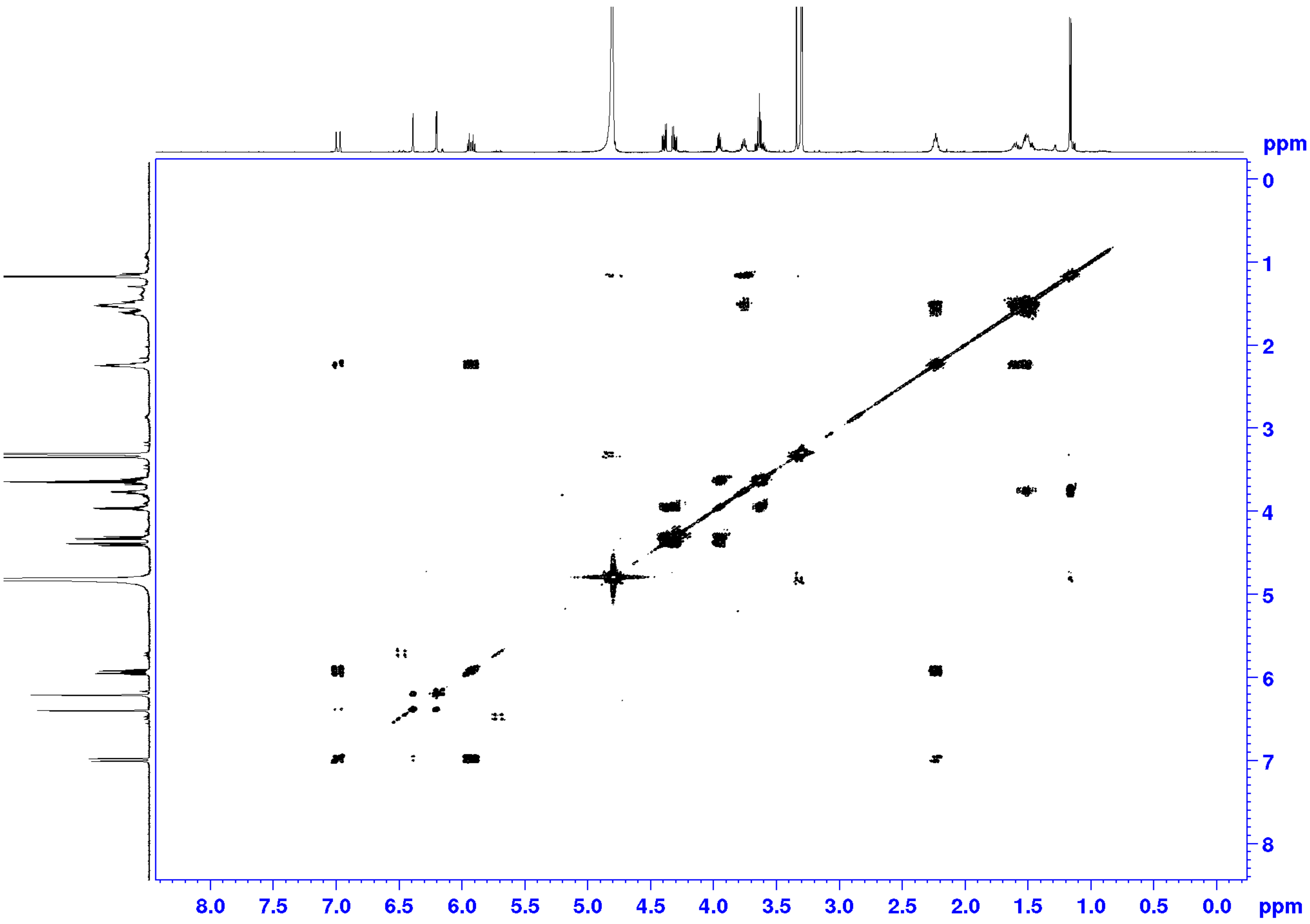

Figure S28. HMBC spectrum of 4 in MeOD

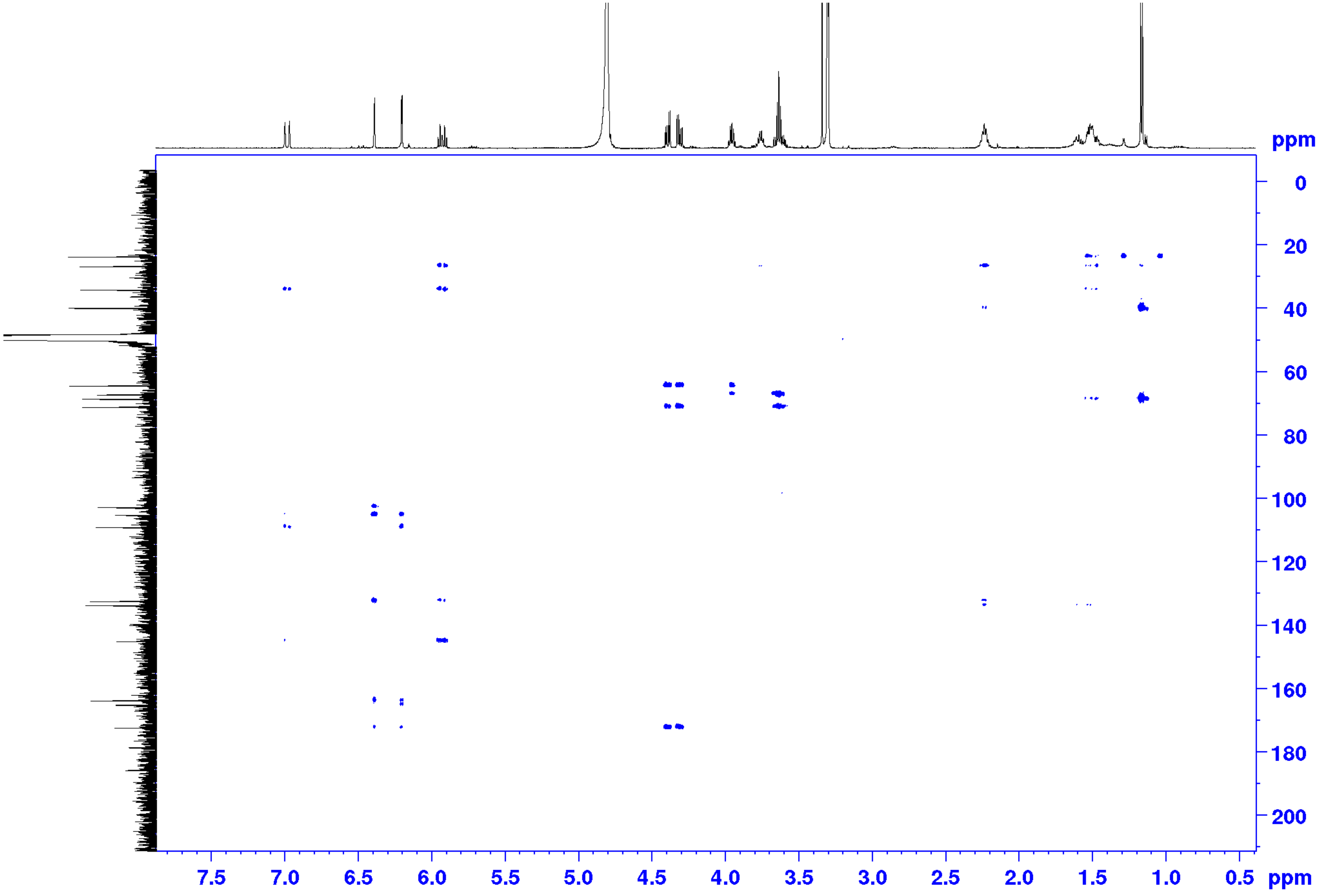

Figure S29. HRESIMS for 5

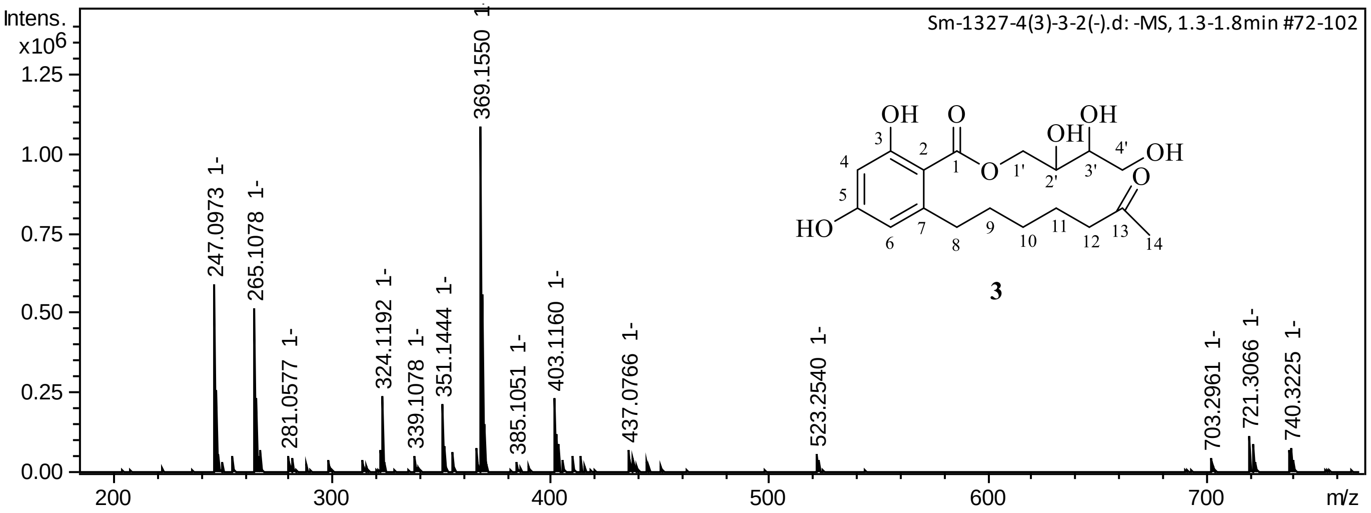

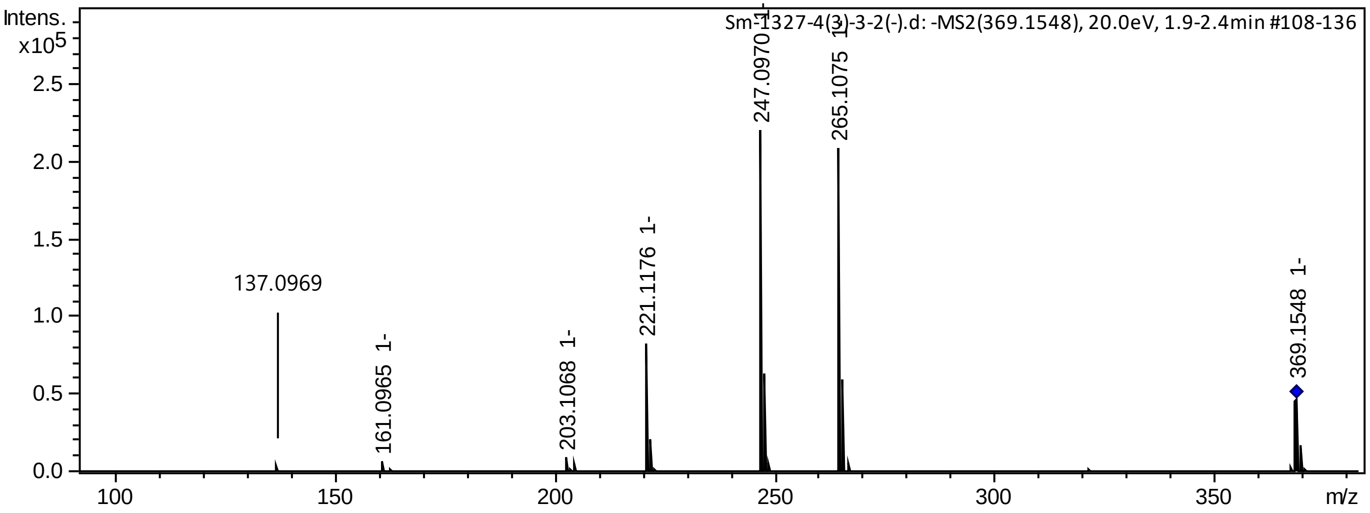

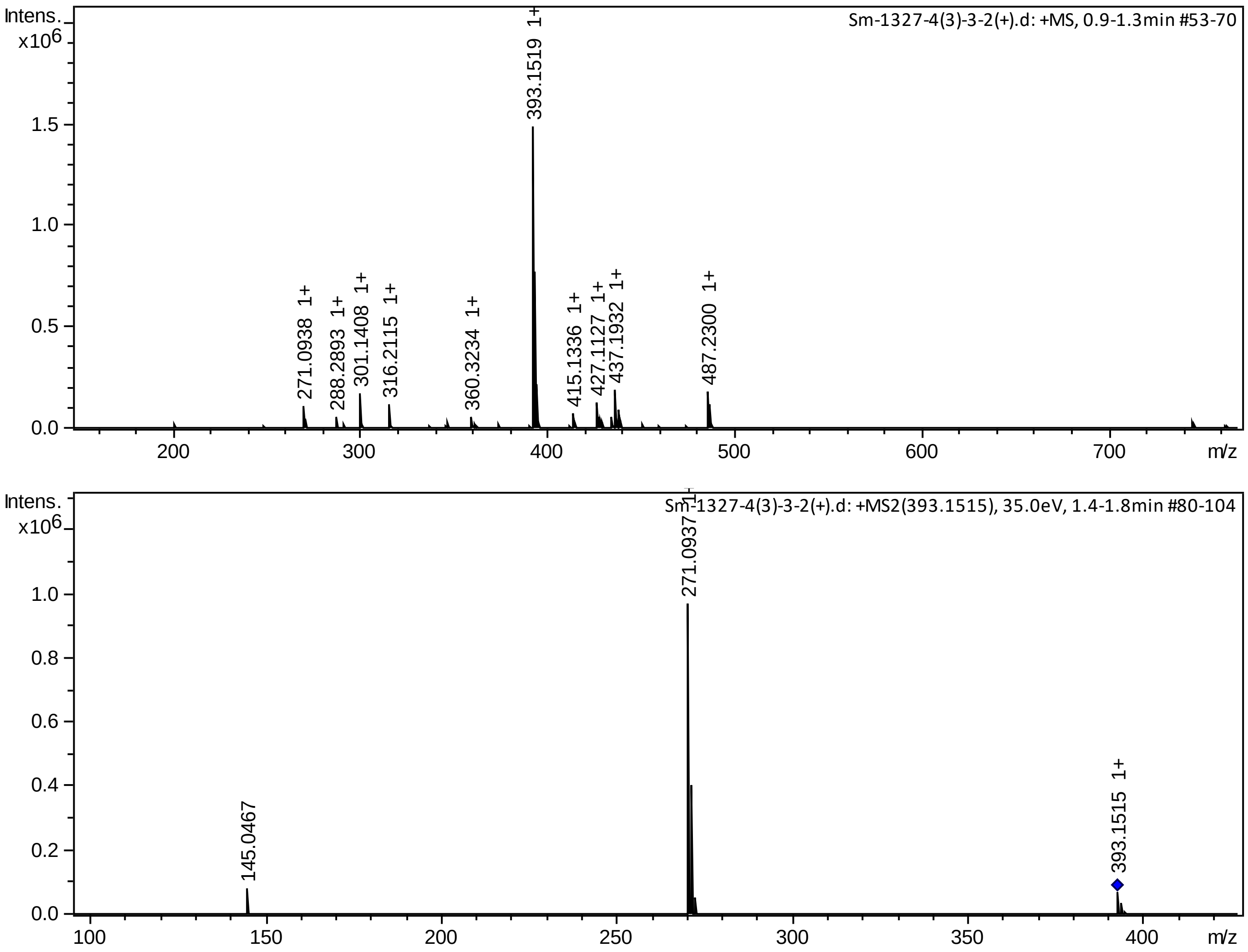

	meas	calc	Δ (ppm)
$[M-H]^-$	369,1550	369,1555	1,3
$[M+Na]^+$	393,1519	393,152	0,3

Figure S30. ^{1}H NMR spectrum of **5** measured at 500 MHz in MeOD

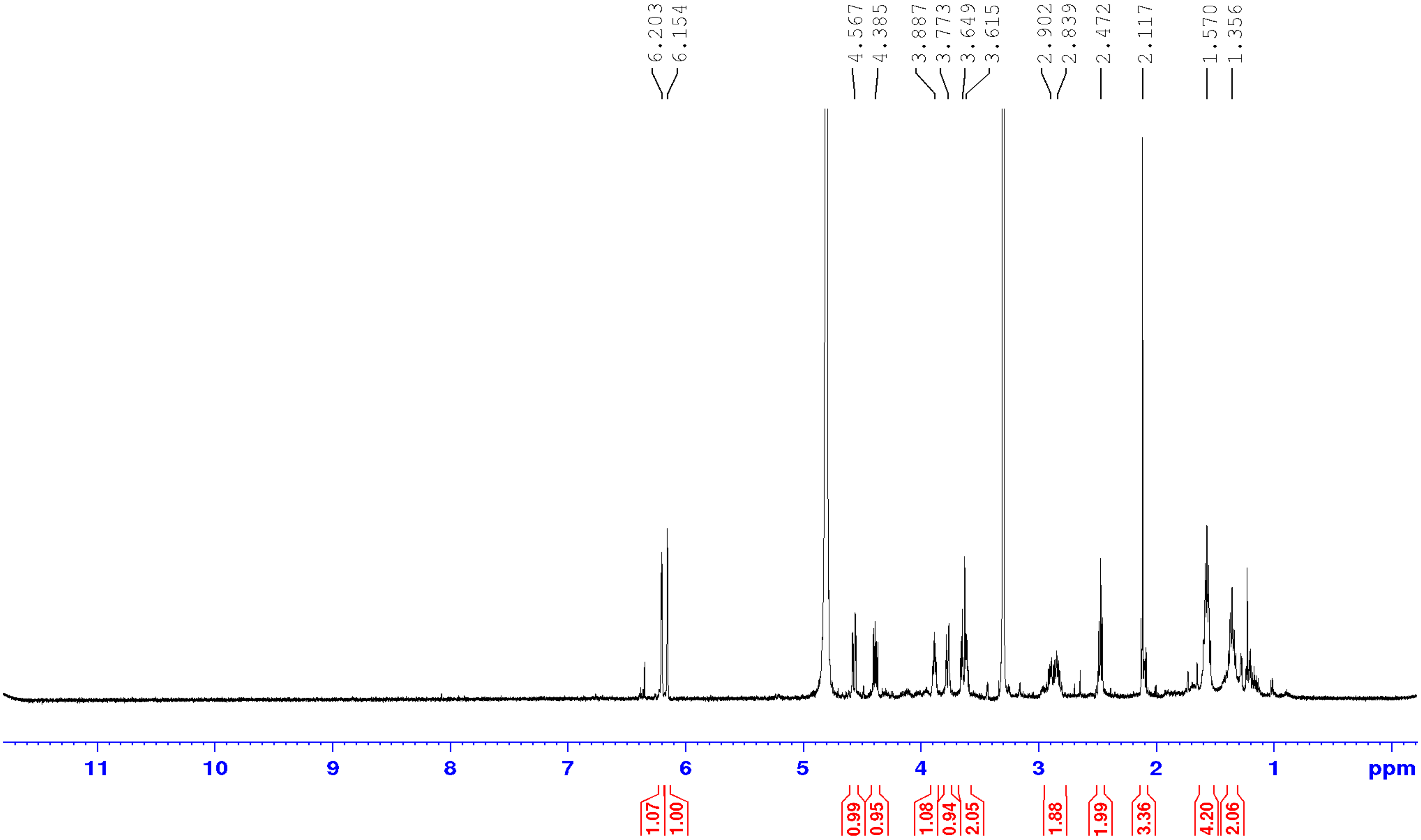

Figure S31. ^{13}C NMR spectrum of 5 measured at 125 MHz in MeOD

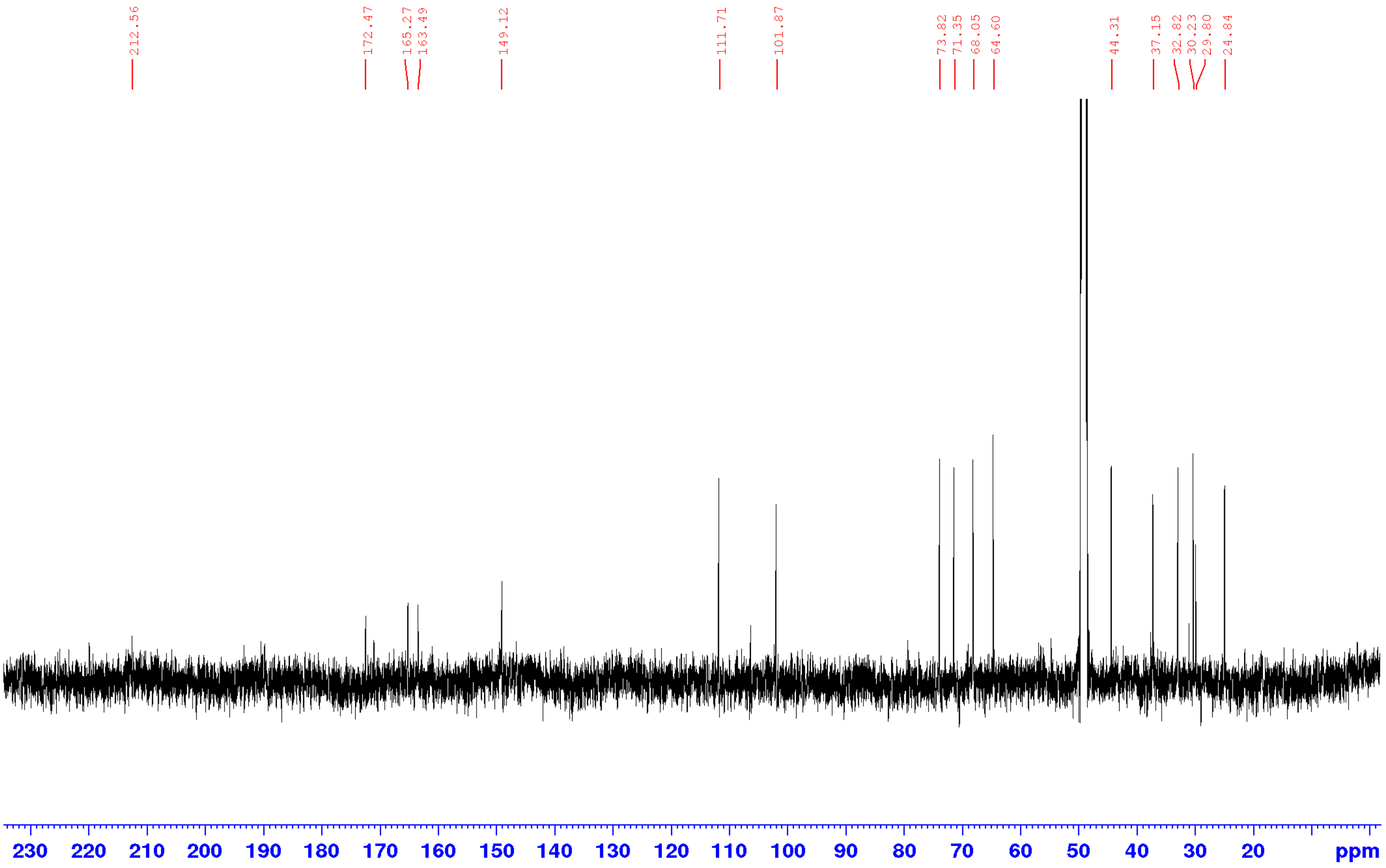

Figure S32. DEPT spectrum of 5 measured at 125 MHz in MeOD

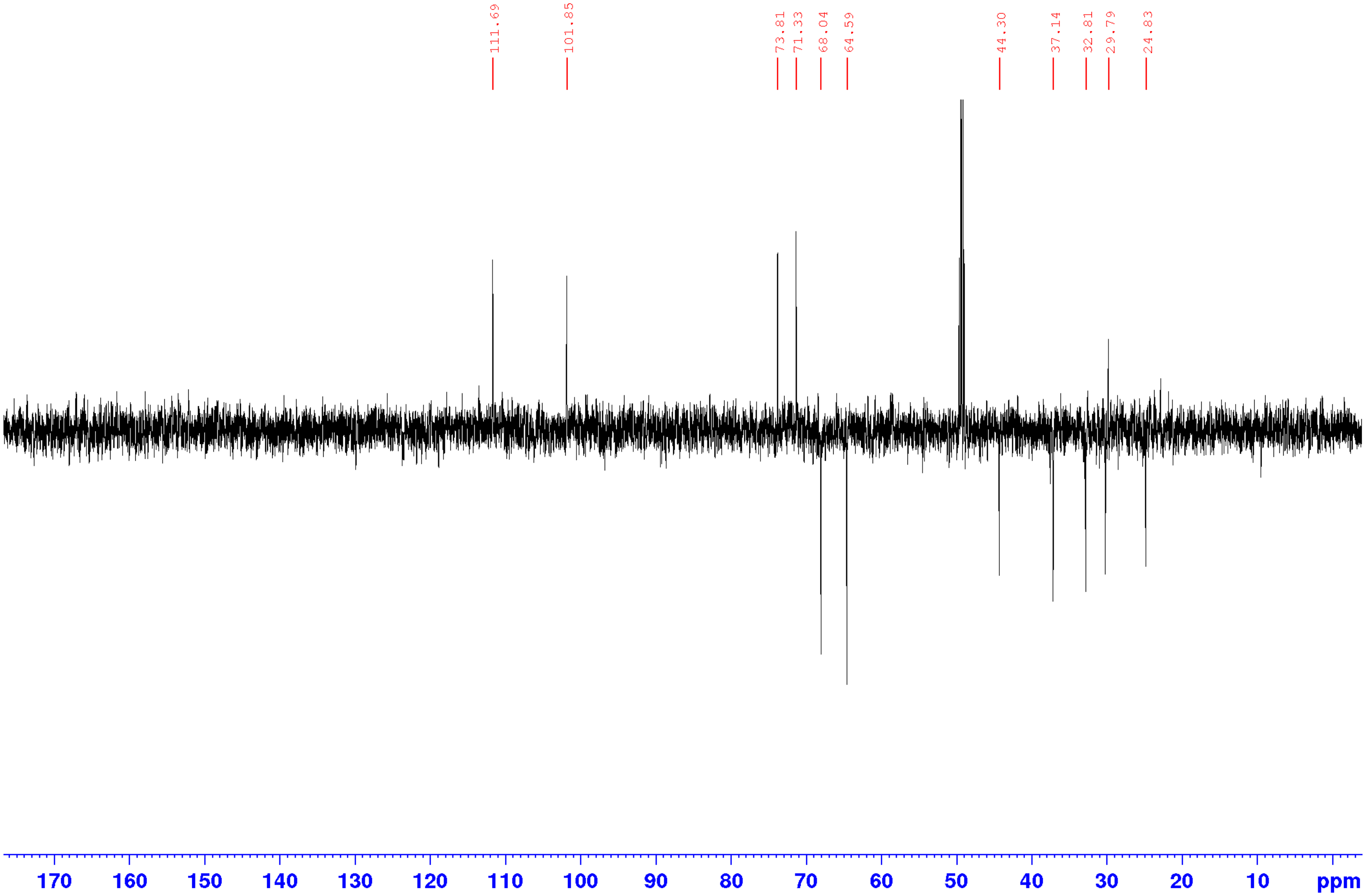

Figure S33. HSQC spectrum of 5 measured in MeOD

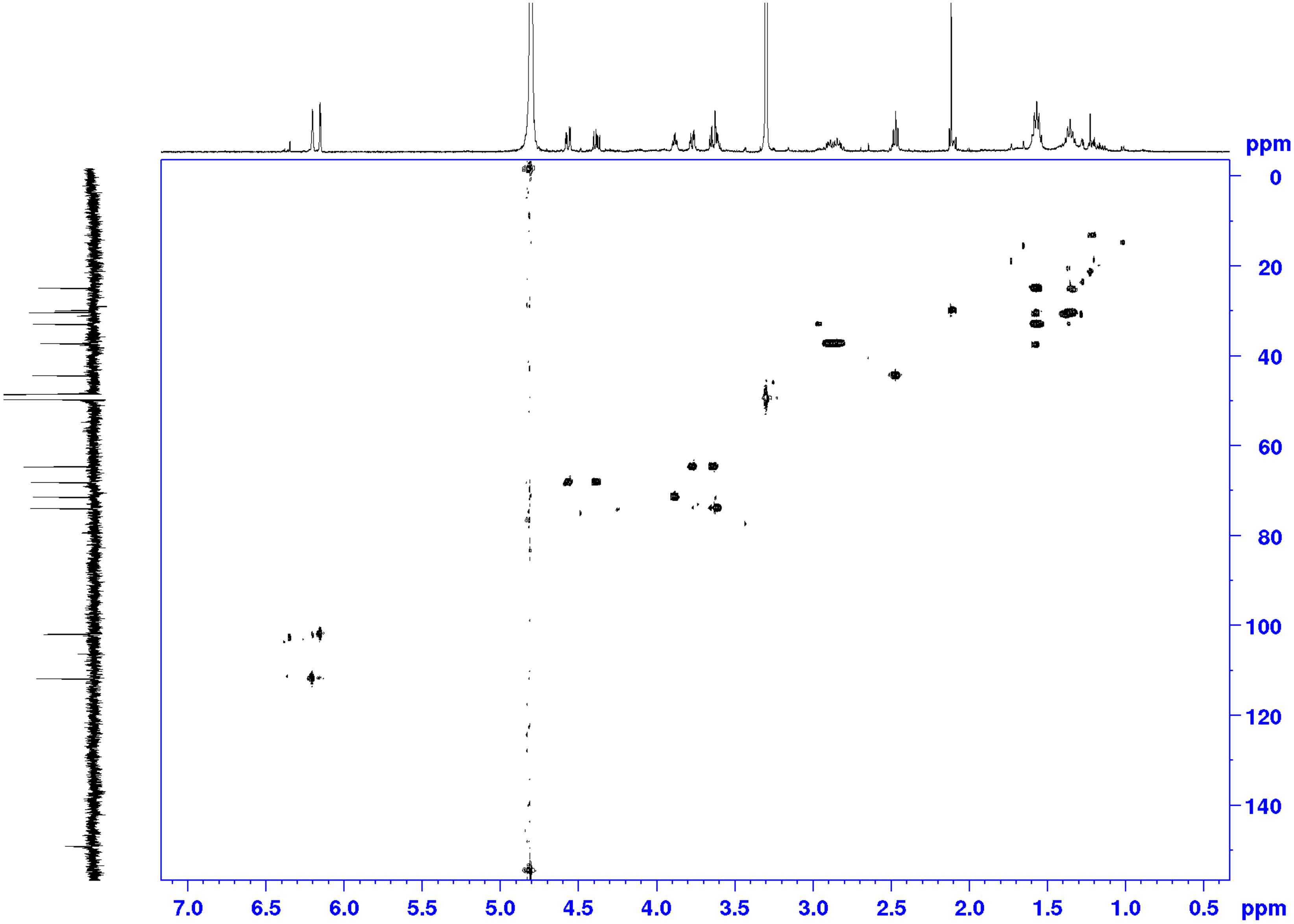

Figure S34. HMBC spectrum of 5 measured in MeOD

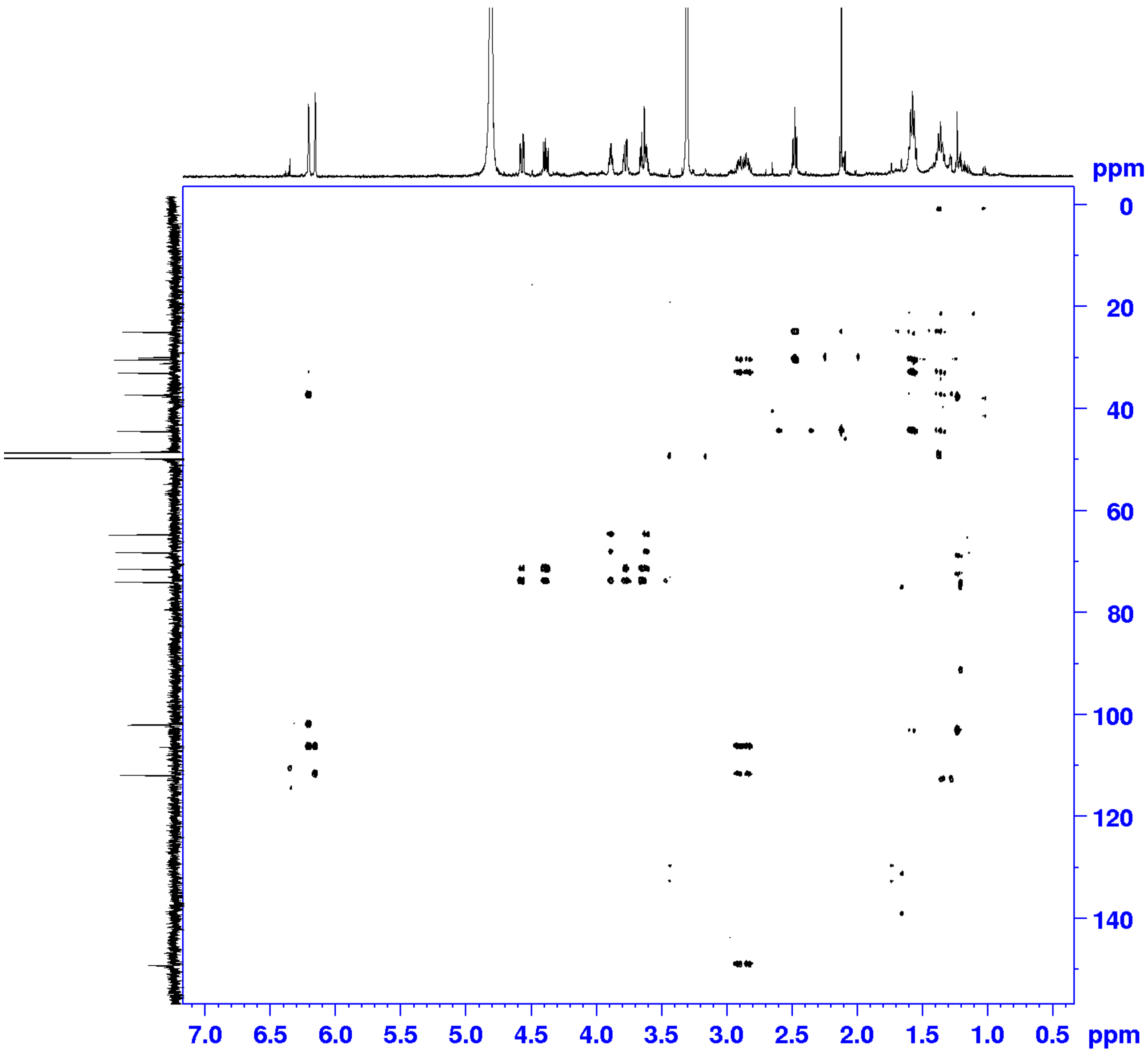

Figure S35. COSY spectrum of 5 measured in MeOD

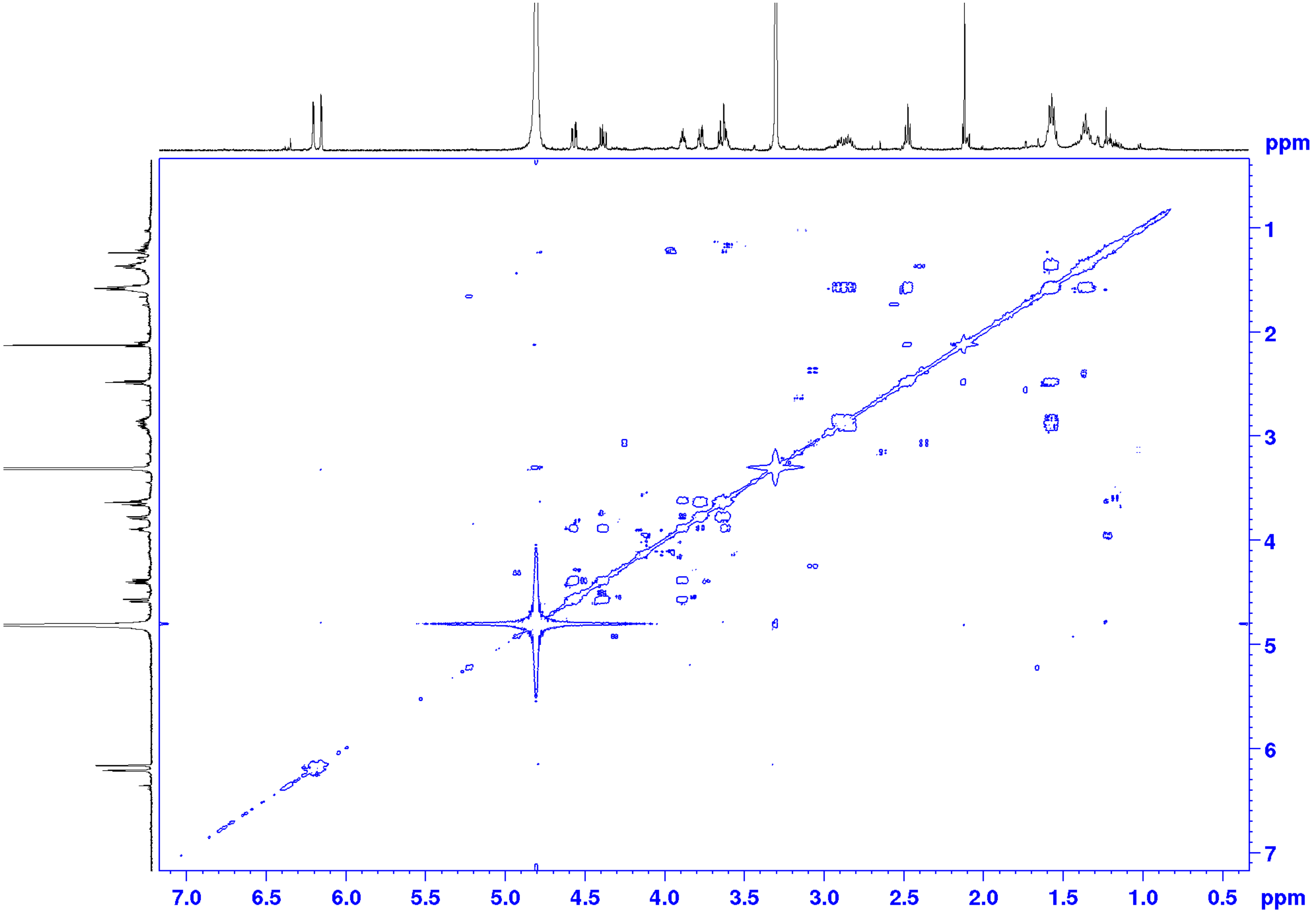

Figure S36. HRESIMS for 6

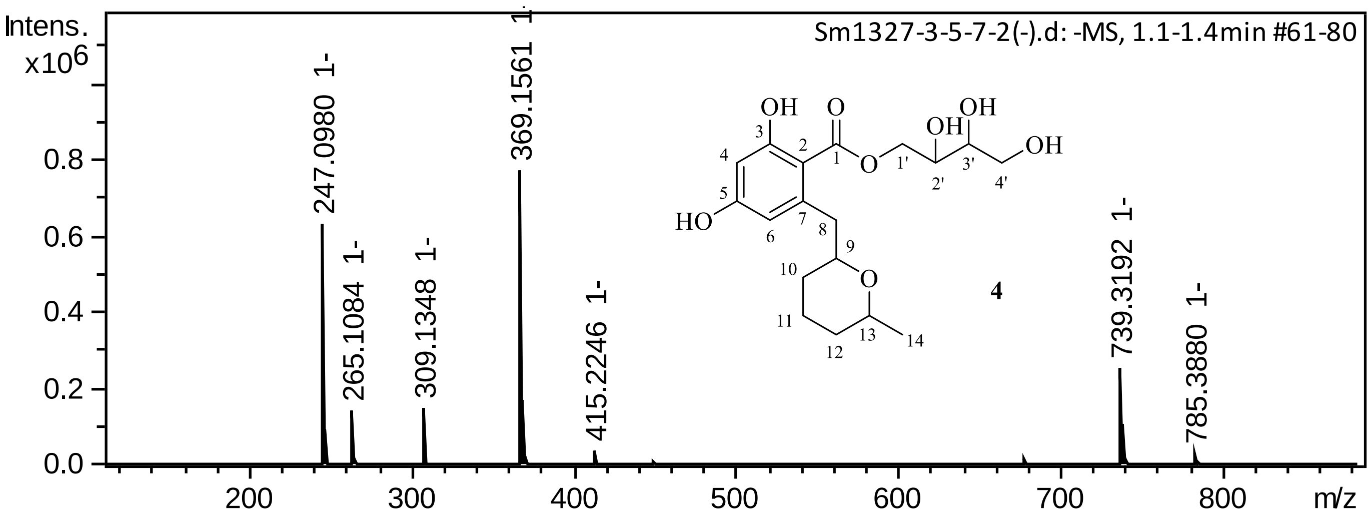

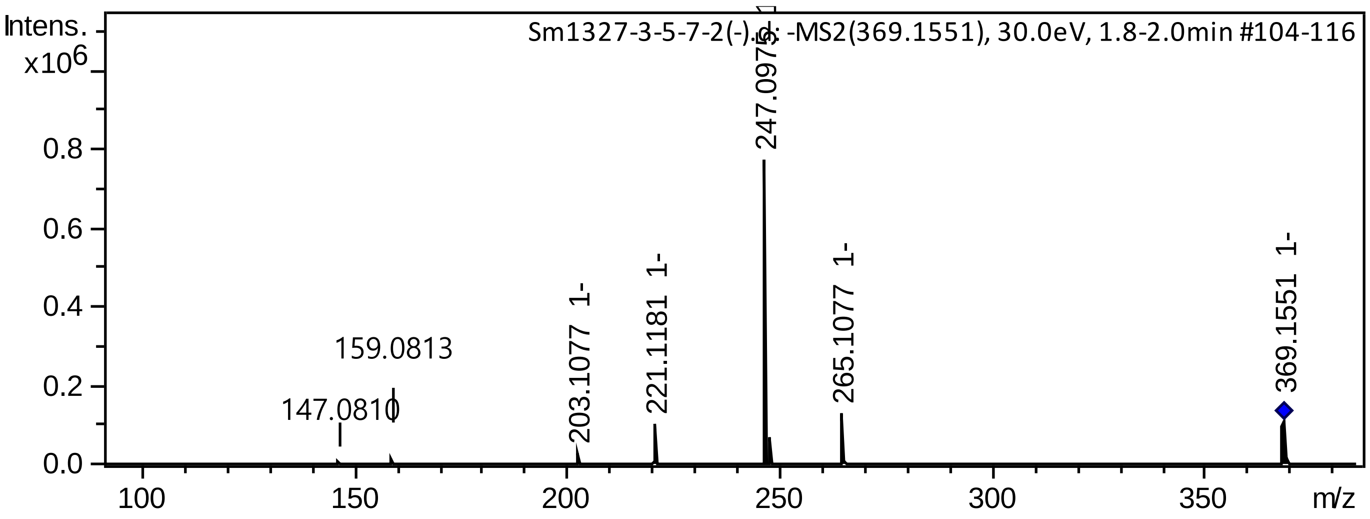

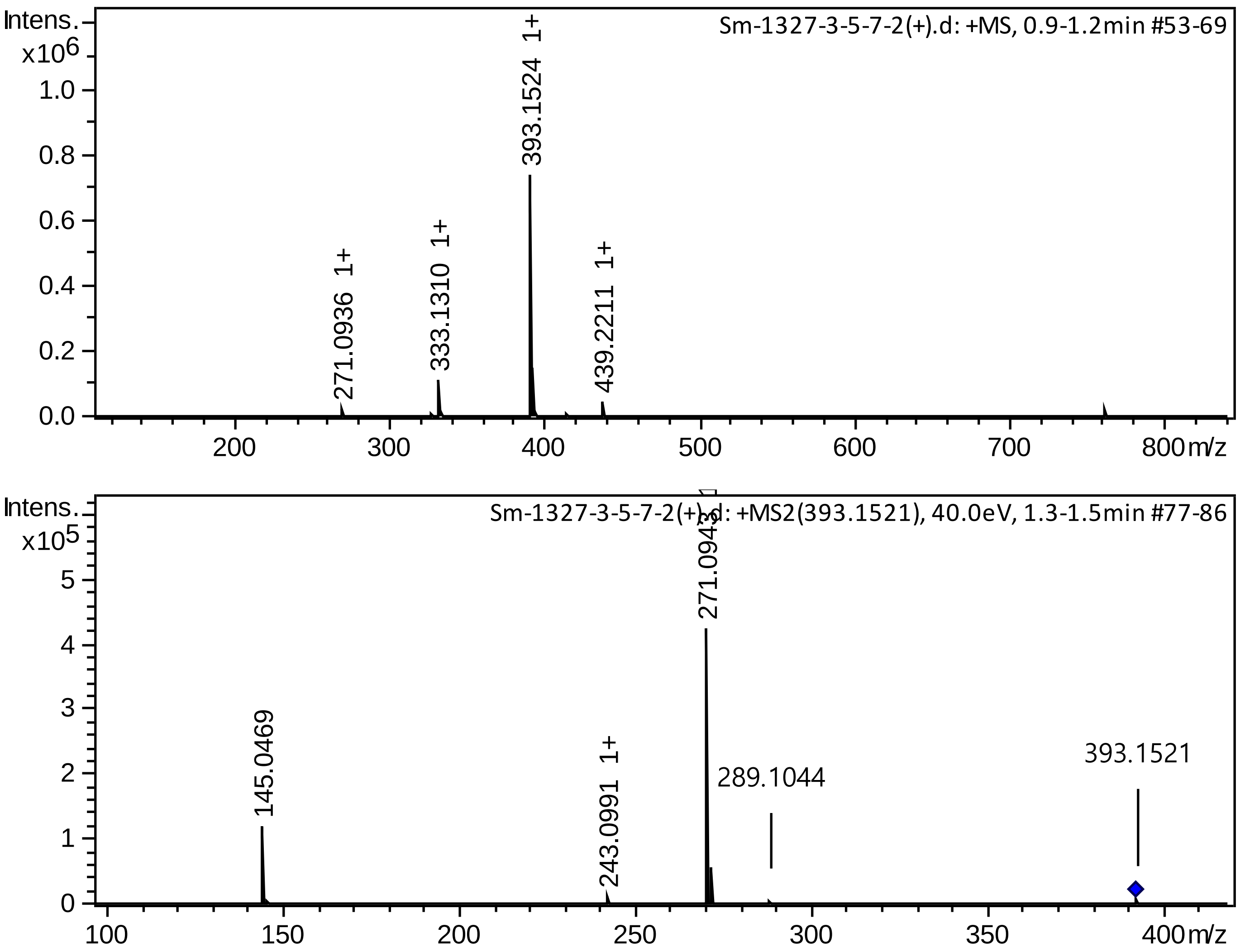

	meas	calc	Δ (ppm)
$[M-H]^-$	369,1561	369,1555	-1,7
$[M+Na]^+$	393,1524	393,152	-0,9

Figure S37. ^{1}H NMR spectrum of 6 measured at 700 MHz in acetone-d_6

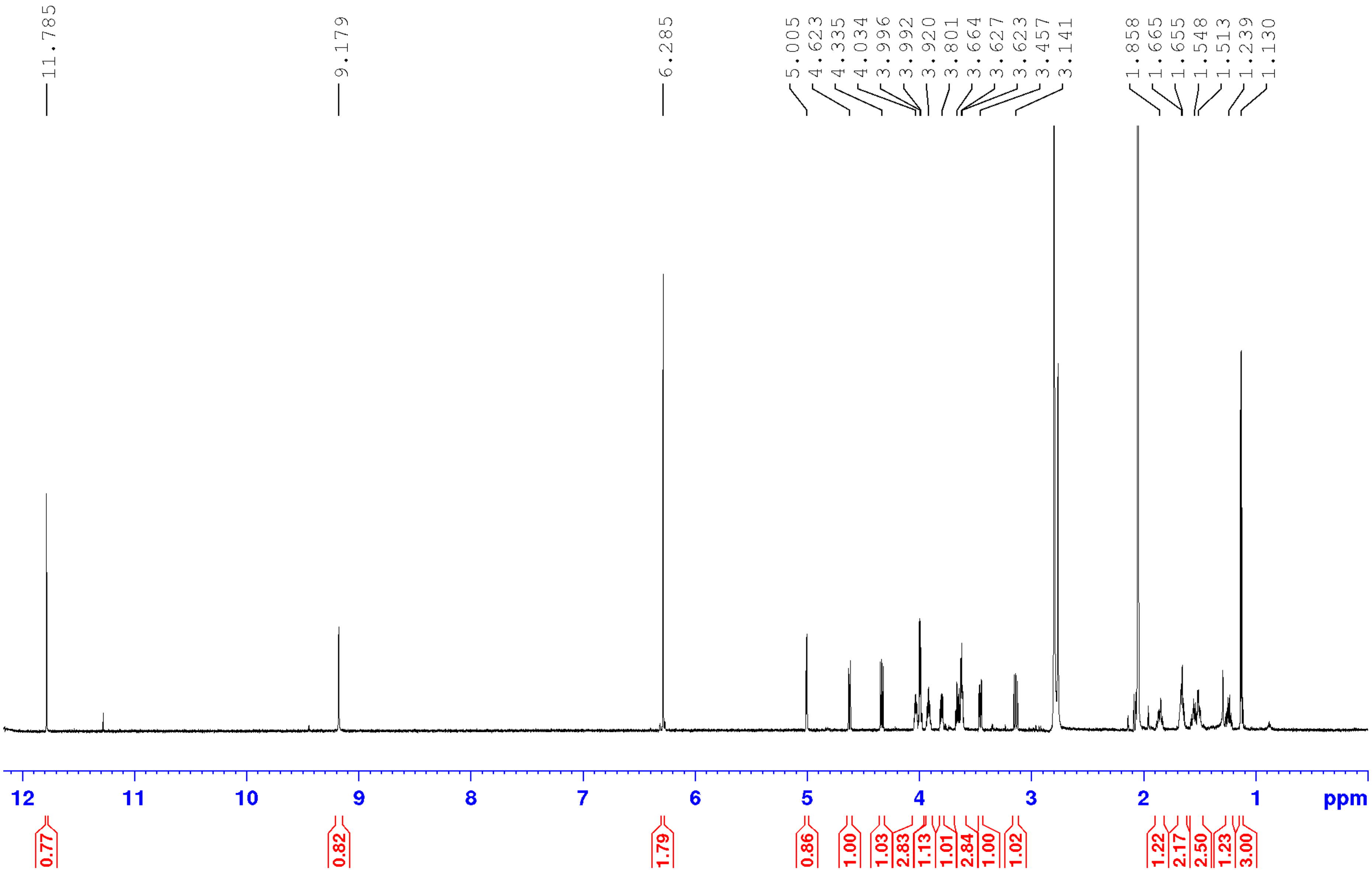

Figure S38. ^{13}C **NMR spectrum of 6 measured at 175 MHz in acetone-d_6**

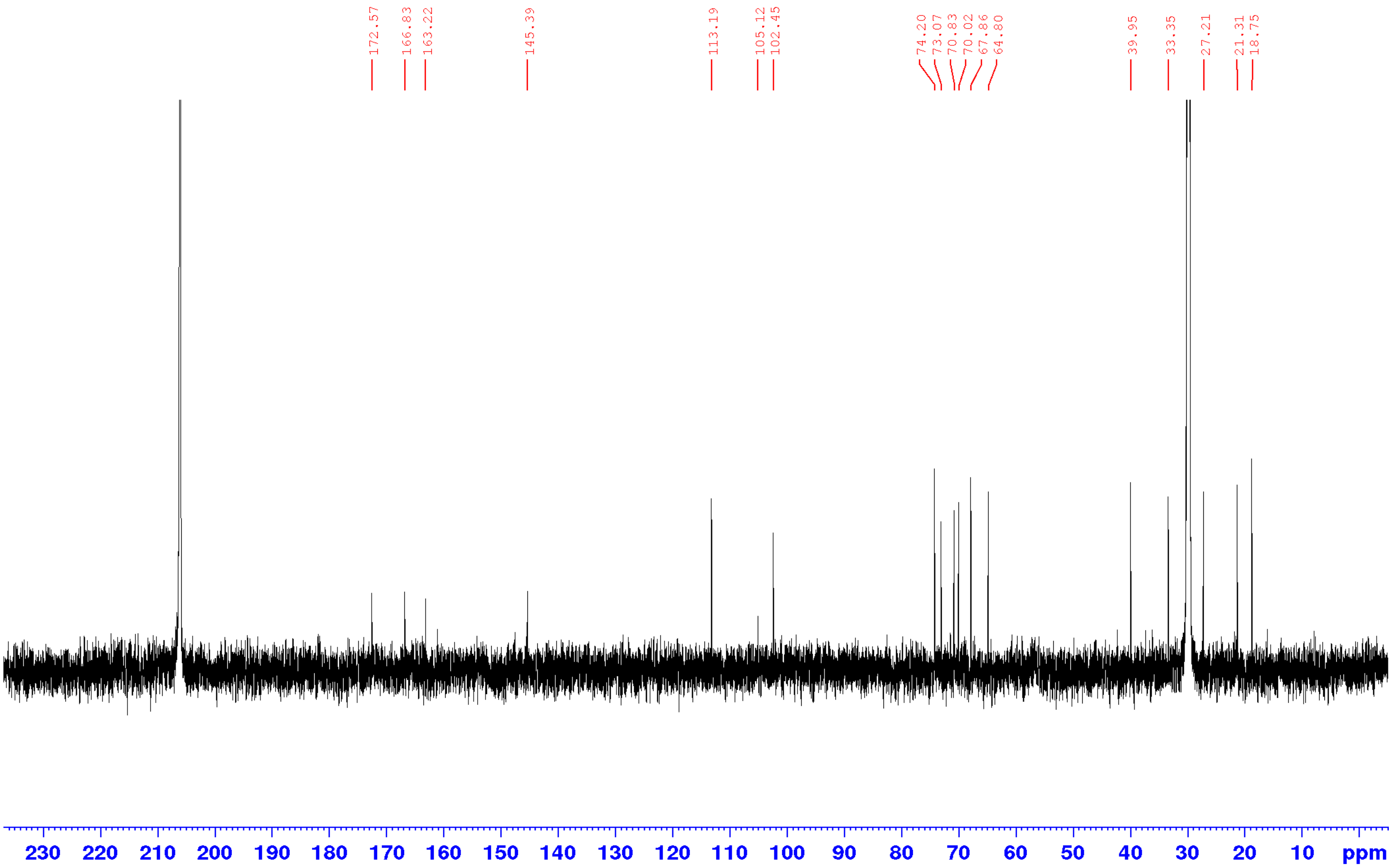

Figure S39. DEPT spectrum of 6 measured at 175 MHz in MeOD

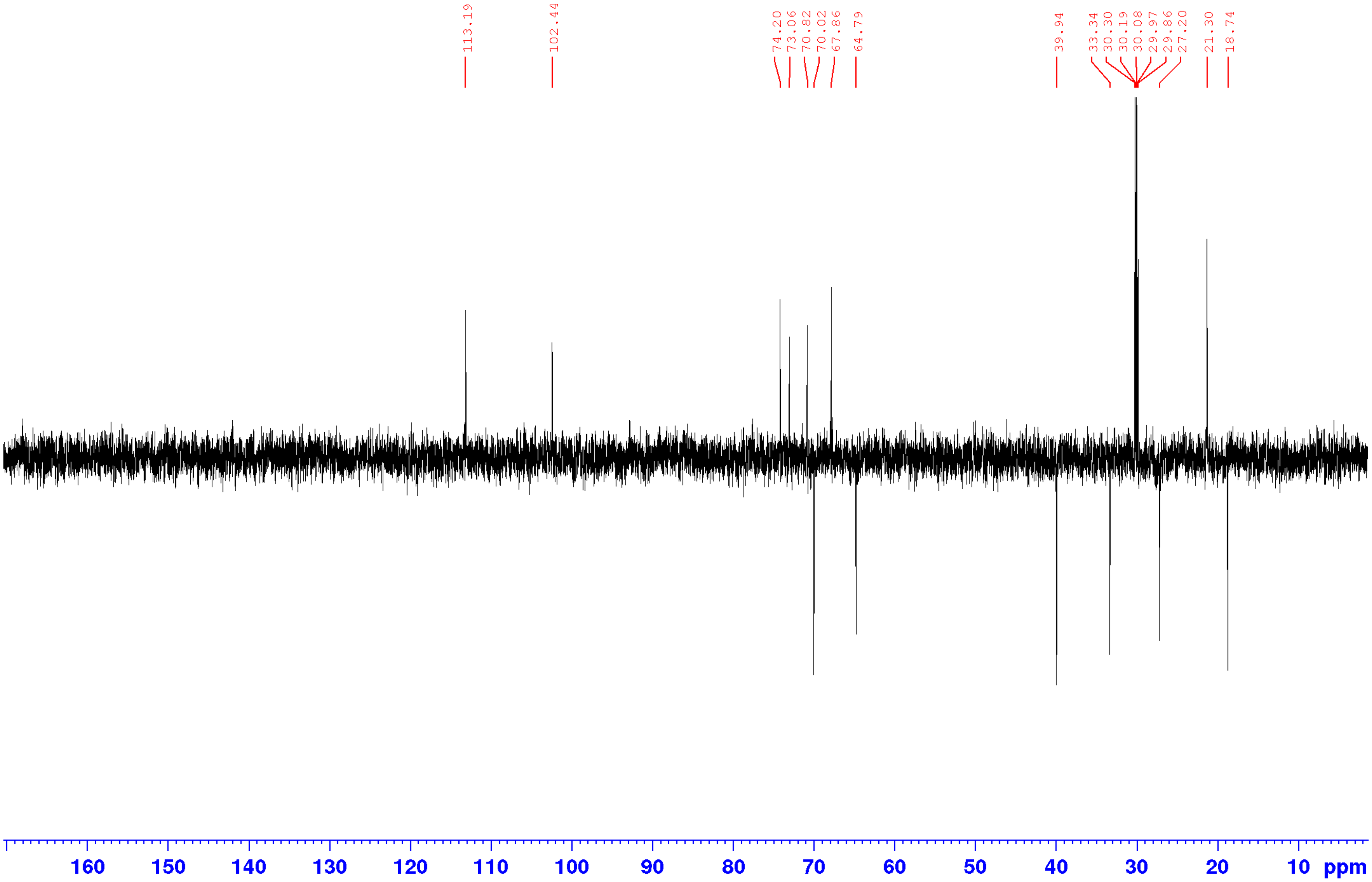

Figure S40. HSQC spectrum of 6 measured in acetone-d$_6$

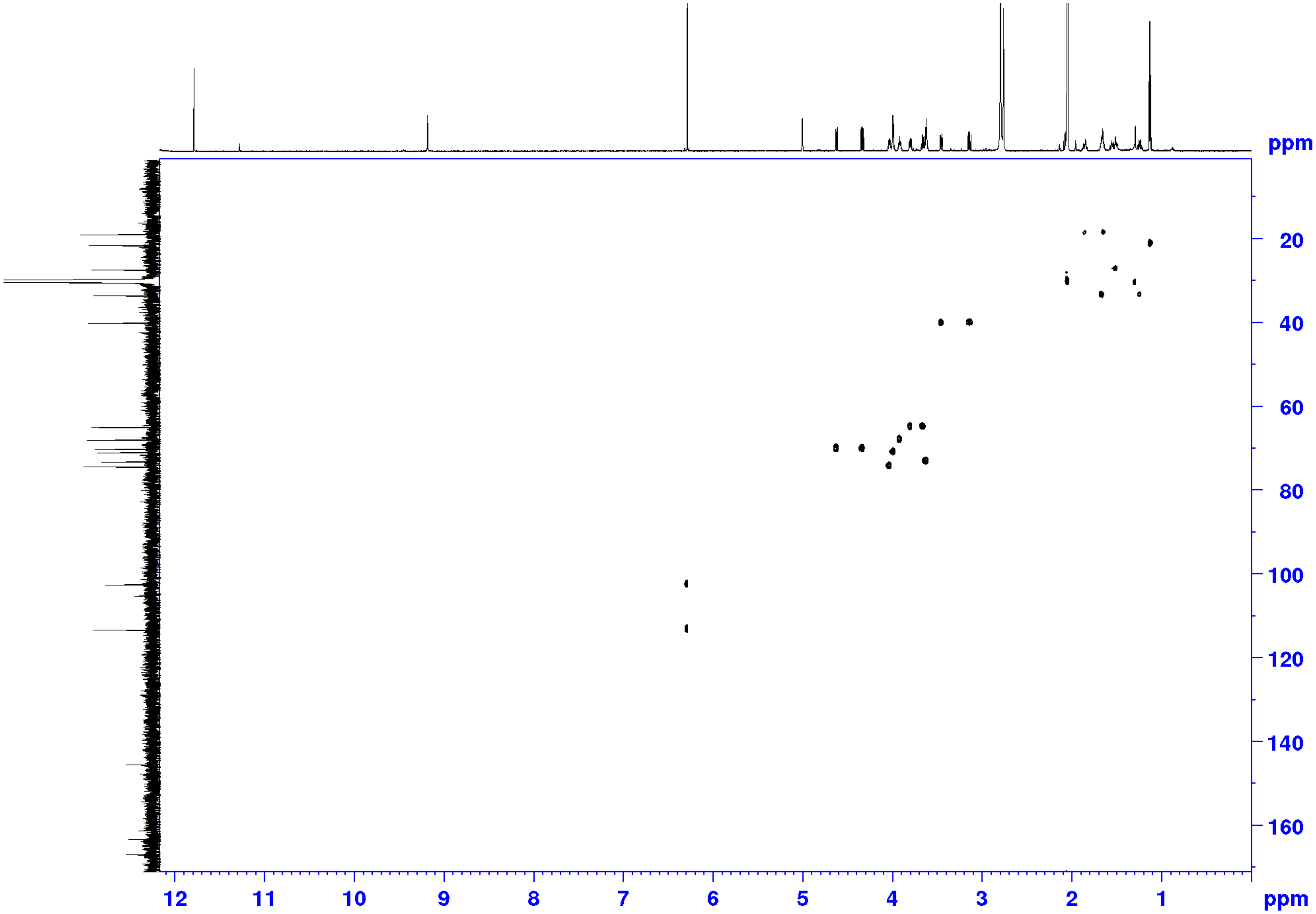

Figure S41. COSY spectrum of 6 measured in acetone-d$_6$

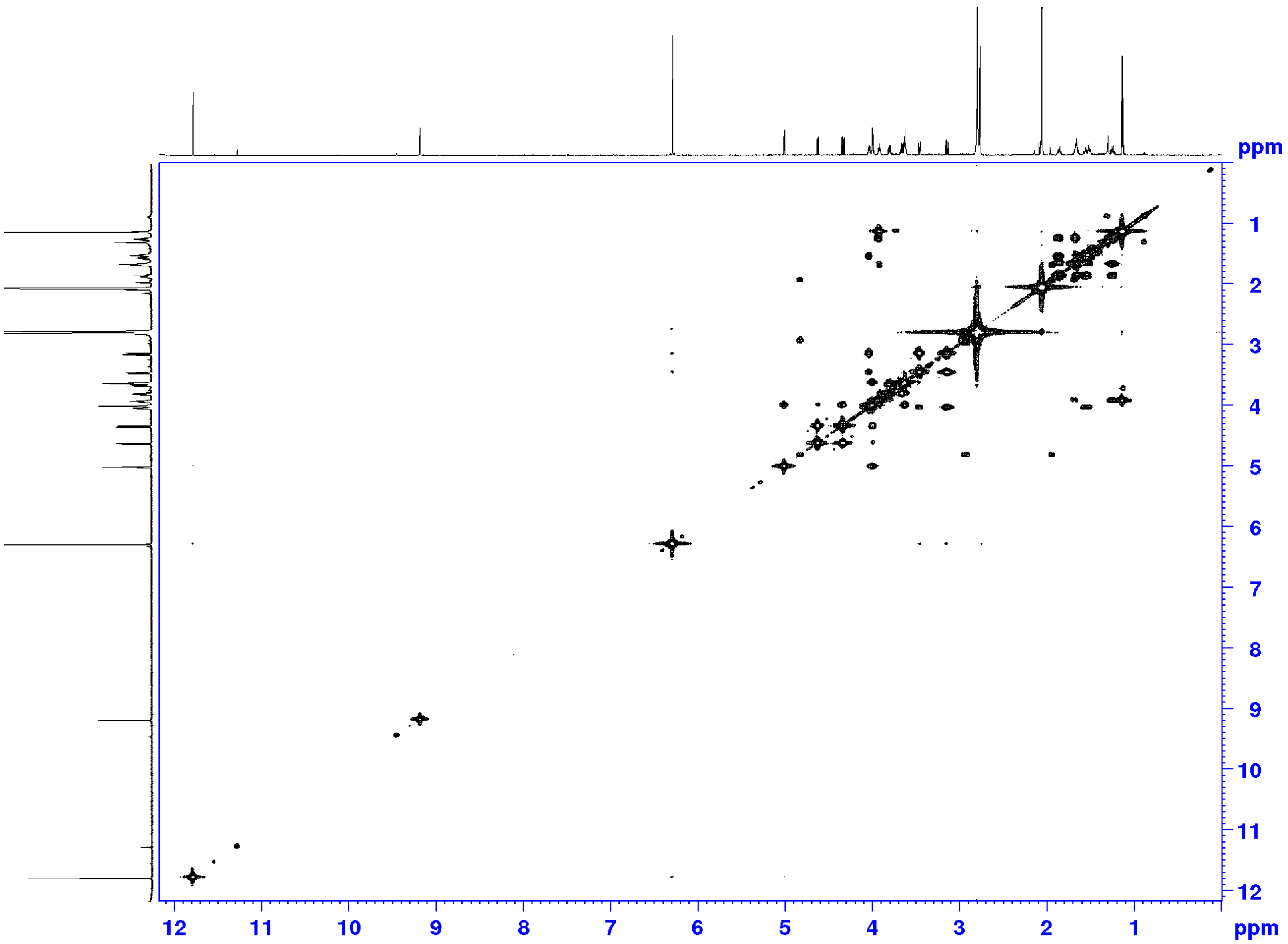

Figure S42. HMBC spectrum of 6 measured in acetone-d_6

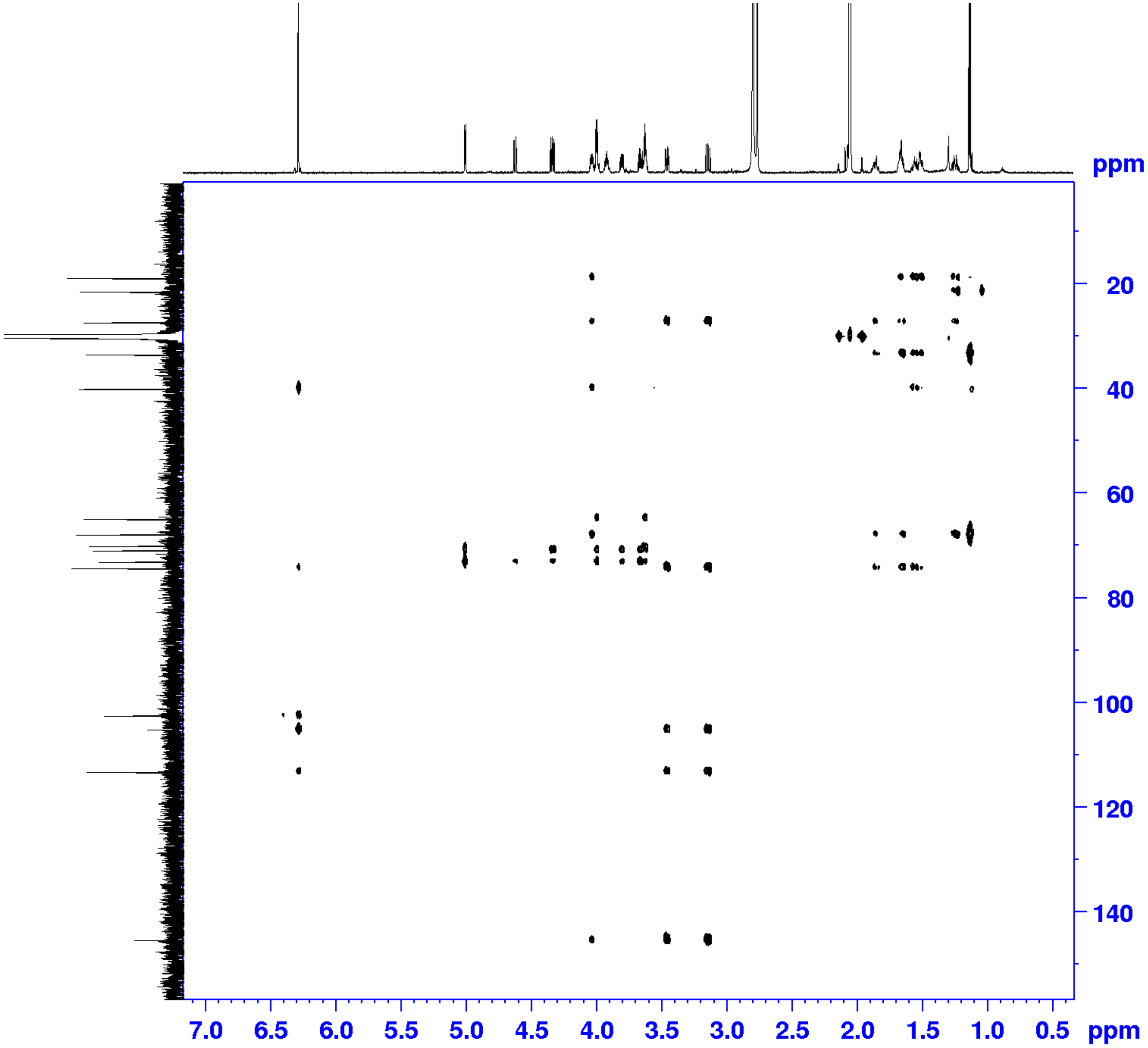

Figure S43. HRESIMS for 6a

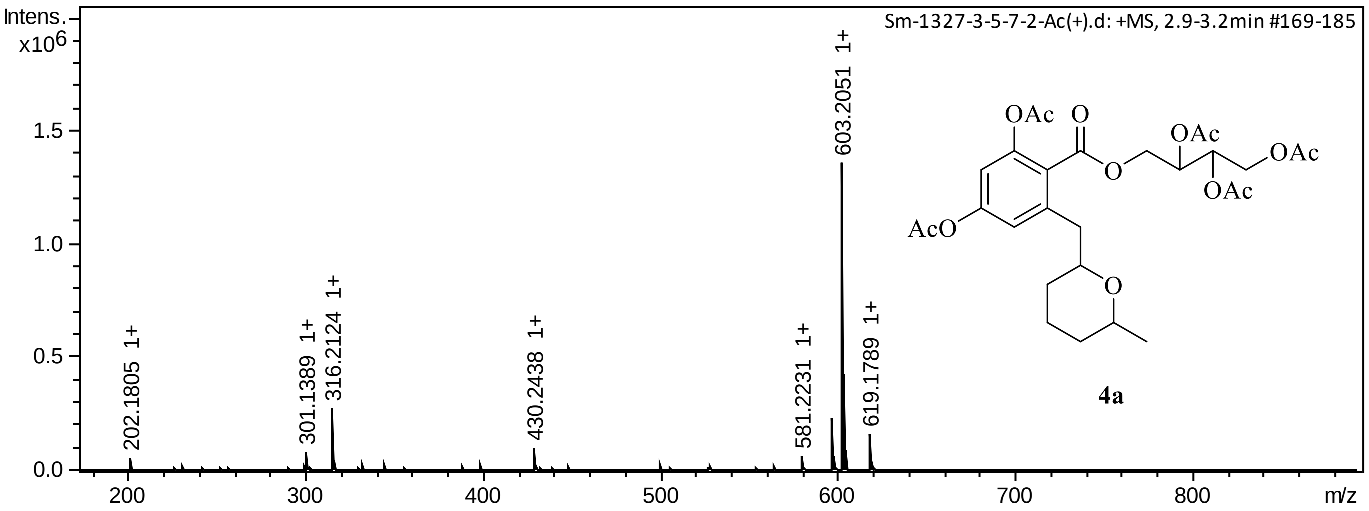

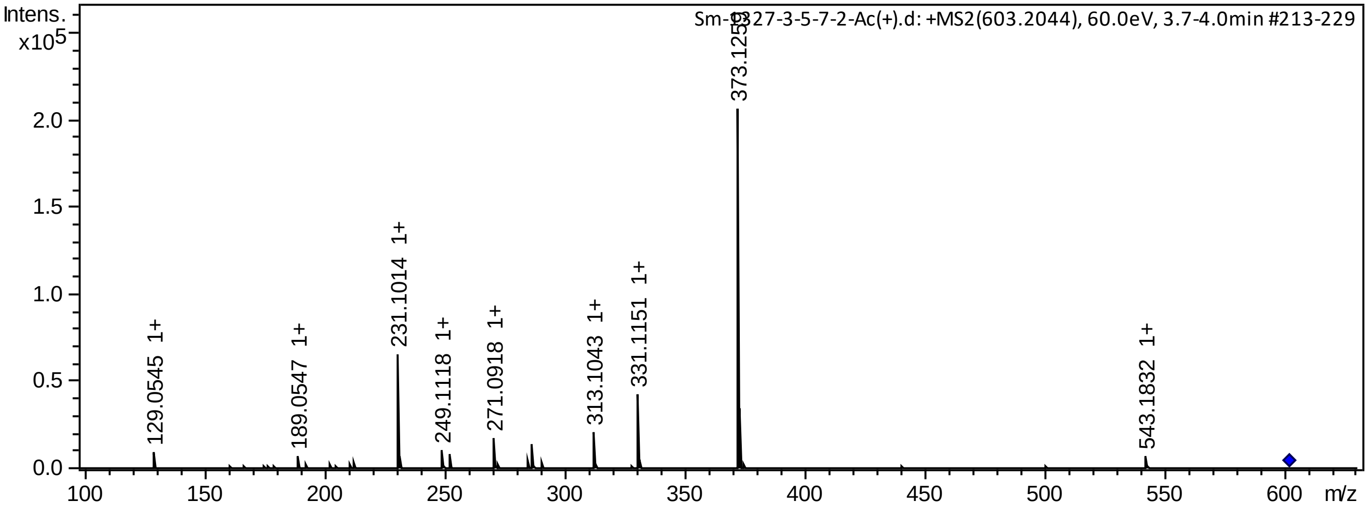

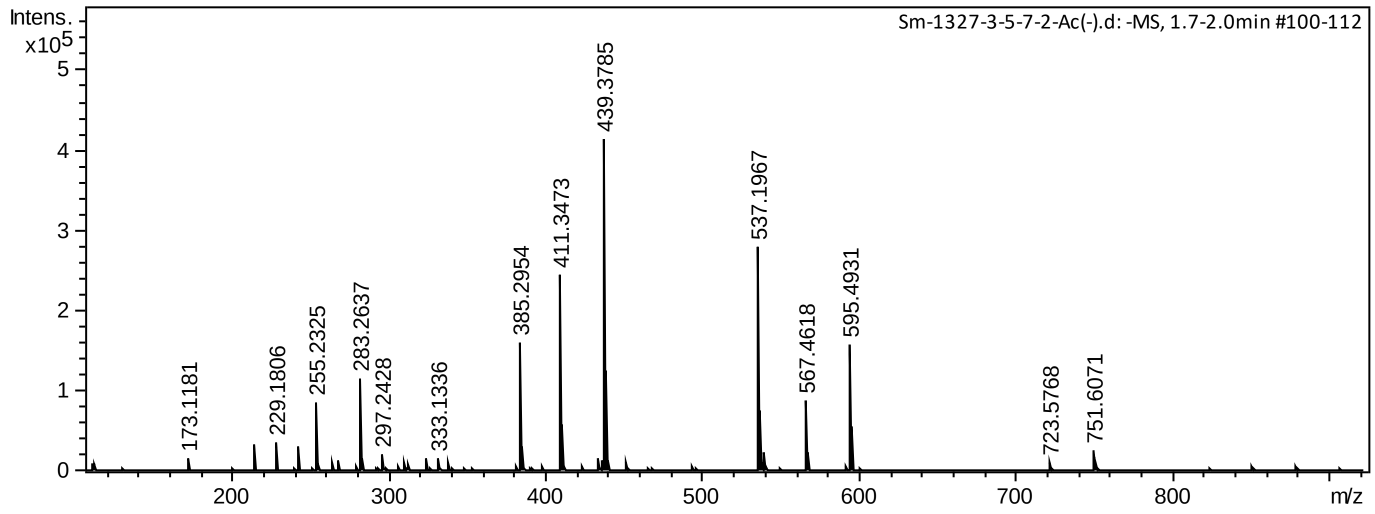

	meas	calc	Δ (ppm)
$[M+Na]^+$	603,2051	603,2048	-0,4

Meas. m/z	Ion Formula	m/z	err [ppm]	mSigma	rdb	e¯ Conf	N-Rule
129,0545	C6H9O3	129,0546	1	4,7	3	even	ok
189,0547	C11H9O3	189,0546	-0,2	11,2	8	even	ok
231,1014	C14H15O3	231,1016	0,8	28,9	8	even	ok
249,1118	C14H17O4	249,1121	1,2	26,4	7	even	ok
271,0918	C14H16NaO4	271,0941	8,4	27,6	7	even	ok
285,1092	C15H18NaO4	285,1097	1,9	16	7	even	ok
287,1251	C15H20NaO4	287,1254	1,1	29,4	6	even	ok
313,1043	C16H18NaO5	313,1046	1,1	27,9	8	even	ok
331,1151	C16H20NaO6	331,1152	0,3	30,4	7	even	ok
373,1259	C18H22NaO7	373,1258	-0,3	20,4	8	even	ok
441,1515	C22H26NaO8	441,152	1,1	30	10	even	ok

485,1786	C24H30NaO9	485,1782	-0,9	n.a.	10	even	ok
501,1725	C24H30NaO10	501,1731	1,2	28,2	10	even	ok
543,1832	C26H32NaO11	543,1837	0,9	30,8	11	even	ok
603,2044	C28H36NaO13	603,2048	0,7	39,4	11	even	ok
253,0681	C10H14NaO6	253,0683	0,6	7,7	4	even	ok

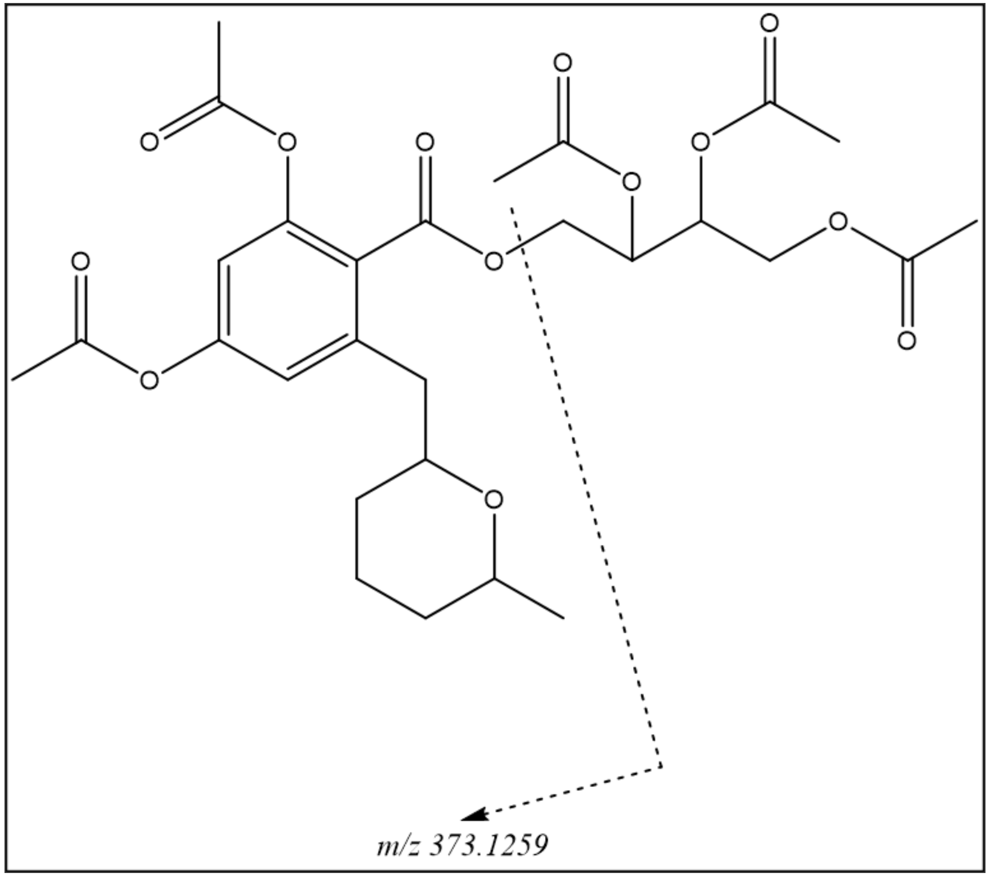

Figure S44. ^{1}H NMR spectrum of **6a** measured at 700 MHz in acetone-d6

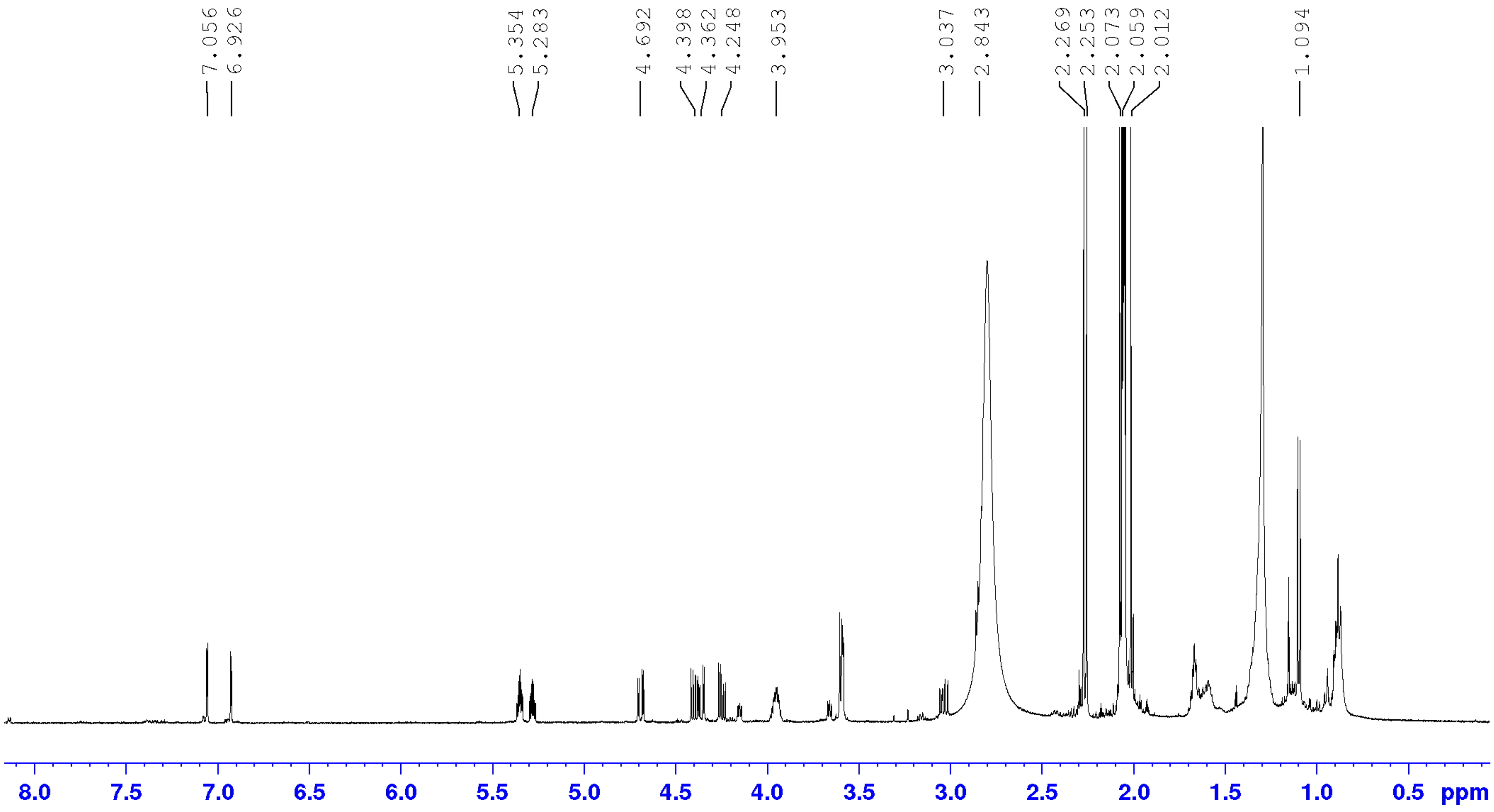

Figure S45. ^{13}C **NMR spectrum of 6a measured at 175 MHz in acetone-d6**

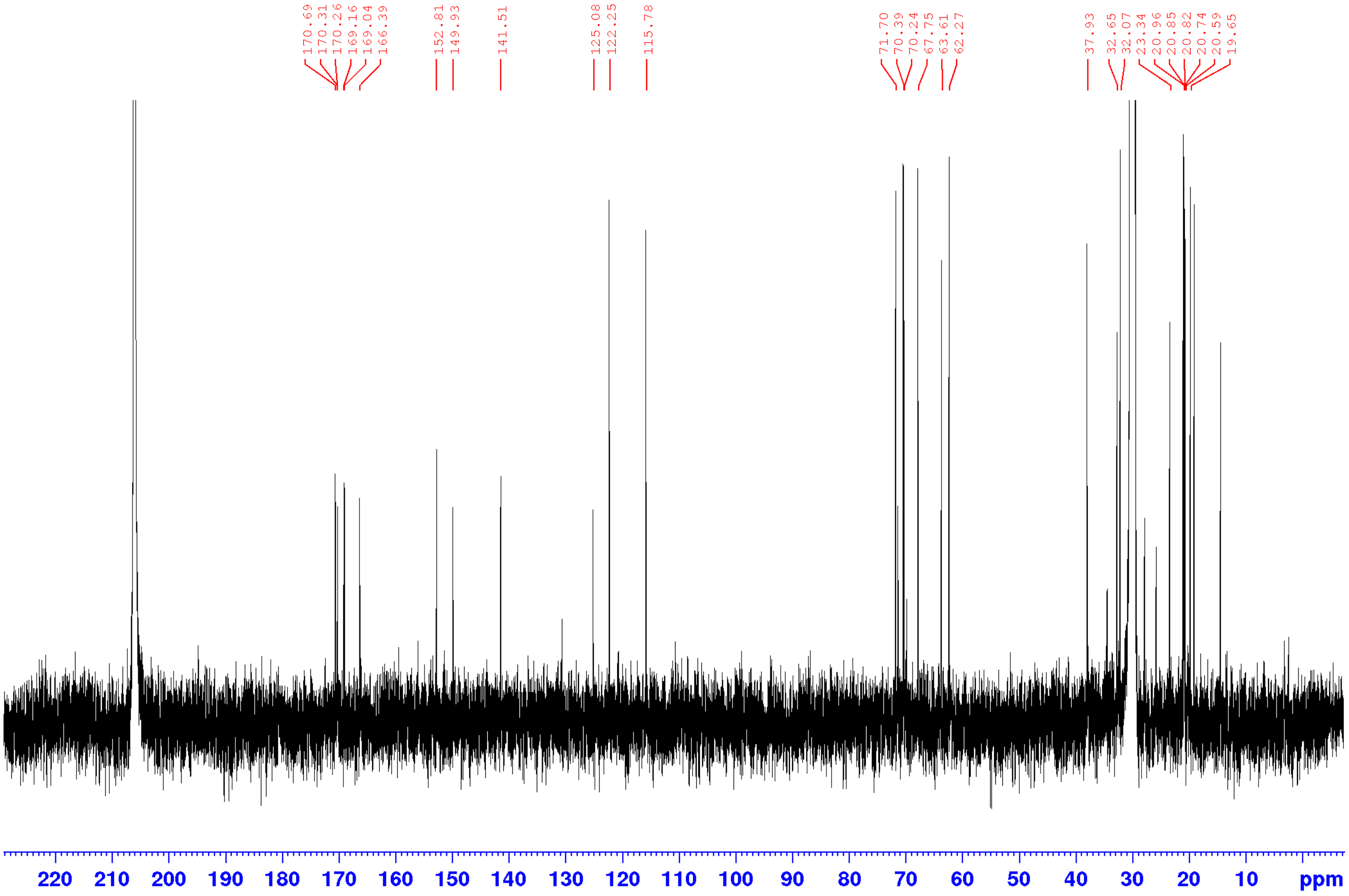

Figure S46. HSQC spectrum of 6a measured in acetone-d6

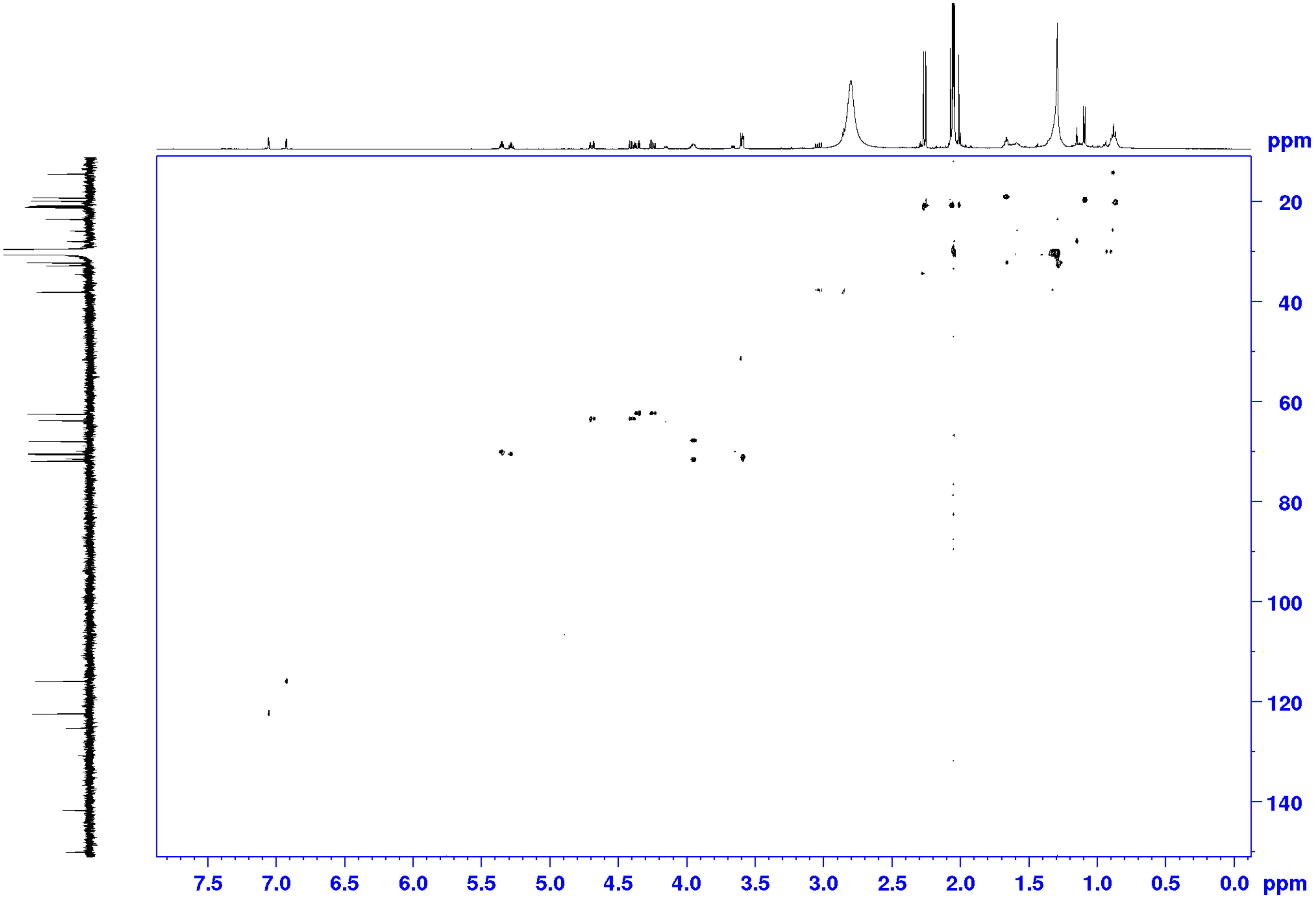

Figure S47. COSY spectrum of 6a measured in acetone-d6

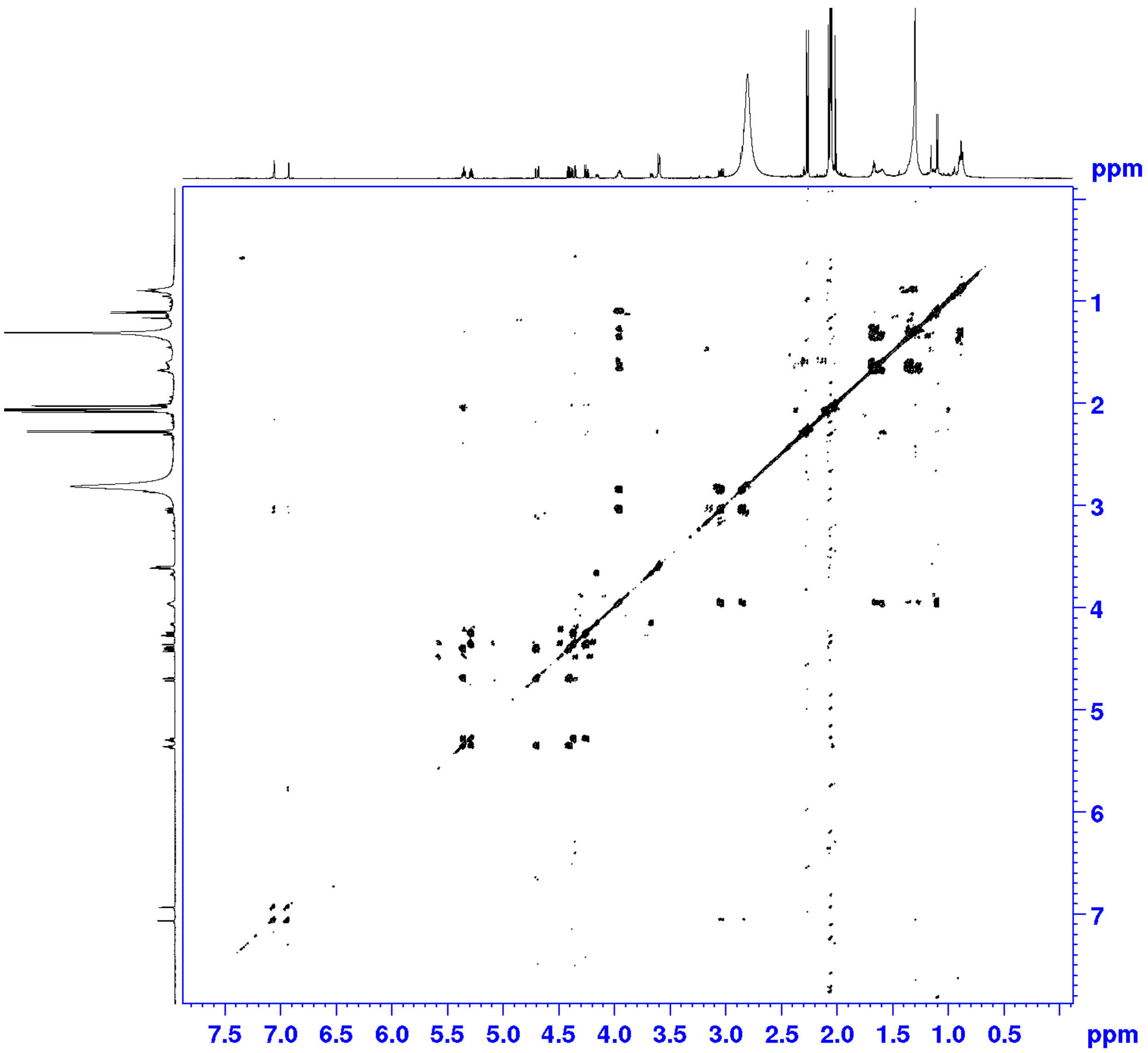

Figure S48. UV and CD data for 1

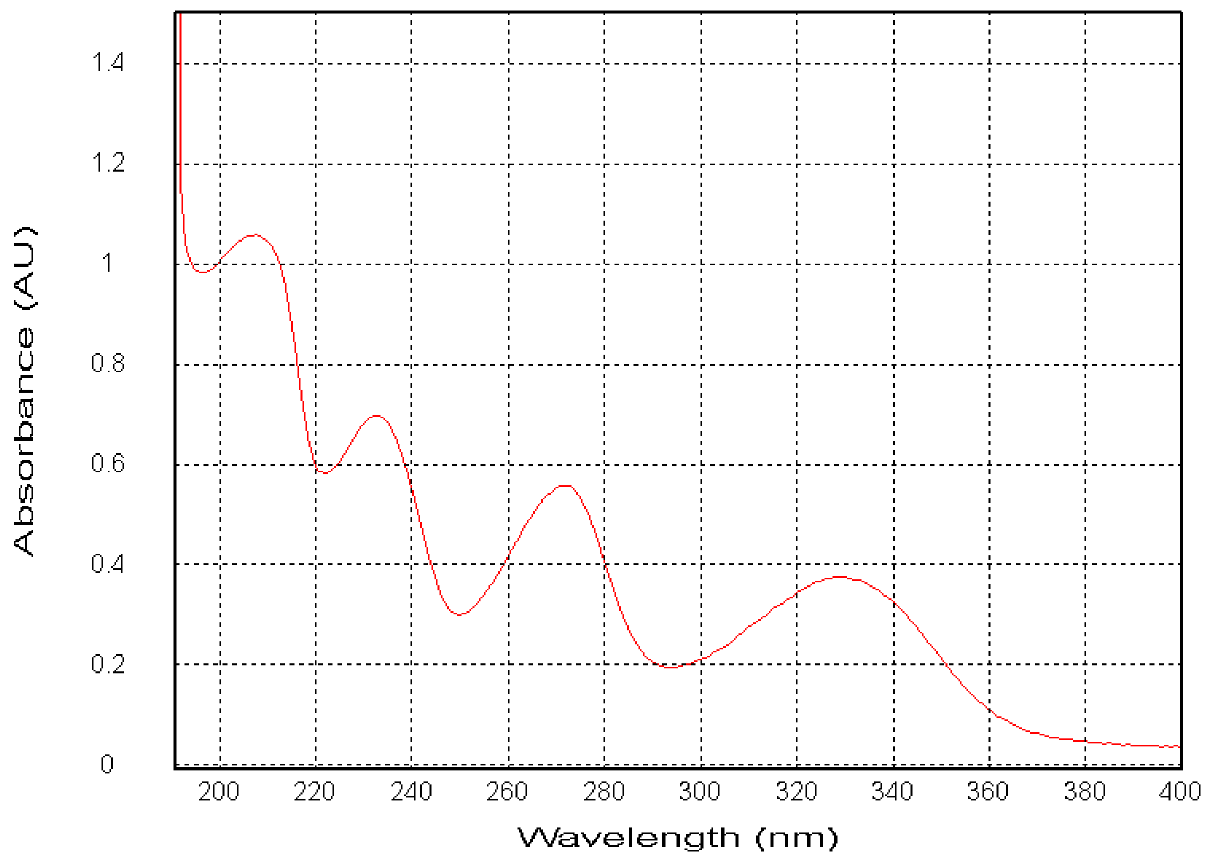

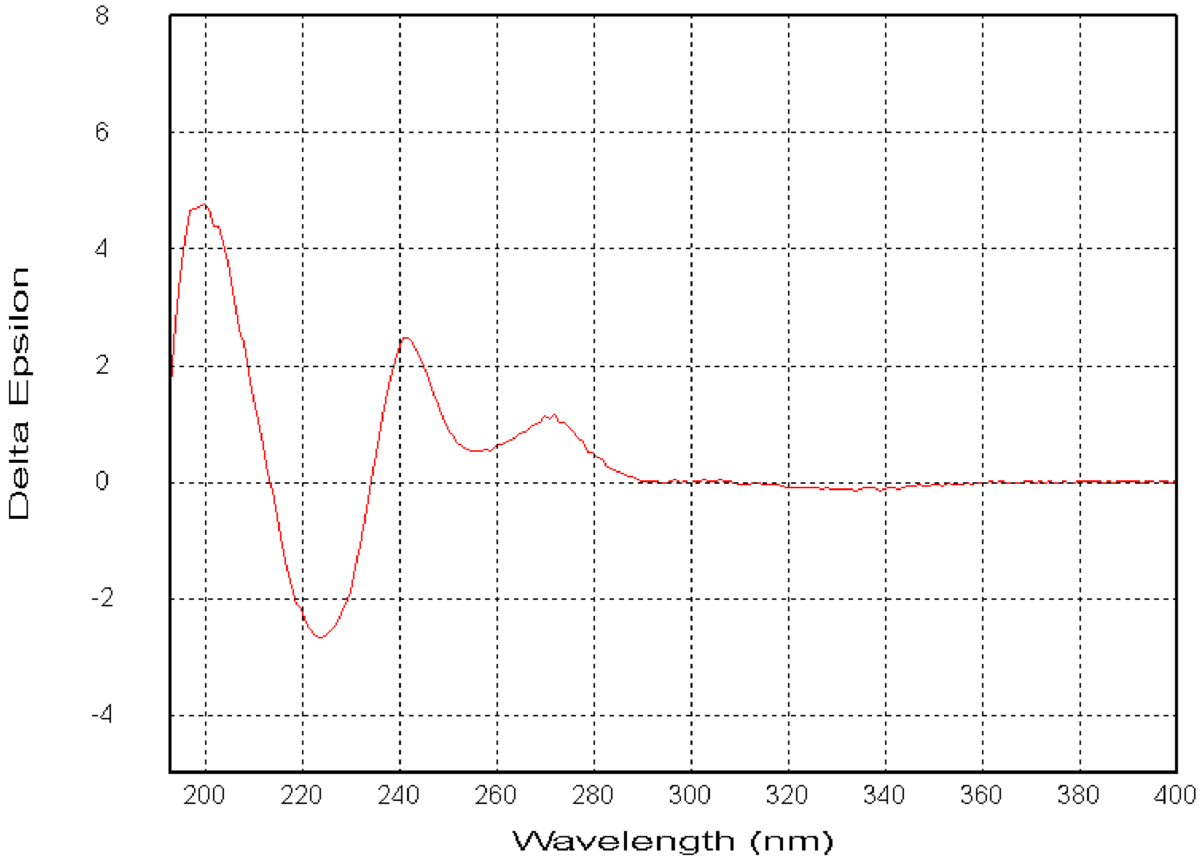

Delta Epsilon
8
6
4
2
0
-2
-4
200
220
240
260
280
300
320
340
360
380
400
Wavelength (nm)

Figure S49. UV and CD data for 3

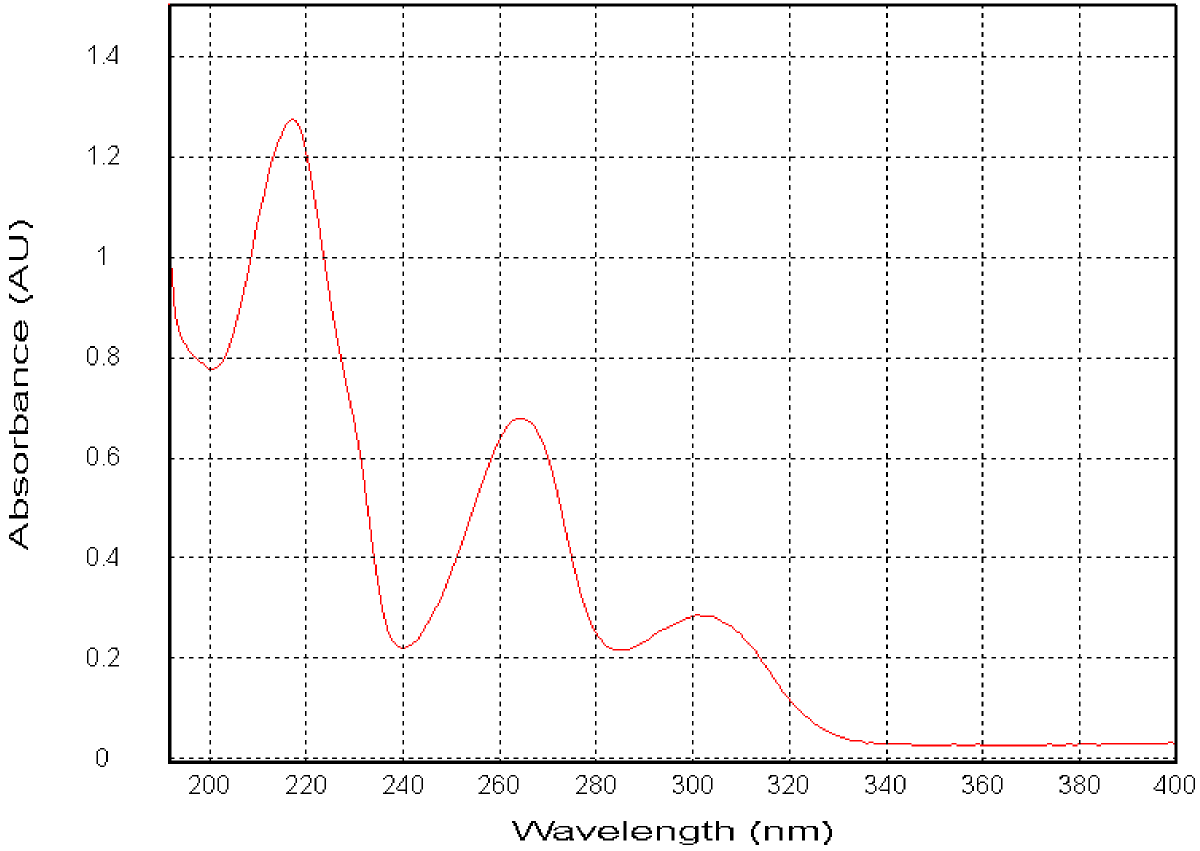

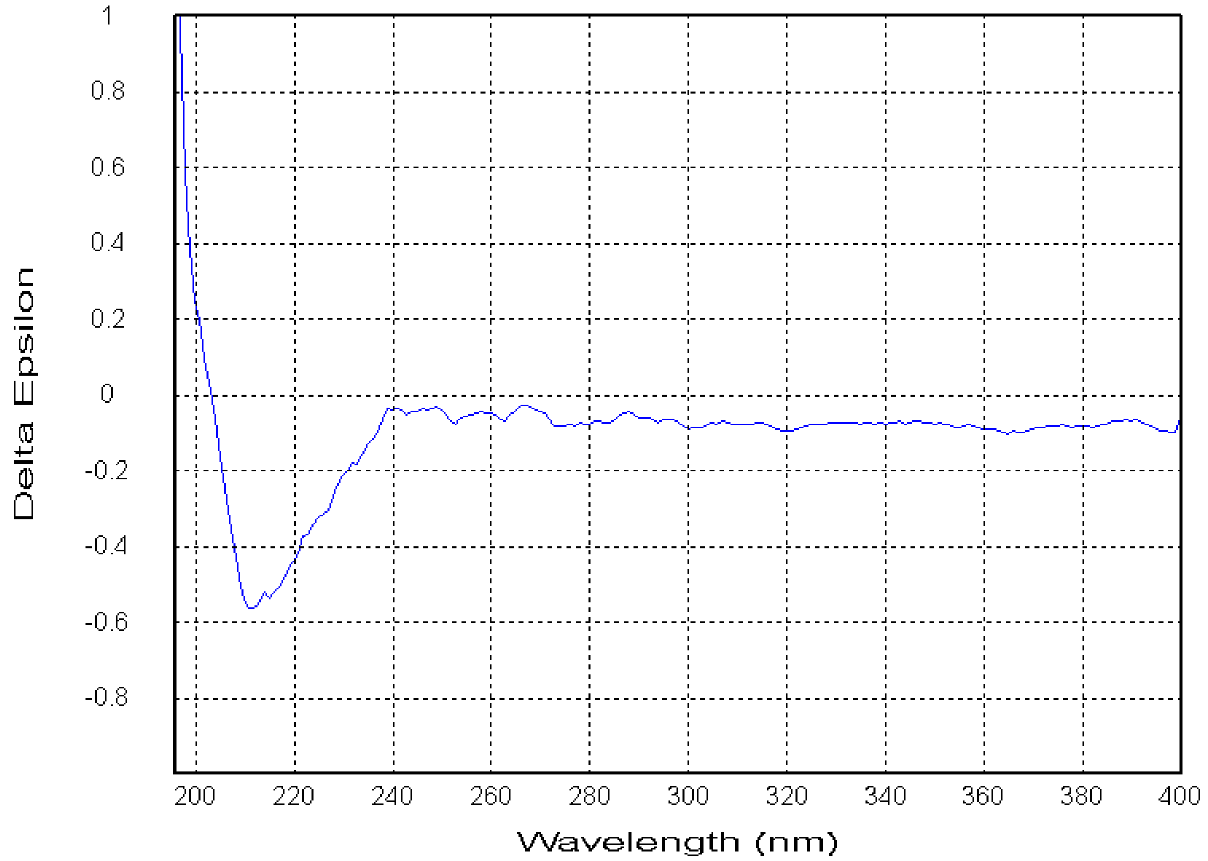

Delta Epsilon
1
0.8
0.6
0.4
0.2
0
-0.2
-0.4
-0.6
-0.8
200
220
240
260
280
300
320
340
360
380
400
Wavelength (nm)

Figure S50. UV and CD data for 4

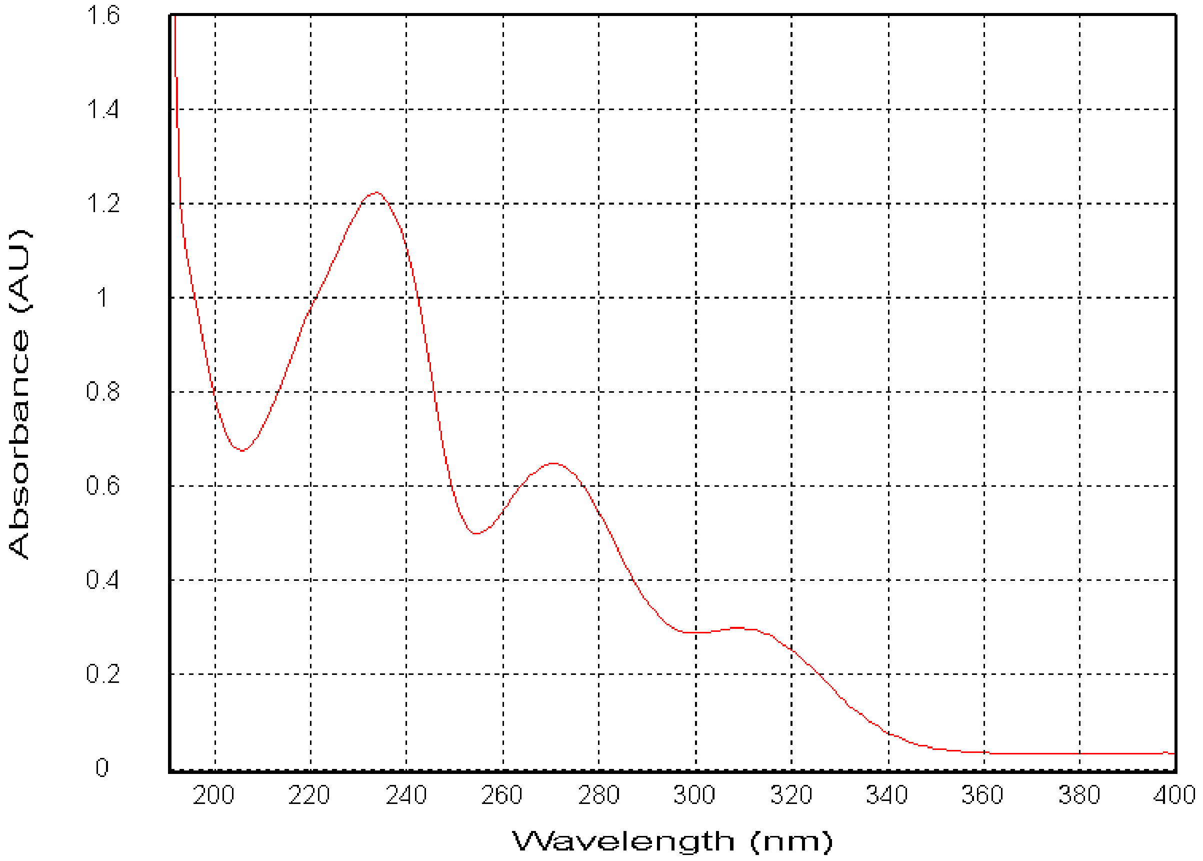

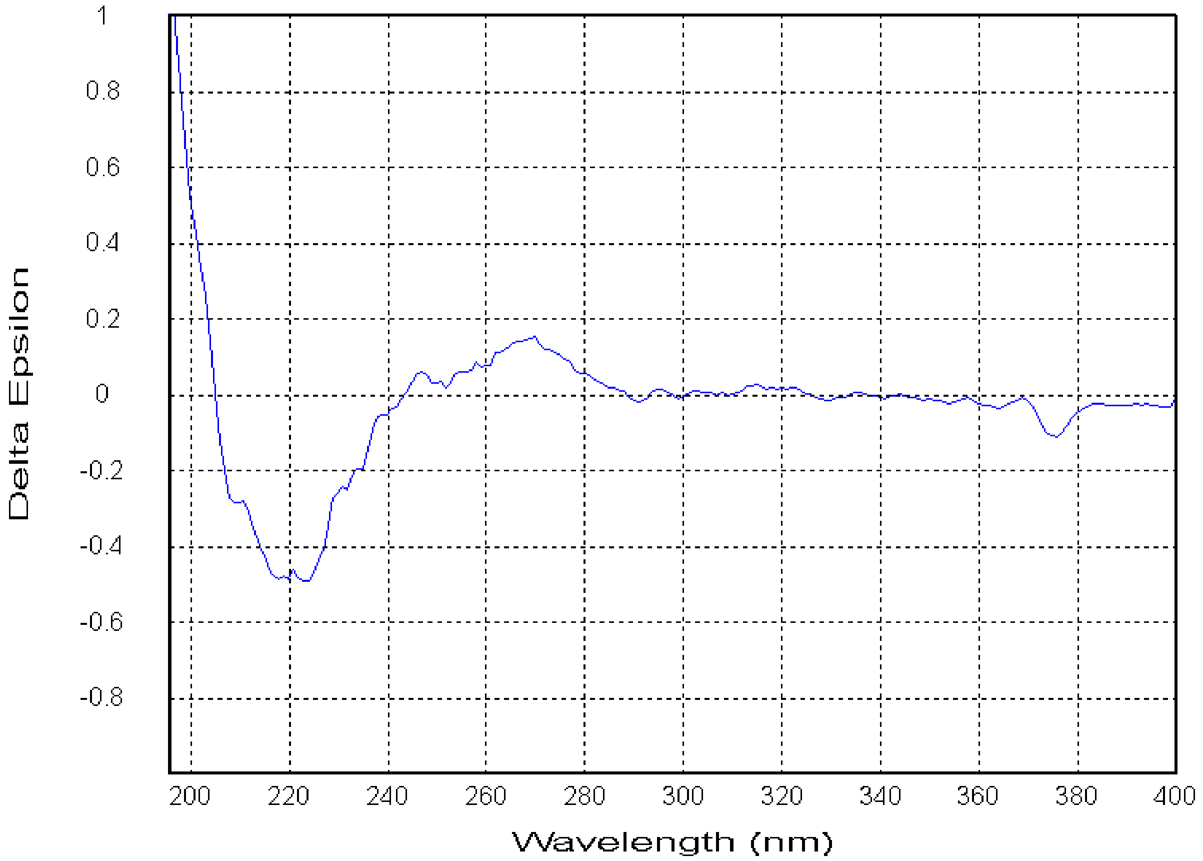

Delta Epsilon
1
0.8
0.6
0.4
0.2
0
-0.2
-0.4
-0.6
-0.8
200
220
240
260
280
300
320
340
360
380
400
Wavelength (nm)

Figure S51. UV and CD data for 5

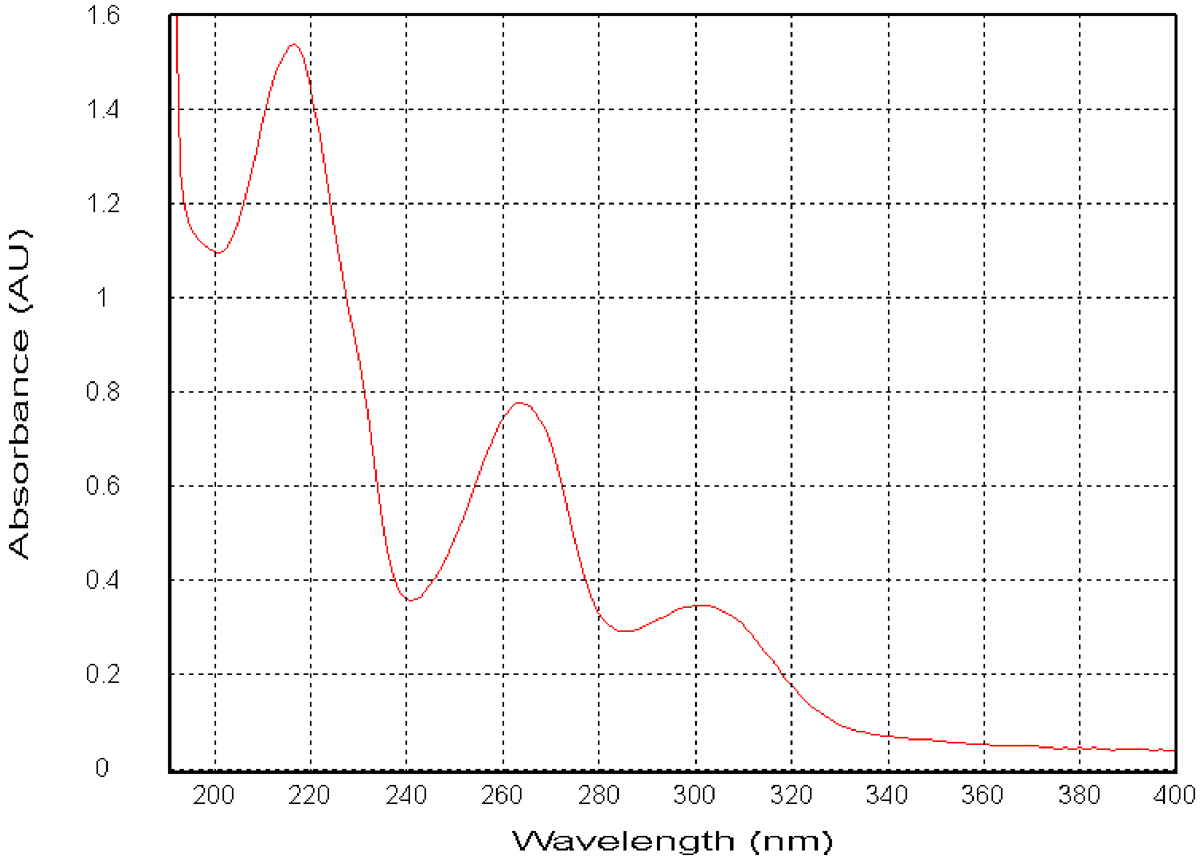

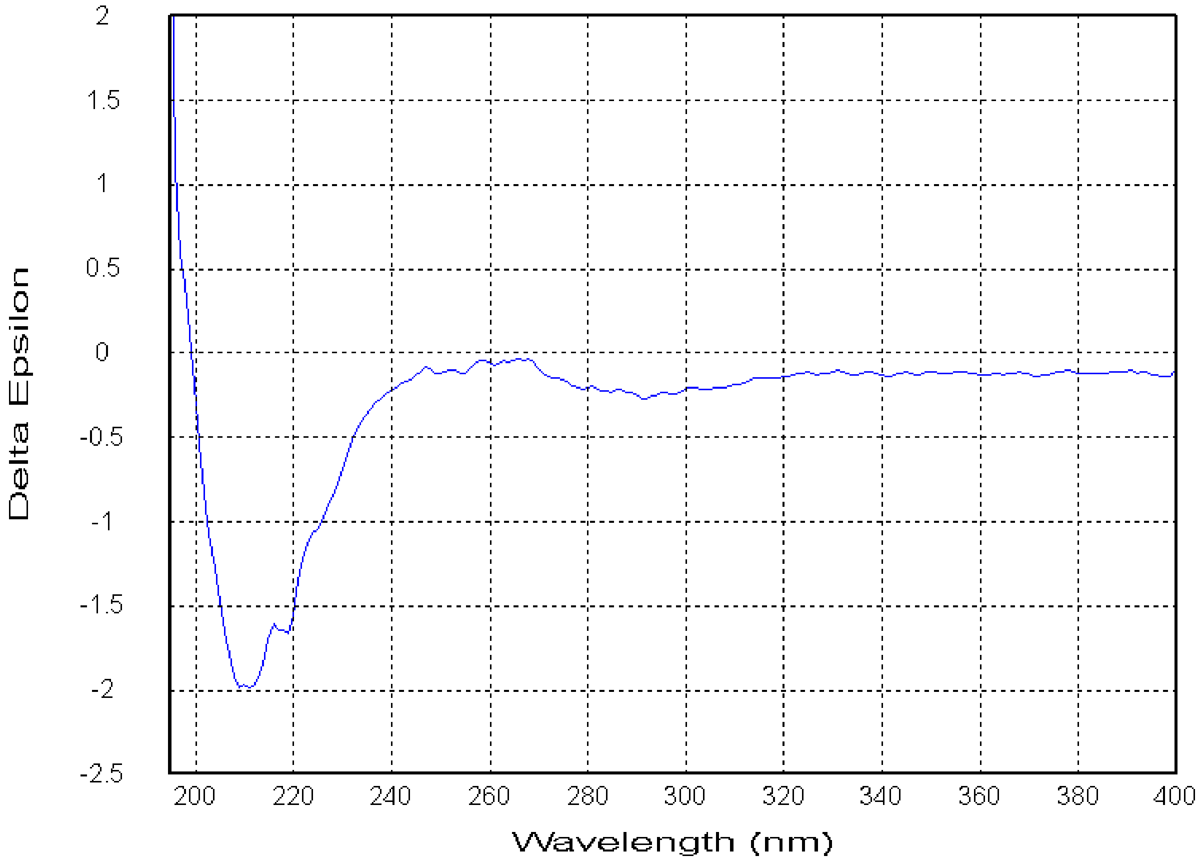

Delta Epsilon
2
1.5
1
0.5
0
-0.5
-1
-1.5
-2
-2.5
200
220
240
260
280
300
320
340
360
380
400
Wavelength (nm)

Figure S52. UV and CD data for 6

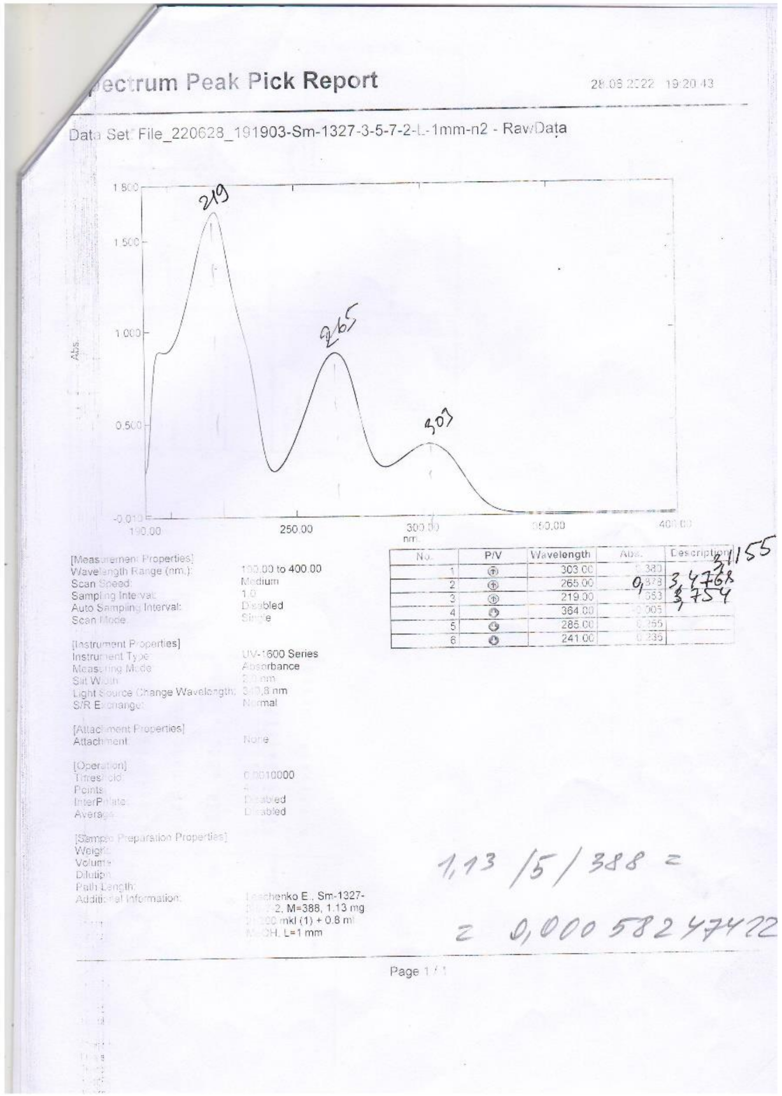

[illegible]pectrum Peak Pick Report

28.06.2022 19:20:43

Data Set: File_220628_191903-Sm-1327-3-5-7-2-L-1mm-n2 - RawData

No.	P/V	Wavelength	Abs.	Description
1	⊕	303.00	0.380	
2	⊕	265.00	0.878	
3	⊕	219.00	[illegible].653	
4	⊖	364.00	-0.005	
5	⊖	285.00	0.265	
6	⊖	241.00	0.235	

Handwritten: 3,1155; 0,878 3,4768; 3,754

[Measurement Properties]
Wavelength Range (nm.): 190.00 to 400.00
Scan Speed: Medium
Sampling Interval: 1.0
Auto Sampling Interval: Disabled
Scan Mode: Single

[Instrument Properties]
Instrument Type: UV-1600 Series
Measuring Mode: Absorbance
Slit Width: 2.0 nm
Light Source Change Wavelength: 3[illegible],8 nm
S/R Exchange: Normal

[Attachment Properties]
Attachment: None

[Operation]
Threshold: 0.0010000
Points: [illegible]
InterPolate: Disabled
Average: Disabled

[Sample Preparation Properties]
Weight:
Volume:
Dilution:
Path Length:
Additional Information: [illegible]chenko E., Sm-1327-[illegible]2, M=388, 1.13 mg [illegible] mkl (1) + 0.8 ml [illegible]OH, L=1 mm

Handwritten: 1,13 / 5 / 388 = = 0,00058247422

Page 1 / 1

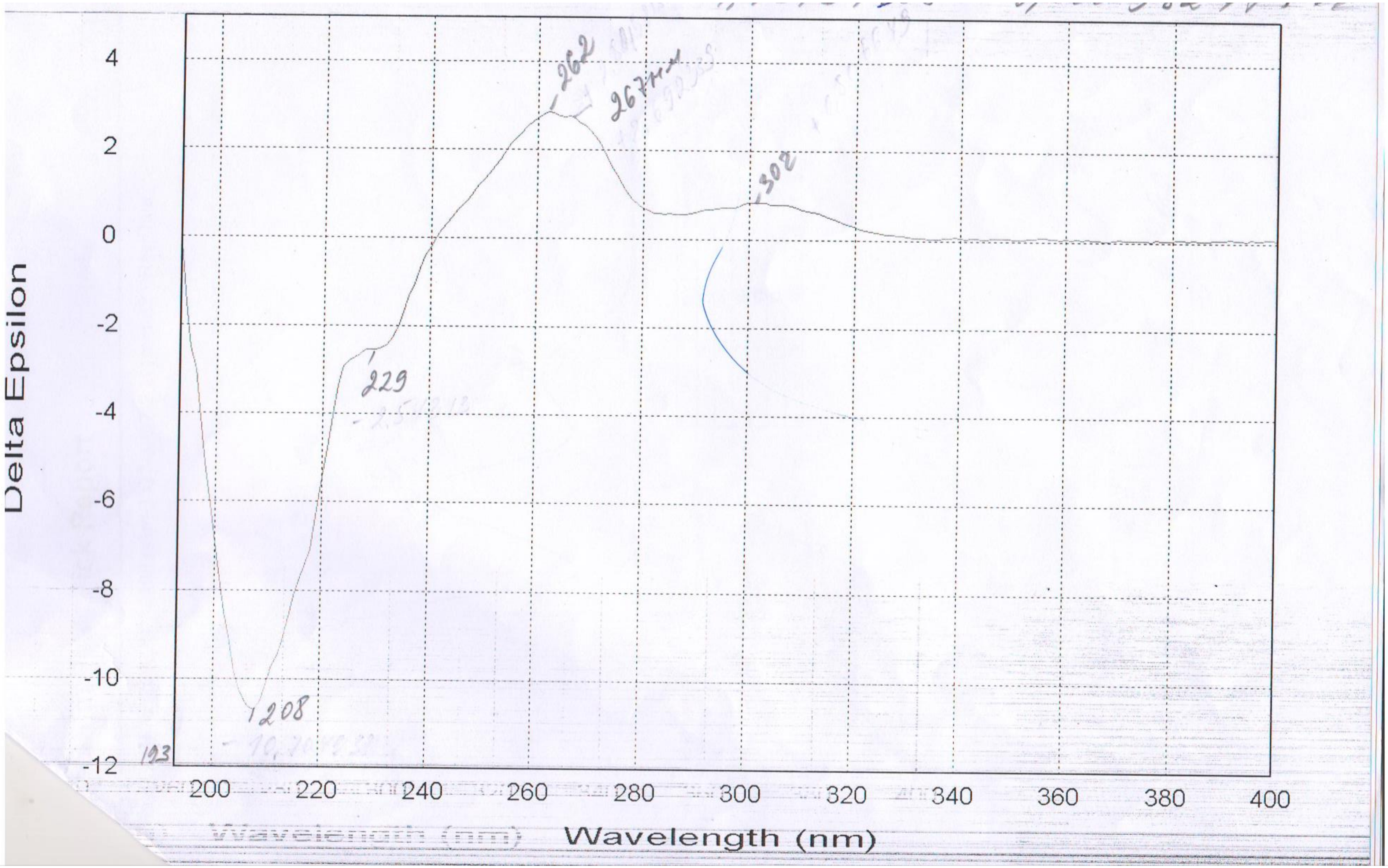
Delta Epsilon
Wavelength (nm)
4
2
0
-2
-4
-6
-8
-10
-12
200
220
240
260
280
300
320
340
360
380
400
262
267
302
229
208
193

Figure S53. 1H NMR spectrum for MTPA-2a (S)

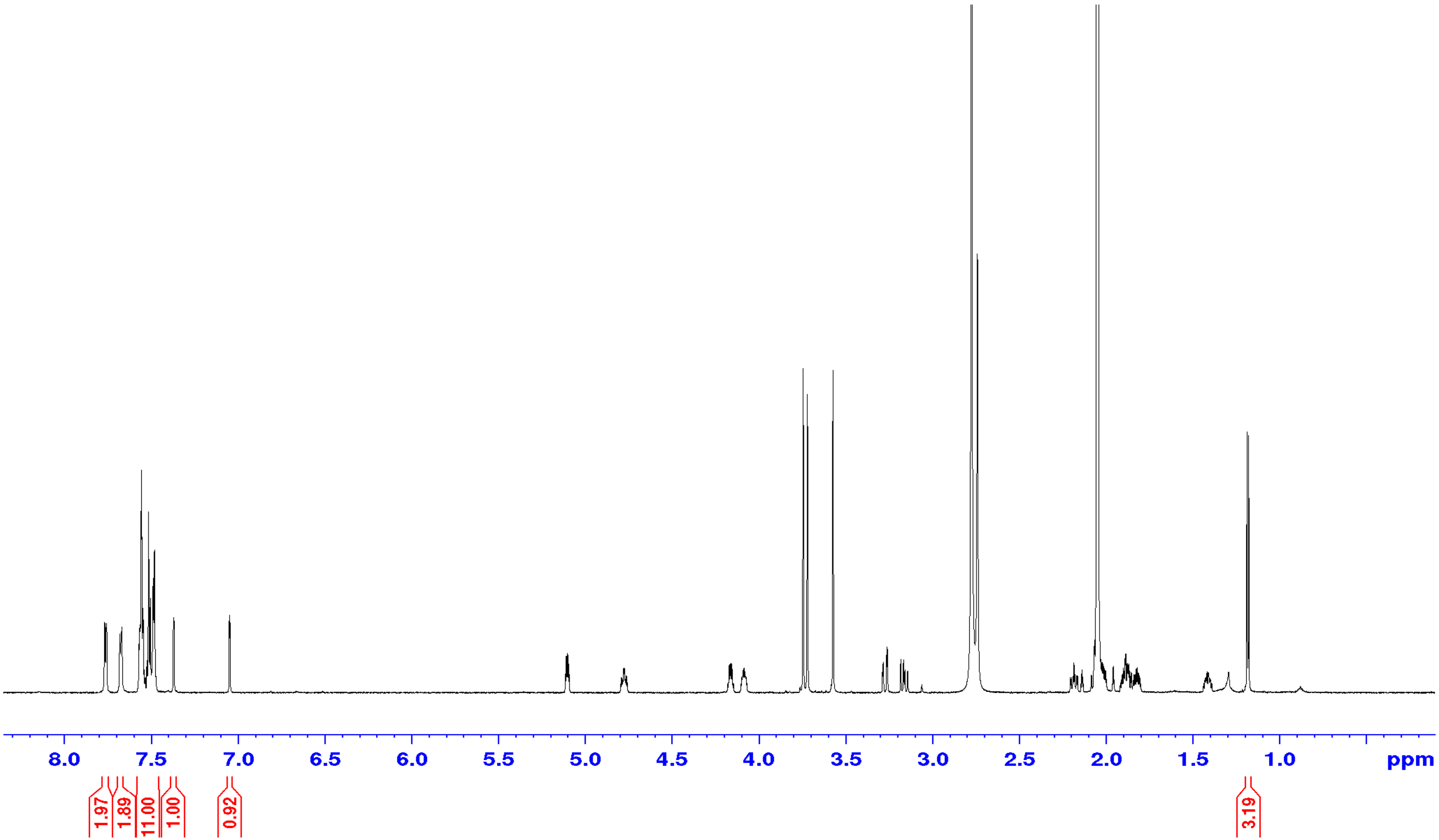

Figure S54. ^{1}H NMR spectrum for MTPA-2b (R)

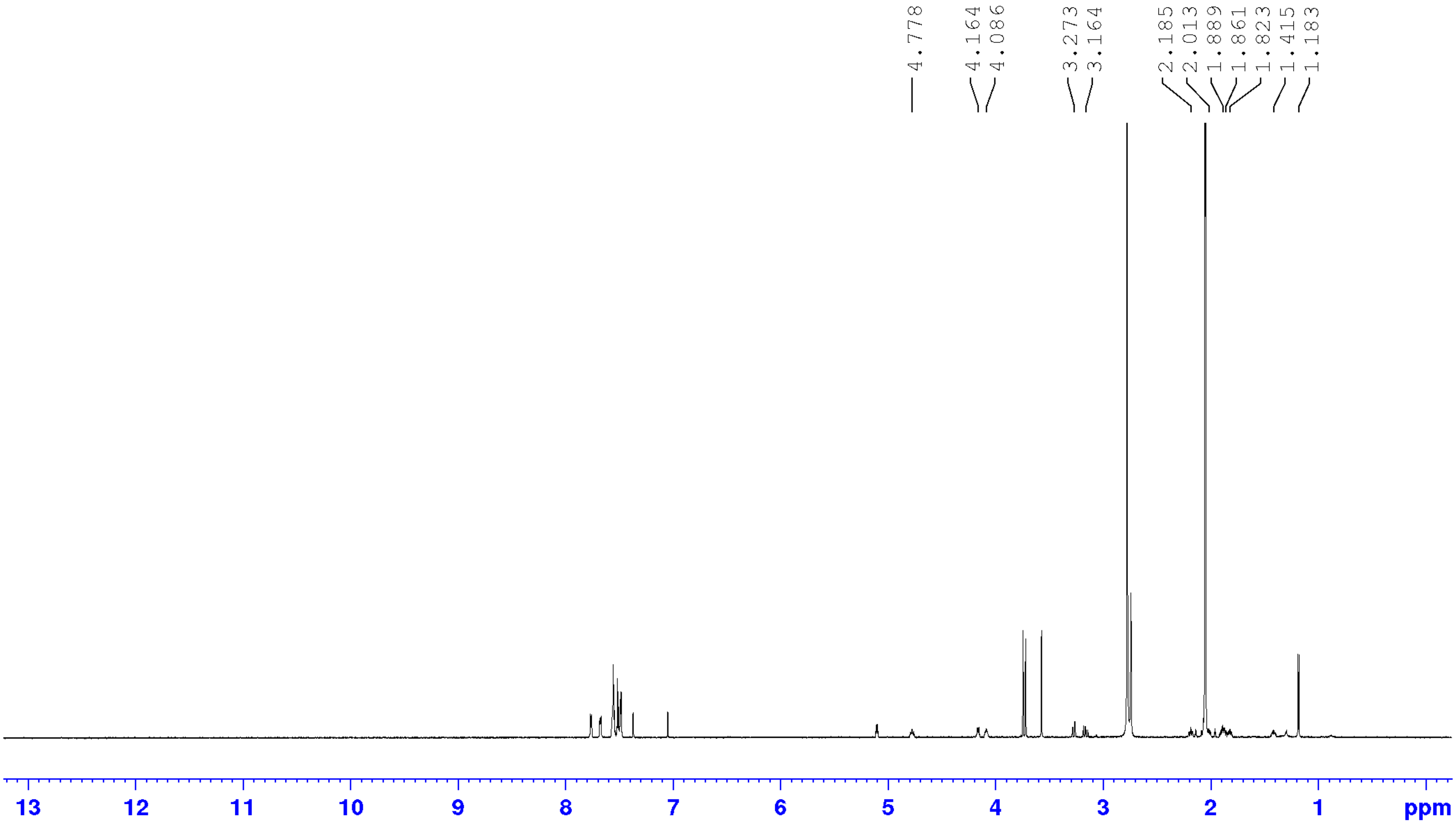

Figure S55. COSY spectrum for MTPA-2b (R)

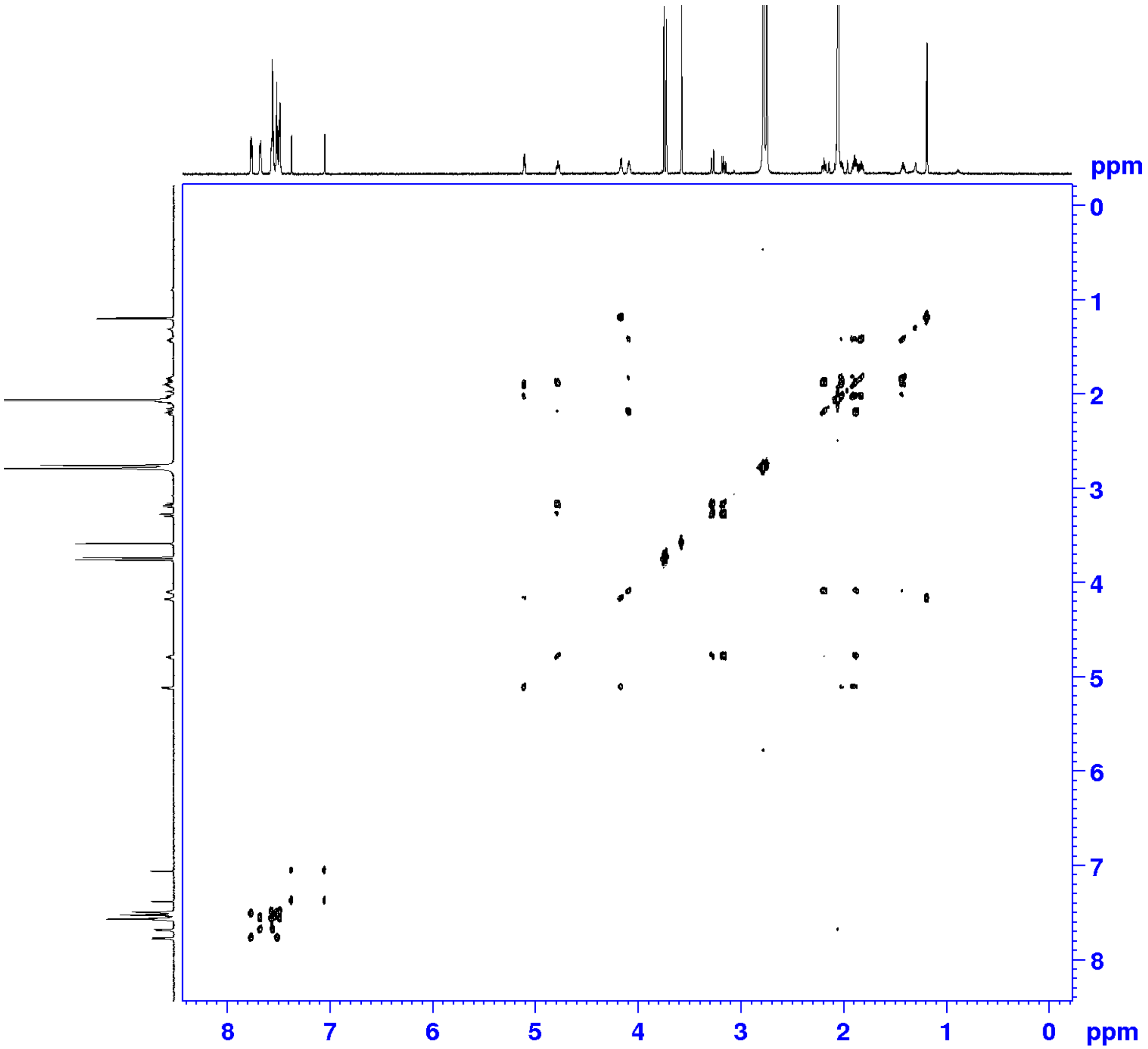

Figure S56. HSQC spectrum for MTPA-2b (R)

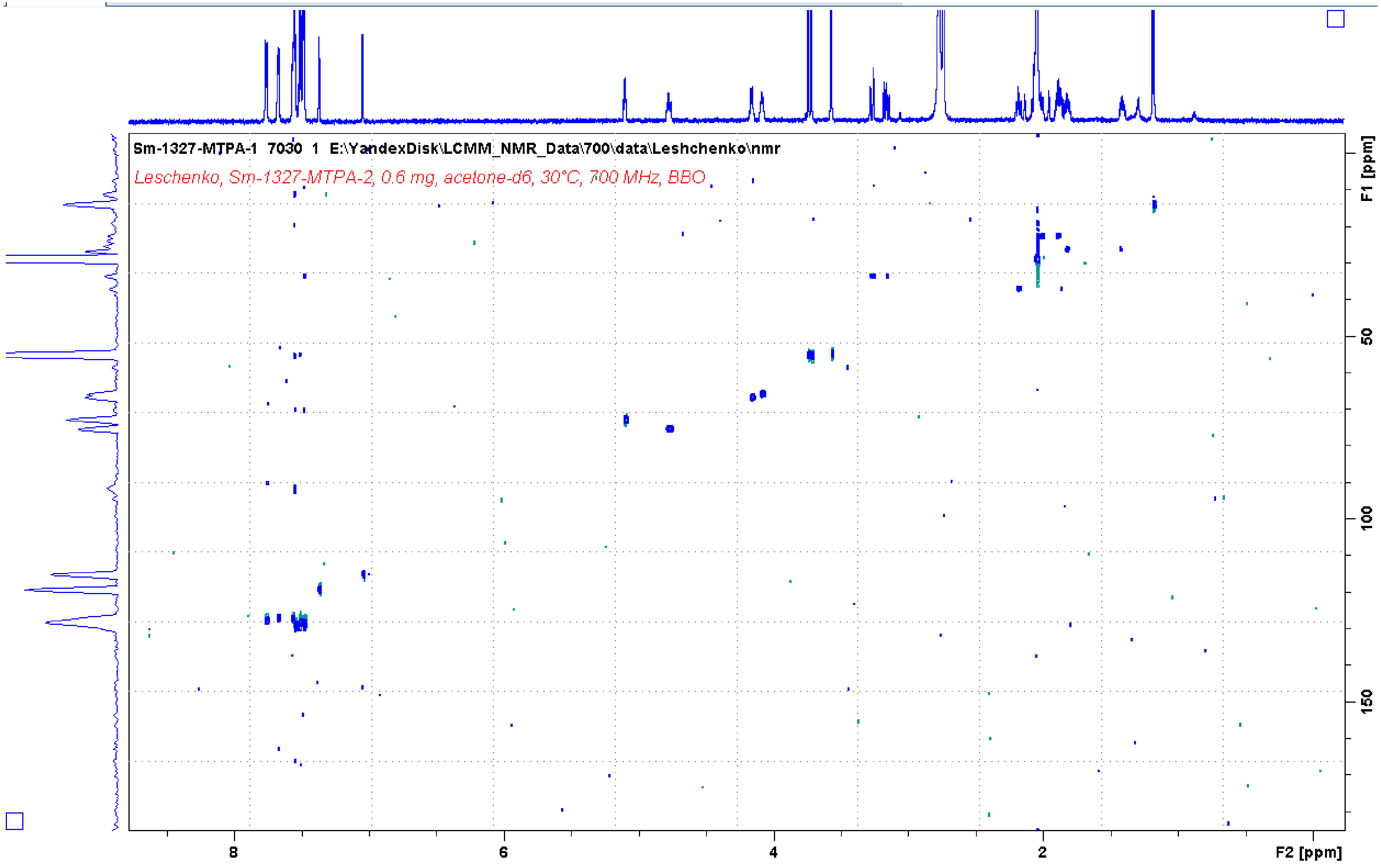

Figure S57. Scheme of isolation compounds 1-6.

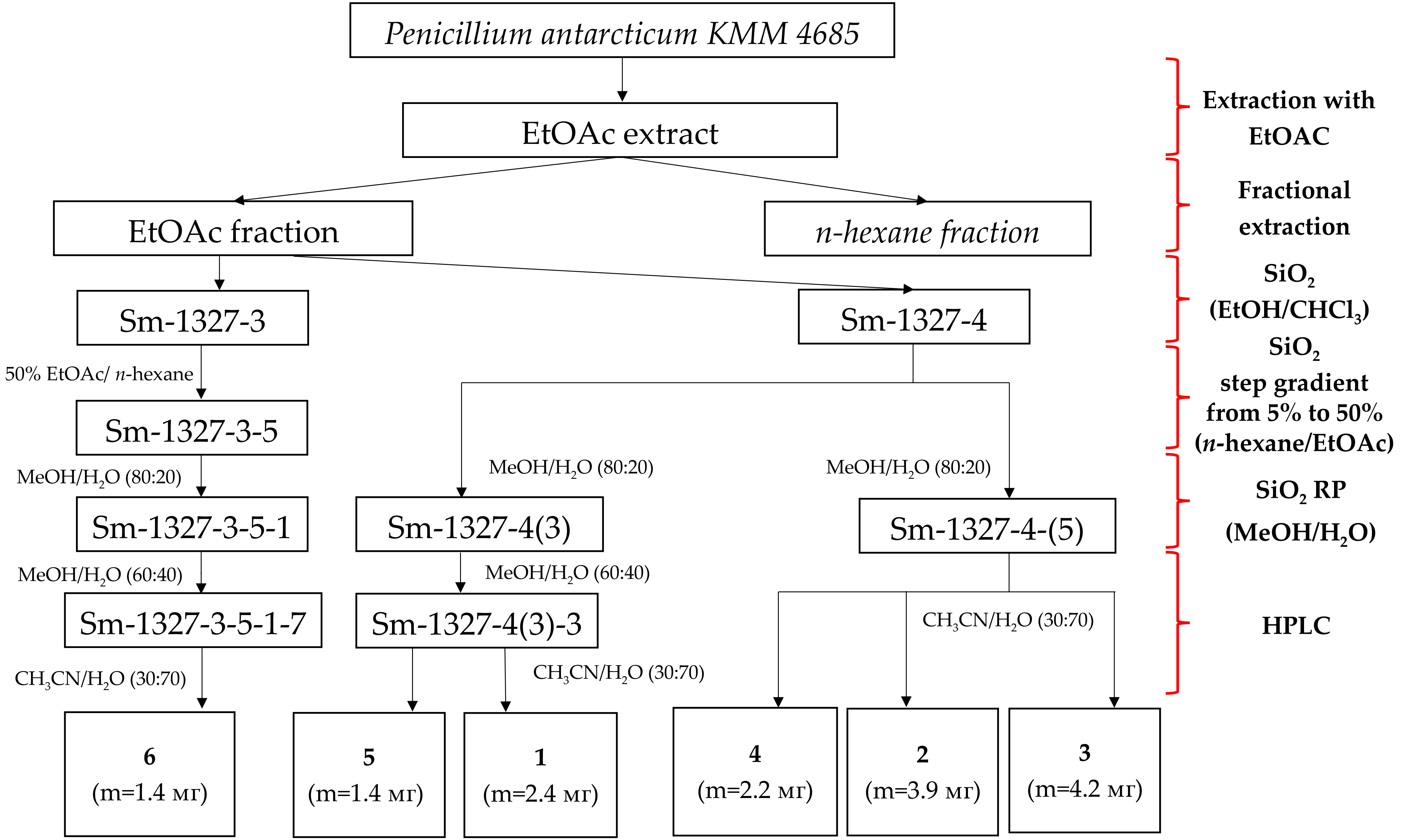

Figure S58. ROESY spectrum for 1

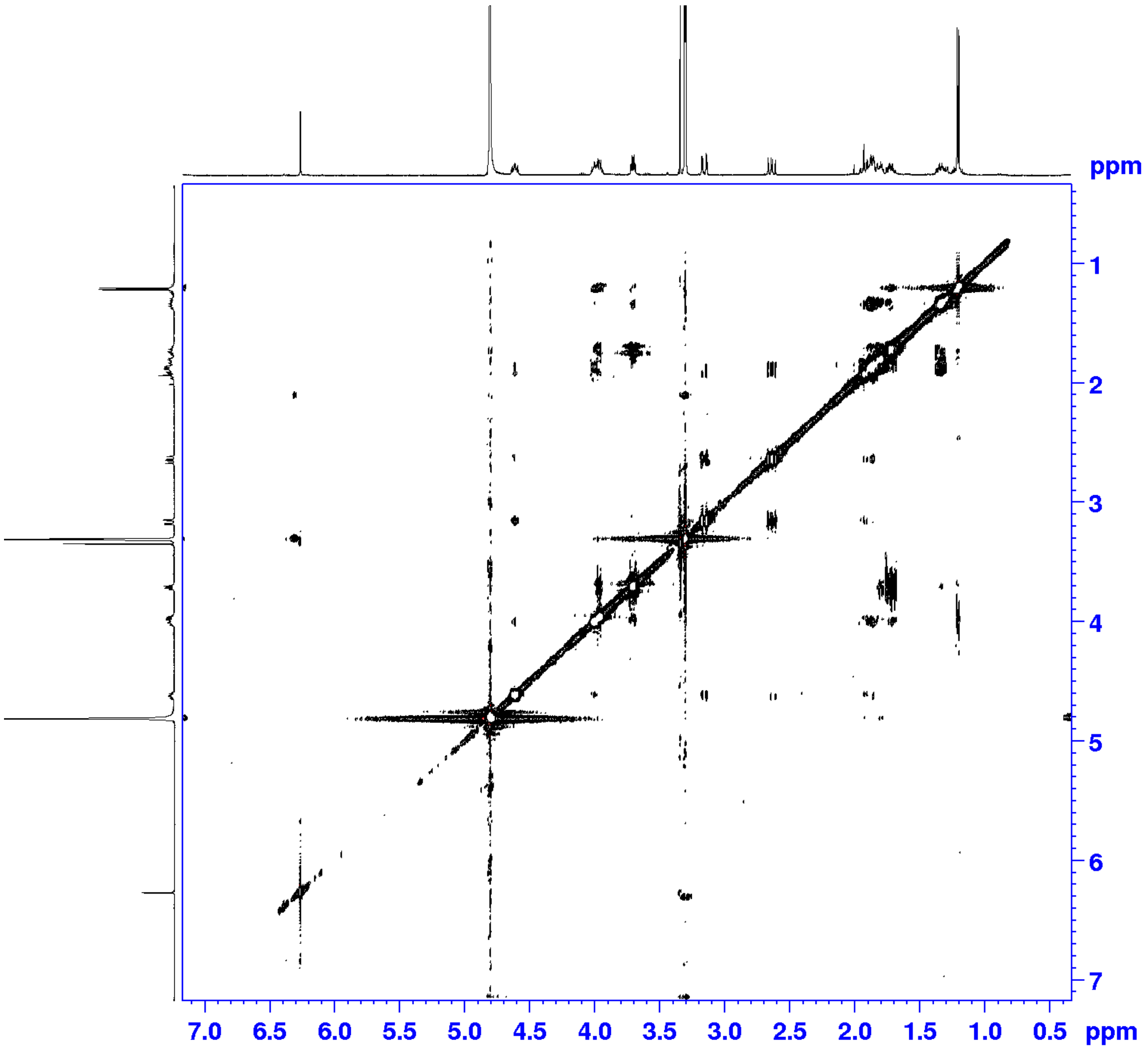

Figure S59. ROESY spectrum for 2

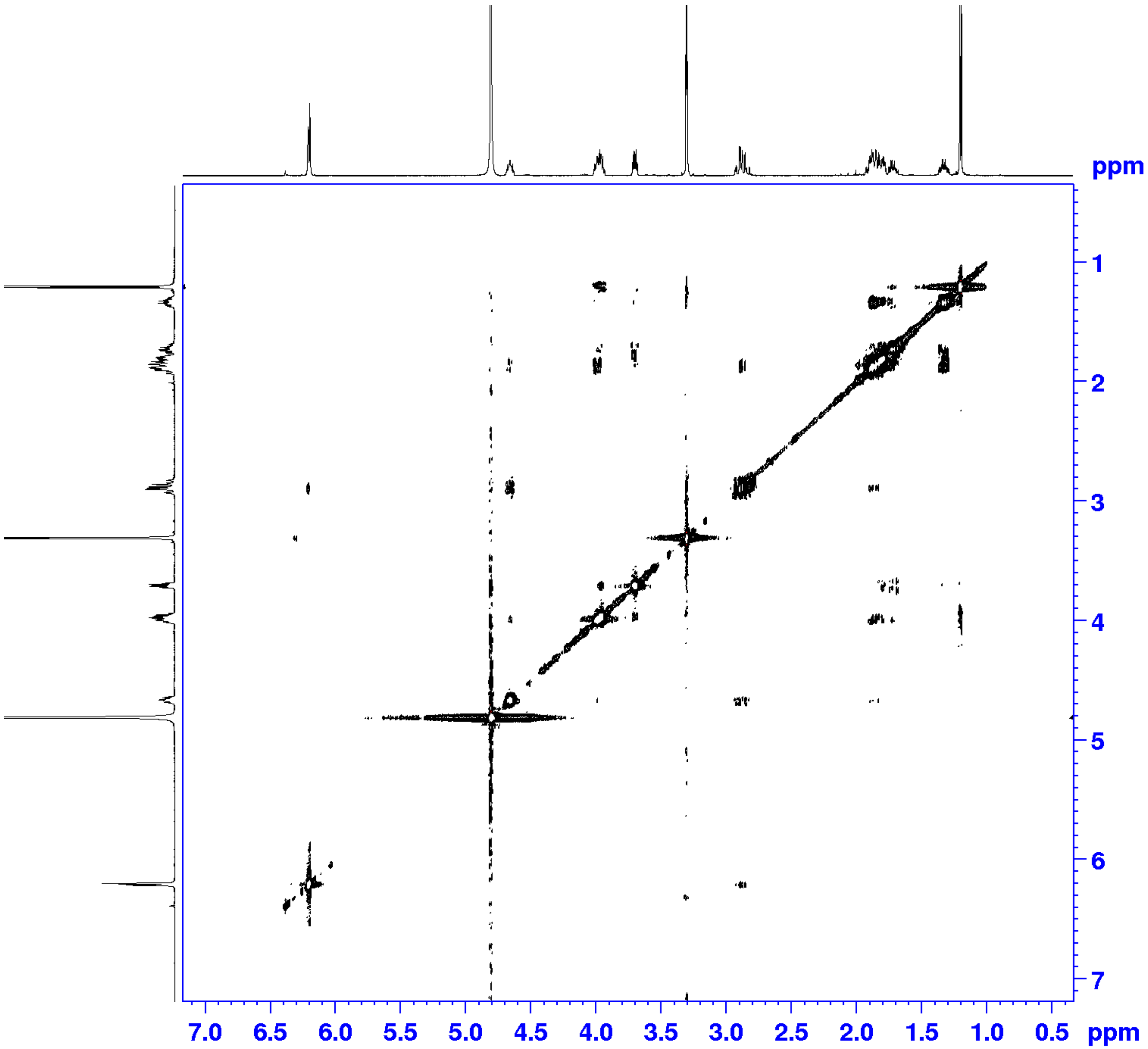

Figure S60. ROESY spectrum for 3

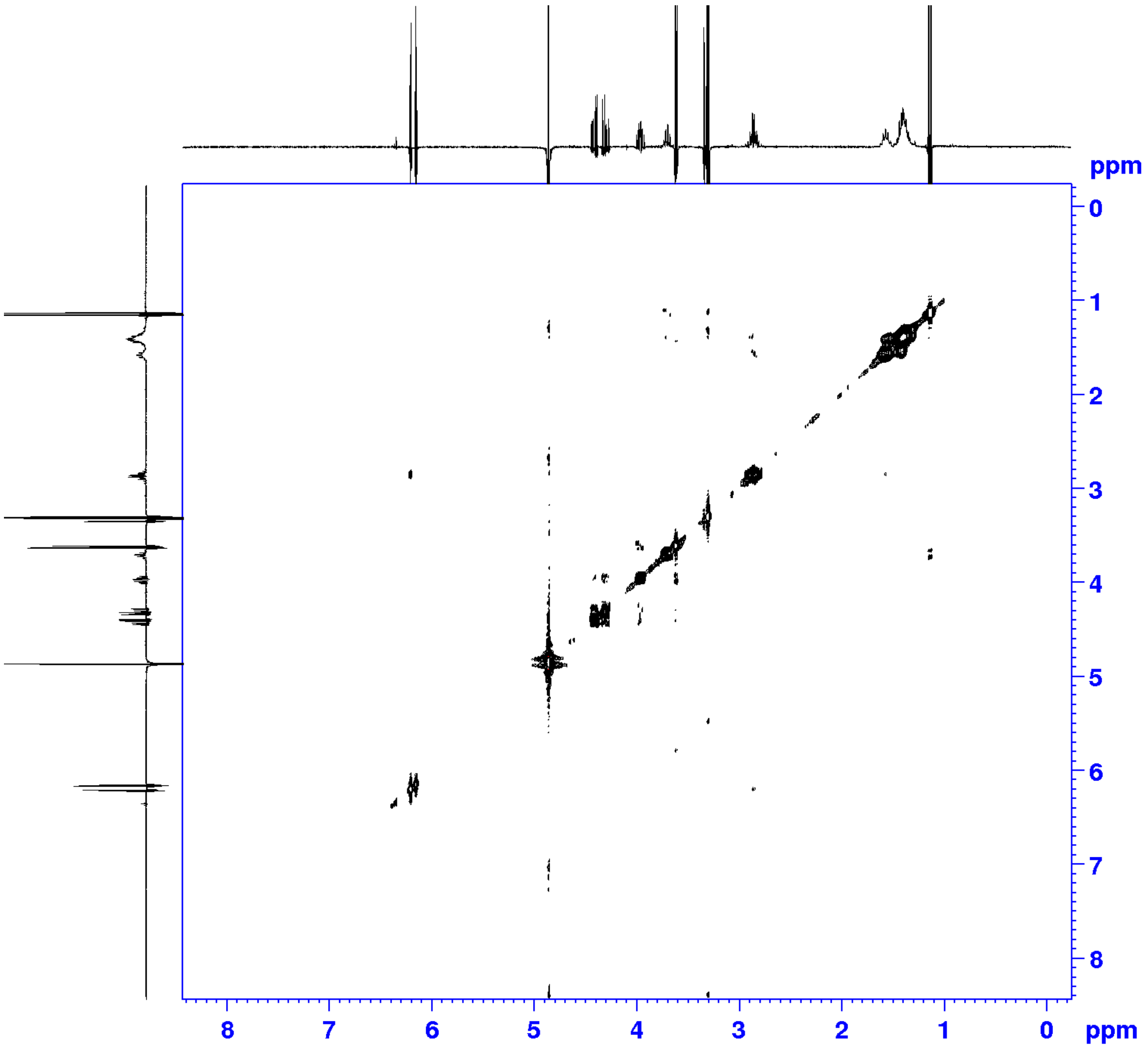

Figure S61. ROESY spectrum for 4

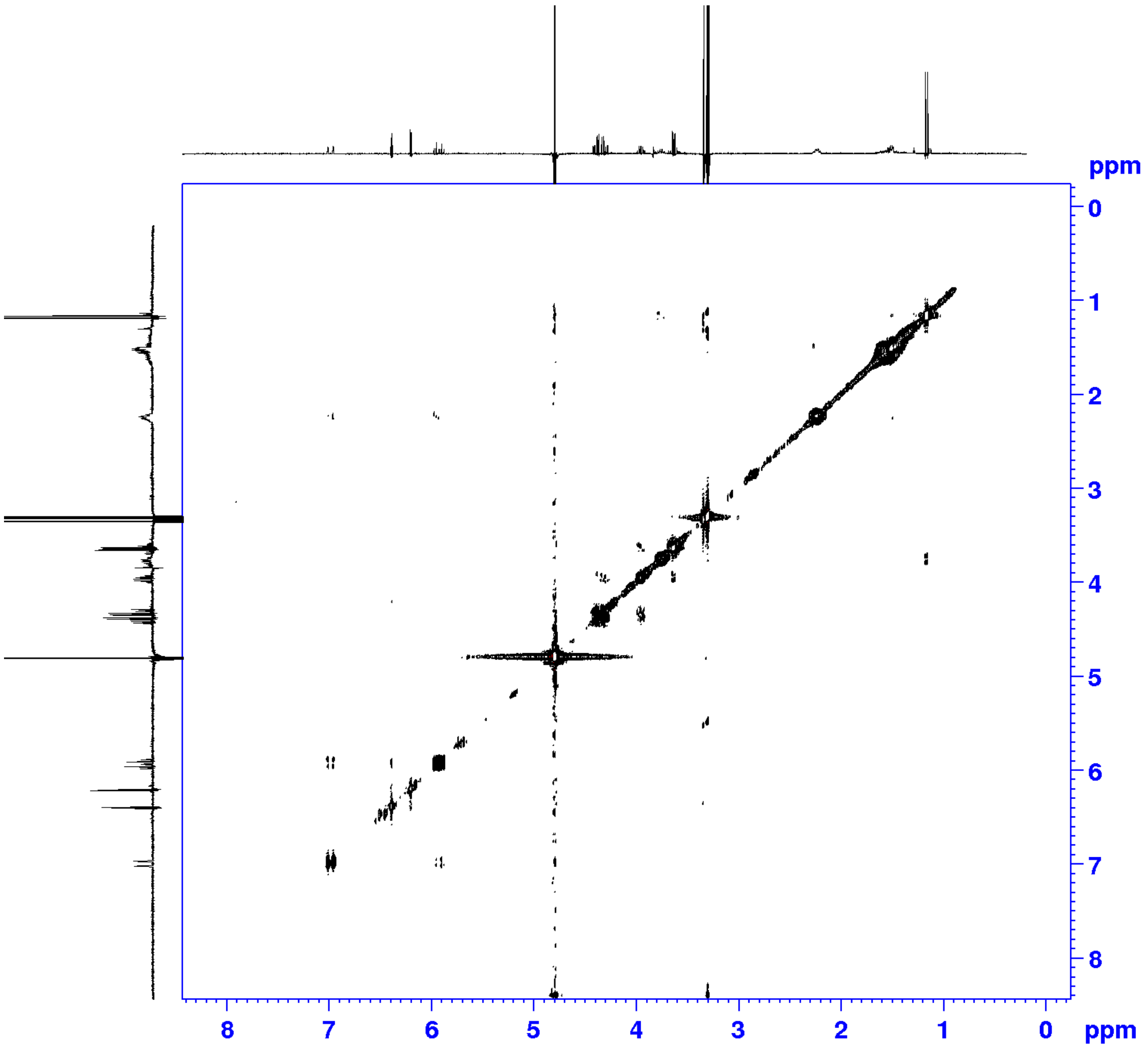

Figure S62. ROESY spectrum for 5

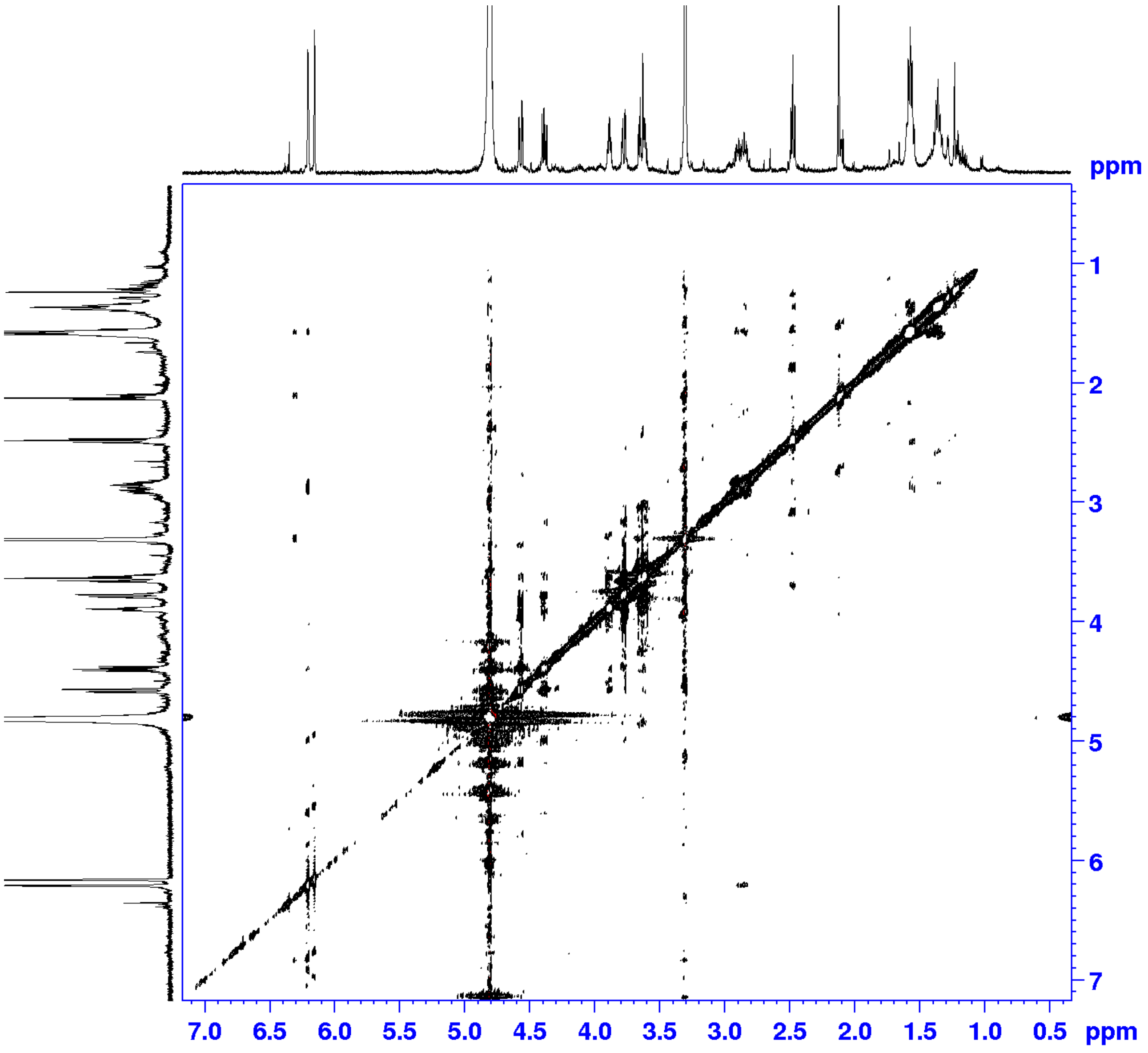

Figure S63. ROESY spectrum for 6

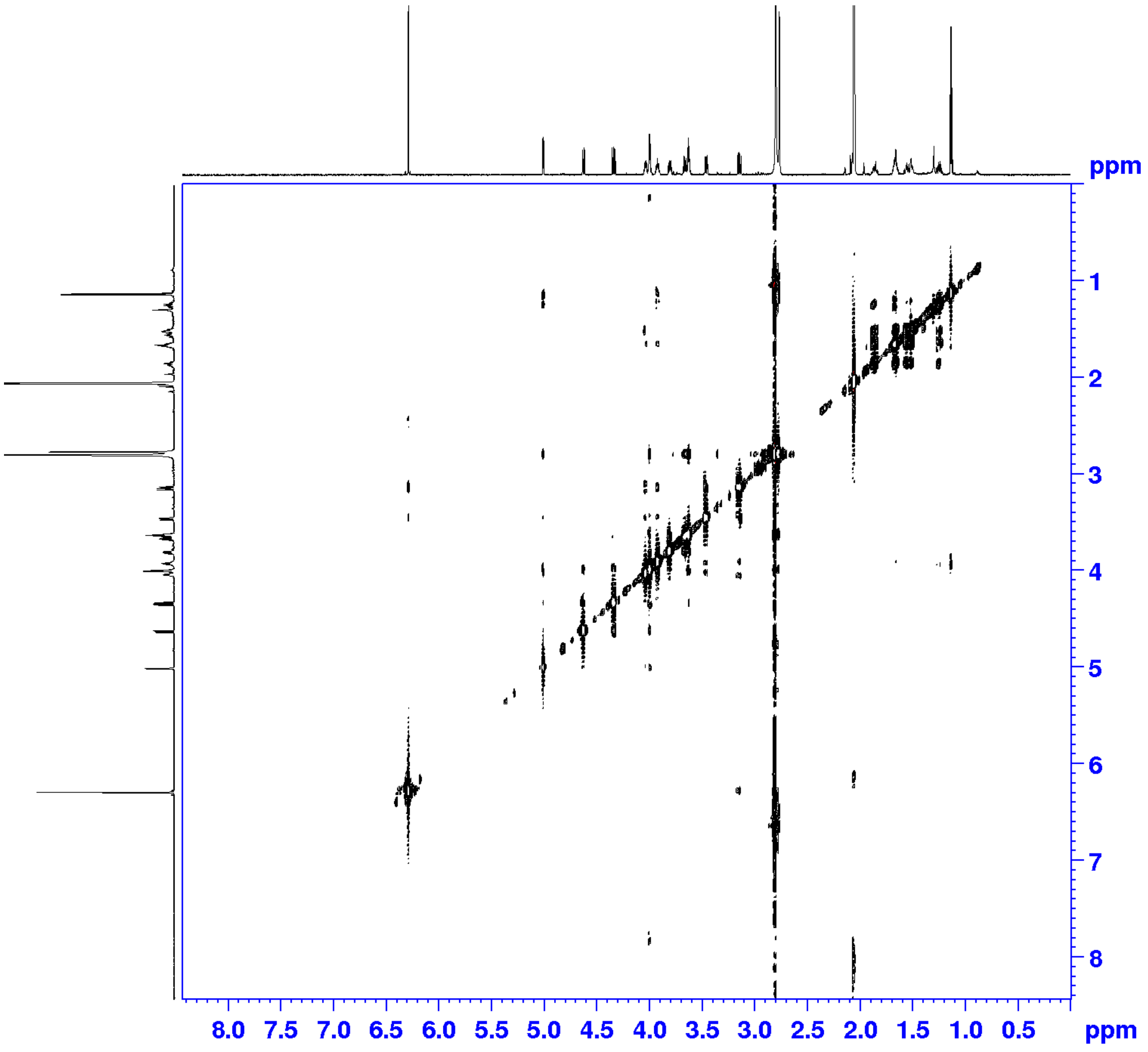

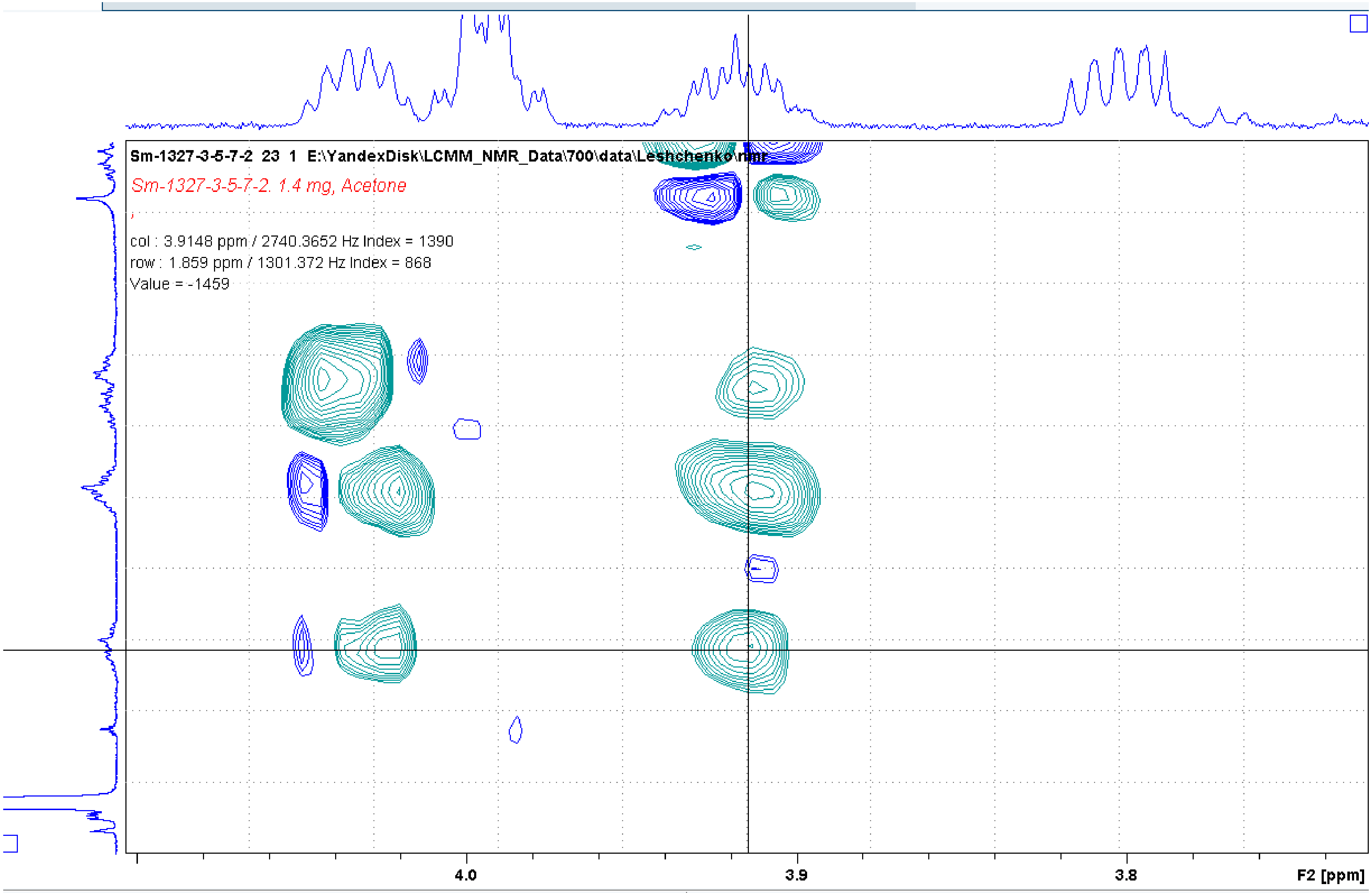
Sm-1327-3-5-7-2 23 1 E:\YandexDisk\LCMM_NMR_Data\700\data\Leshchenko\nmr
Sm-1327-3-5-7-2. 1.4 mg, Acetone
col : 3.9148 ppm / 2740.3652 Hz Index = 1390
row : 1.859 ppm / 1301.372 Hz Index = 868
Value = -1459
4.0
3.9
3.8
F2 [ppm]

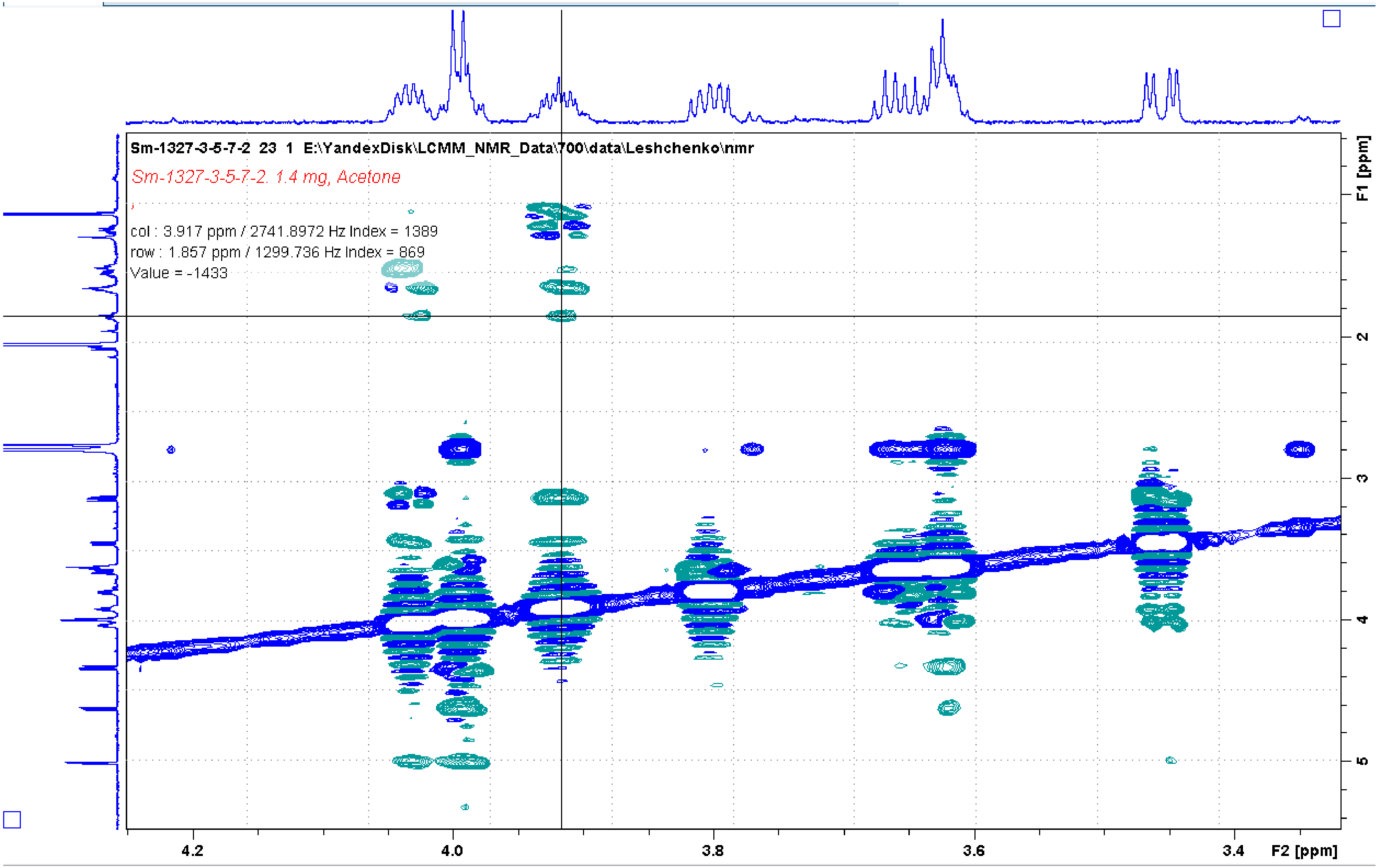

Sm-1327-3-5-7-2 23 1 E:\YandexDisk\LCMM_NMR_Data\700\data\Leshchenko\nmr
Sm-1327-3-5-7-2. 1.4 mg, Acetone
col : 3.917 ppm / 2741.8972 Hz Index = 1389
row : 1.857 ppm / 1299.736 Hz Index = 869
Value = -1433
F1 [ppm]
2
3
4
5
4.2
4.0
3.8
3.6
3.4
F2 [ppm]
